# Synthesis of Seven Indolizine-Derived Pentathiepines: Strong Electronic Structure Response to Nitro Substitution in Position C-9

**DOI:** 10.3390/molecules29010216

**Published:** 2023-12-30

**Authors:** Roberto Tallarita, Lukas Manuel Jacobsen, Benedict J. Elvers, Stefan Richter, Siva S. M. Bandaru, Jevy V. Correia, Carola Schulzke

**Affiliations:** Bioinorganic Chemistry, Institute of Biochemistry, University of Greifswald, Felix-Hausdorff-Str. 4, 17489 Greifswald, Germany; roberto.tallarita@uni-greifswald.de (R.T.); lukasmanuel.jacobsen@stud.uni-greifswald.de (L.M.J.); benedict.elvers@uni-greifswald.de (B.J.E.); stefan.richter@uni-greifswald.de (S.R.); siva.bandaru@uni-greifswald.de (S.S.M.B.); correiaj@uni-greifswald.de (J.V.C.)

**Keywords:** pentathiepines, molybdenum, polysulfides, *N*-heterocycles, *S*-heterocycles, indolizine, crystal structures, electronic spectra

## Abstract

Seven new 1,2,3,4,5-pentathiepino[6,7-*a*]indolizines were synthesized in which the pentathiepine moieties bear an indolizine backbone that is derivatized from C–H to F-, Cl-, Br-, I-, NO_2_-, and CH_3_-substitutions, respectively, in a meta position relative to the aza group on the pyridine moiety. Their preparation took place via two common steps: (i) a Sonogashira coupling between (4-substituted) 2-bromo- or 2-chloropyridines and propynyl 3,3-diethylacetal, and (ii) a ring closing reaction mediated by a molybdenum oxo-bistetrasulfido complex and elemental sulfur. The latter simultaneously facilitates the 1,2,3,4,5-pentathiepino chain/ring- and indolizine ring-formations. The fluoro derivative was addressed with 2-bromo-5-aminopyridine as the starting material via a Sandmeyer reaction. The iodo derivative was obtained from 5-bromo-2-alkynylpiridine using a metal-assisted variation of the Finkelstein reaction. The requirement to explore different reaction conditions and the varied respective yields of the final products are discussed. The influence of the distinct substitutions on the pyridine moieties, their electronic structures, and respective chemical properties was investigated through a set of spectroscopic/analytical characterizations. Intriguingly, in all cases, the nitro-substituted derivative exhibited a distinct behavior compared to the six other investigated derivatives, which was also addressed computationally. All seven new pentathiepines were crystallized, and their respective molecular structures were determined using single crystal X-ray diffraction. These structures are compared and discussed as are their respective packing patterns.

## 1. Introduction

1,2,3,4,5-pentathiepines (PTEs), as the name says, are heterocycles characterized by a seven-membered ring, five atoms of which comprise a chain of sulfur atoms while two are carbons in a C=C double bond (or, to put it more broadly, two sp^2^-hybridized carbon atoms bonded to each other). At first, synthesized unintentionally as early as 1971 [1], they remained a chemical curiosity until in 1995, derivatives thereof were discovered as natural products in marine animals belonging to the class *Ascidiacea* [2]. Exhibiting potent cytotoxic activity, their biological production was attributed to a self-defense strategy against pathogens such as bacteria [3].

Following the first recognized natural PTE named *varacin*, a whole family of molecules was found, members of which exhibit only relatively small modifications with regard to this progenitor [4]. They turned out to be targets of significant interest for pharmacological studies, since antibiotic or chemotherapeutic drugs were expected to be derived from them [5]. However, as of yet, they have rarely been adapted for clinical use. Developing synthetic strategies allowing for pentathiepine derivatization, which will result in improved properties such as solubility and biological activity, is therefore of substantial importance [6]. In addition to the therapeutic activity [7,8,9,10,11,12], it is furthermore important to understand what occurs to a drug in an organism. Biomolecules, in particular, which take care of any redox stressors, have the potential to deactivate chemical compounds before these can reach their target. Often, such biological defense systems bear sulfur atoms themselves [13,14,15,16]. Chatterji et al., in this regard, investigated in detail the dependence of the polysulfur ring stability and that of functional groups on the backbone of the pentathiepine against ethanethiol, a sulfur-based nucleophile [17]. Similar work with nitrogen-based nucleophiles followed [18,19]. Further biological studies elucidated the pharmacological demeanor of pentathiepines against glutathione (GHS) reductase and GHS itself. In all these experiments, it was often observed that the respective pentathiepine moiety was comparatively labile against reactants and even inherently, i.e., by itself.

**Figure 1:**
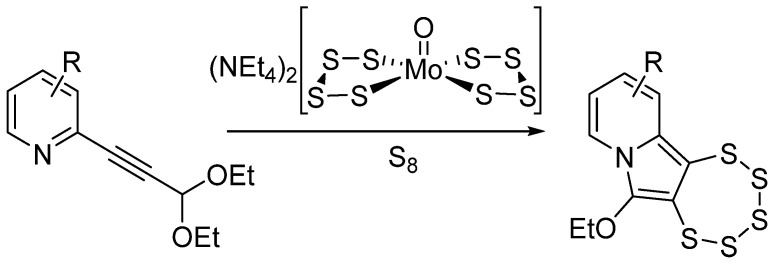
General scheme for molybdenum-mediated 1,2,3,4,5-pentathiepino[6,7-*a*]indolizine synthesis.

Despite this somehow limited pentathiepine stability, harsh conditions and/or harmful reagents were generally common features in the synthesis of artificial PTEs [20] until the serendipitous discovery of a molybdenum-mediated preparation by Zubair et al. in 2013 (Figure 1) [21]. The reactions are carried out by the application of a much milder protocol, which uses azines bearing an alkyne that carries a diethoxyacetal-protected aldehyde adjacent to the nitrogen. The result is a 1,2,3,4,5-pentathiepino[6,7-*a*]indolizine derived structure, which can easily be varied on the backbone and which is, most notably, substantially more stable than other analogues. These are characteristics which should have the potential to pave the way towards future pharmacological applications.

Another important aspect in this regard is the fact that the literature-known pentathiepines are somehow limited in their number and variation, while a larger available library would broaden, for instance, their use as monomers in electrochemical polymerization applications [22] and thereby provide an access to polymers with a wide range of characteristics. The milder protocol has the potential to change the limits of the present situation quite dramatically as was already indicated [23,24,25], and as is currently being explored by us intensively.

In previous work, the molybdenum-assisted ring formation was developed and optimized exclusively under inert gas (N_2_) conditions using dry and deoxygenated solvent. We noticed that for some precursors, this did not work as well as expected or even not at all. Contrary to the common approach, for some few cases, it was possible to successfully carry out the procedure in air and with non-dried off-the-shelf solvents. Since a synthetic procedure in air and with solvents as purchased is even more convenient and more economic, this approach was now addressed through a systematic investigation.

**Figure 2:**
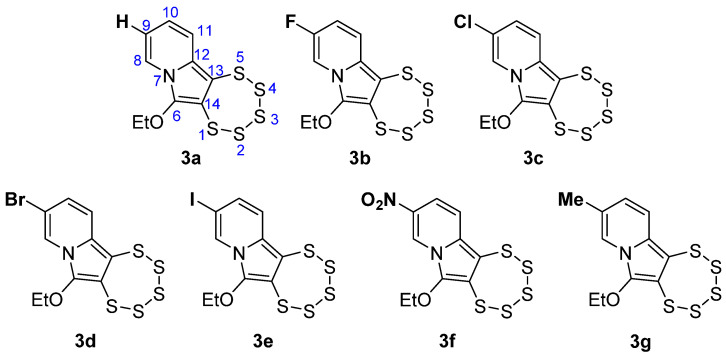
Chemical structures of the novel 1,2,3,4,5-pentathiepino[6,7-*a*]indolizines **3a**–**g,** with the numbering scheme shown for **3a** as an example.

In order to gain a better understanding of the substituent’s influence on the pentathiepines’ characteristics, a small series of compounds with varied substitutions in a single position was developed for which hydrogen, the four lighter halogens (F, Cl, Br, and I) which are also pharmaceutically relevant substituents [26], methyl, and nitro groups were used (Figure 2). Those “well behaved” functional groups were chosen because of their extensively described electronic features including inductive and mesomeric effects [27]. Furthermore, from a pharmacological point of view, they represent a key factor in drug design with regard to effectiveness and variability, widening the spectrum for target interactions and avoiding potential resistances [28]. The position to be functionalized was chosen as the para position to the alkyne in the precursor for three main reasons: (i) being far away from where the ring formation takes place, so that any steric impairment of the PTE formation can be excluded, (ii) an electronic fine-tuning from that position was expected to be relatively more independent from other effects [29], i.e., pure, and (iii) the β position to the aza atom in pyridines is more accessible to modifications and more synthetically reliable to work with [30]. The compounds were comprehensively analyzed using a range of methods such as ^1^H and ^13^C NMR, UV-vis, IR spectroscopy, and APCI mass spectrometry and elemental analyses in order to carry out a detailed comparison with regard to the effects that the substituents actually have.

## 2. Results and Discussion

Please note, the acronym PTE, more generally used for pentathiepines, will be used in the following section to indicate more specifically the seven compounds of this study; it refers to the general composition of 6-ethoxy [1,2,3,4,5]pentathiepino[6,7-*a*]indolizine with (**3b**-**g**) or without (**3a**) a substituent in the position C-9.

### 2.1. Synthetic Procedures

**Figure 3:**
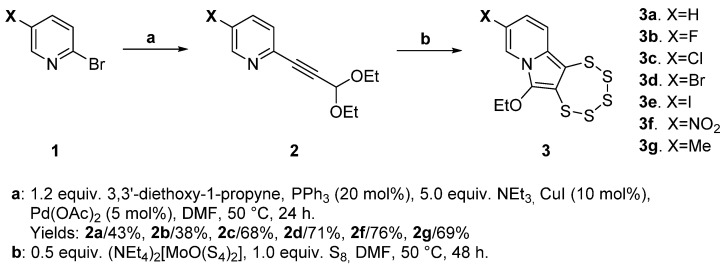
Synthetic pathway for pentathiepines consisting of a Sonogashira cross coupling followed by molybdenum mediated ring-closure. N.B., for **3e,** the sequence deviates, as **2e** is derived from **2d** (in ca. 94% of the yield) with an additional synthetic step (see below). Yields of **3** for procedures in air and under inert atmospheres are provided in Table 1 in Section 2.1.1.

The streamlined and uncomplicated protocol for the synthesis of 6-ethoxy-[1,2,3,4,5]pentathiepino[6,7-*a*]indolizines (PTEs) starts with a Sonogashira cross-coupling reaction, followed by the molybdenum-mediated ring-closing process, as illustrated in Figure 3.

Pyridines bearing either a hydrogen or a Cl-, Br-, Me-, or NO_2_-substituent at their position C-5 were commercially available for reasonable prices. The need for additional steps for the F- and I-derivatives goes back to the unavailability of respective precursors; hence, **1b** and **2e**, had to be prepared in advance.

Details of the synthesis of 5-fluoro-2-bromopyridine (**1b**) according to a literature procedure [31] are available in Section 3.2.3 and in the Supplementary Materials.

**Figure 4:**
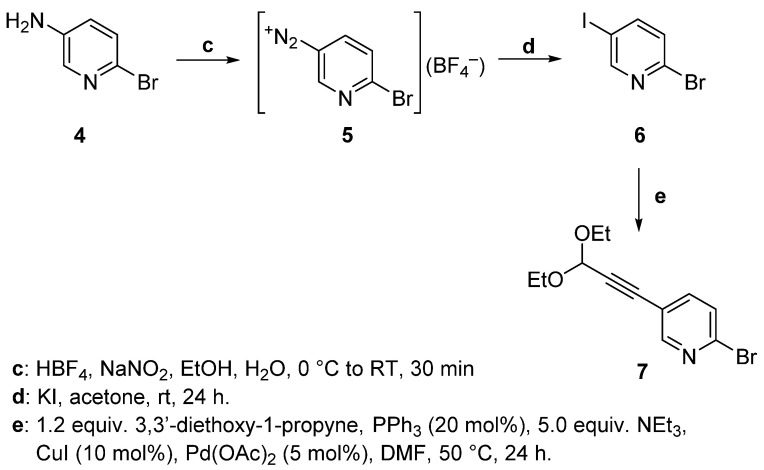
Attempted synthesis of **2e** via a diazonium salt intermediate followed by a Sandmeyer reaction [31], leading to undesired product **7** instead.

5-iodo-2-alkynylpyridine (**2e**) was derived from 5-bromo-2-alkynylpyridine (**2d**) via a metal-assisted variation of the Finkelstein reaction. A halogen substituent in *ortho* position to the aza group (such as Br in **4**–**6**, Figure 4) is substantially reactive. Yet, iodine substituents are typically more labile than their lighter congeners. Considering both of these factors, an attempt was still made to obtain 3-iodo-6-bromopyridine via the Sandmeyer reaction route (as was successful for the fluorine derivatives) followed by Sonogashira coupling. This endeavor, however, was unsuccessful, as the subsequent Sonogashira coupling occurred exclusively at the C–I bond as shown by MS analysis, with signals exhibiting the characteristic bromine pattern. 

**Figure 5:**
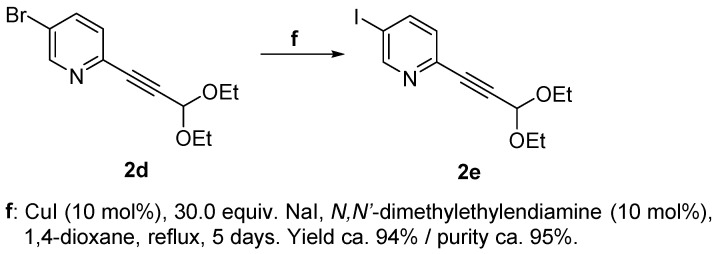
Nucleophilic substitution of bromine by iodine via a metal-assisted Finkelstein reaction.

Therefore, the order of events was reversed and compound **2d**, which had been previously prepared for the synthesis of **3d**, was used as the starting material. A nucleophilic substitution of bromine by iodine was carried out following, with some minor modifications, the procedure described by Klapars et al. [32] (Figure 5) for a copper-assisted Finkelstein reaction [33]. Both the starting material and the product exhibited the same retention factor (*R*f) in TLC (thin layer chromatography); therefore, the reaction could not be monitored using TLC but was tracked via mass spectrometric analysis in short time intervals. The conditions outlined in the literature procedure proved insufficient to drive the reaction to completion. Even under more rigorous conditions, the highest conversion achieved did not surpass the 95%/5% I vs. Br ratio in the products. A total of 30 equivalents of NaI were employed, and the reaction was replicated twice with the same batch. Eventually, the desired 9-iodo-substituted PTE could be isolated with minor contamination, but to achieve a fully satisfying complete purification (i.e., without any residual starting material or other contamination), it will be necessary to explore alternative synthetic routes in the future.

#### 2.1.1. Synthesis of 1,2,3,4,5-Pentathiepino [6,7-*a*]indolizidines **3a**–**e**

The molybdenum-mediated synthesis of PTEs was methodically developed and optimized under anhydrous conditions under an inert atmosphere (N_2(g)_). In numerous syntheses of pentathiepines in the course of various studies, these conditions were continuously applied without exception. In various synthetic contexts of PTEs, this protocol was, without exception, continuously applied and typically led to success, albeit at times with relatively lower yields. Considering previous studies, the yields in pentathiepine synthesis generally display a substantial variability from disappointingly low to appreciably high, which is not predictable [20,34]. For the majority of PTEs in this study, the addition of a molybdenum complex and elemental sulfur to a solution of the alkyne precursor in dimethyl formamide (DMF) at 50 °C was indeed initially and successfully applied. When attempting to prepare the nitro derivative (**3f**), the reaction resulted in zero yield, which could not be overcome in any of the several trials that followed in which conditions were varied, such as the temperature, solvent, and/or duration. Eventually and out of desperation, it was decided to try one more time, but in air instead of inert gas. Surprisingly, this turned out to be successful. The preparation resulted in a moderately high, and in this context, rather satisfying yield (73%) of the targeted nitro-substituted pentathiepine **3f**. This species has a notably distinct color (red) compared to all other pentathiepines prepared by our group as of yet (typically yellow to orange). This suggests that **3f** has a substantially different electronic structure compared to the other pentathiepines, and implies that the latter may be the reason for the distinct behavior during synthesis. Since working in air and with an off-the-shelf solvent (i.e., neither dried nor distilled) is far more convenient and economical than going through the procedures for inert gas/dry solvent preparations, in the present study, it was systematically tested which condition resulted in better yields depending on the substituent employed. Ideally, it would have been possible, based on the consideration of the mesomeric and inductive influences of the substituents, to predict the conditions with higher chances of success. However, there was no unambiguous trend observed for the substituents in accordance with their apparent electronic influence on the molecule (Table 1). In one case, synthesizing in air worked slightly better (Br/**3d**), in all others except for the nitro derivative (**3f**), it was the opposite or irrelevant (**3e**). The conclusion is that going for inert conditions has most often better chances of receiving higher yields and will continue to be the method of choice. Most notably, the nitro derivative is clearly an exception, and it is not unlikely that here the reaction goes through a distinct mechanism, which is still being investigated.

**Table 1:** Yields obtained under different reaction conditions in a systematic investigation of transformations of **2**→**3** (see also Figure 3).

Yields (%) for	Air/Commercial DMF	N_2_/Dry DMF
**3a**	34	45
**3b**	5	11
**3c**	22	31
**3d**	68	38
**3e**	70	70
**3f**	73	0
**3g**	21	32

Note that for the PTE with an iodine substituent (**3e**), the nucleophilic substitution reaction from **2d** to give **2e** does not proceed to completion. This results in the persistent presence of a minor impurity, which is the bromine species **3d**. In consequence, the yield was assessed using quantitative nuclear magnetic resonance ^1^H-NMR spectroscopy. The integration of signals from the starting material and the final product facilitated an effective evaluation of the yield. The proton between the aza group and the substitution in position C-9 of the pentathiepine was chosen as a reference proton. It is strategically positioned on the lateral downfield area of the spectra and thereby, farther away from potential coalescence-based interferences. Upon evaluating the T_1_ relaxation times associated with the bromine-containing species (**2d** and **3d**) and comparing the literature data [35] on the decay times of protons adjacent to C–X in aromatic systems to ensure that the instrument’s delay time D_1_ adequately covered the T_1_ of the iodine derivative, a D_1_ value 10 times higher than the bromine’s T_1_ was employed to measure the spectra of the iodinated moiety (typically, in a standard analysis, D_1_ is 5 times higher than T_1_). All data points of the free induction decay (FID) signal collected were utilized for the construction of the spectrum, so the complete information for the final spectra conversion was considered (i.e., no information omitted), whereby the determined yields are unambiguous.

As a rudimentary test for their thermal stability, the melting points of all seven pentathiepines were determined. Indeed, none of them showed any decomposition at temperatures up to their melting points in the range of 107 °C (**3a**) up to 179 °C (**3b**). This further supports the fact that this specific family of indolizine-derived pentathiepines bearing an adjacent ethoxy substituent exhibits a superior stability compared to the majority of more chemically simple PTE derivatives reported in the literature.

#### 2.1.2. Attempting a Finkelstein Reaction with **3d**

**Figure 6:**
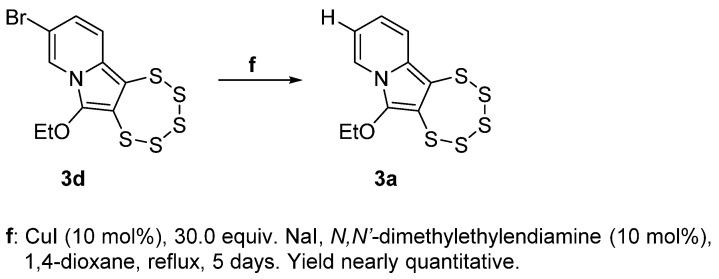
Failed attempt of a metal-assisted Finkelstein reaction using **3d** as starting material (resulting in **3a** instead of the targeted **3e**).

For accessing the iodine derivative **3e**, a metal-assisted nucleophilic substitution was attempted with **3d**. Unfortunately, the desired product was not obtained; instead, a formal dehalogenation process took place, resulting in the complete conversion to the unsubstituted (H bearing) derivative **3a** (Figure 6) with a remarkable purity and the absence of any discernible byproducts. This behavior can likely be attributed to the substantial electron deficiency of the pyridinic ring, resulting in deactivation toward the oxidative addition of the C–Br bond to the metal and also compromising the stability of the C–Cu(III)–Br intermediate itself [36].

Notable in this context is the unexpected tolerance of the pentathiepine species, despite pentathiepines being generally relatively sensitive. Here, quite extreme reaction conditions were applied without the decomposition of the pentathiepine moiety, comprising the elevated boiling temperature of dioxane (101 °C), the utilization of a nitrogen-based base and nucleophilic ligand [18], but above all, copper. This robustness of the PTE against the metal, which is highly reactive [37], is remarkable considering the well-established chalcogenophilic characteristics of transition metals and metal ions [38]. This phenomenon is particularly relevant, as sulfur species can form stable d-metal complexes, leading to a decrease in their catalytic efficiency, usually referred to as “catalyst poisoning” [39,40]. The observation made during the failed Finkelstein attempt is therefore encouraging (despite its failure) with regard to the prospect of utilizing PTEs in the future as starting materials for further derivatization (i.e., late-stage transformation).

### 2.2. Characterization

#### 2.2.1. UV-vis

**Figure 7:**
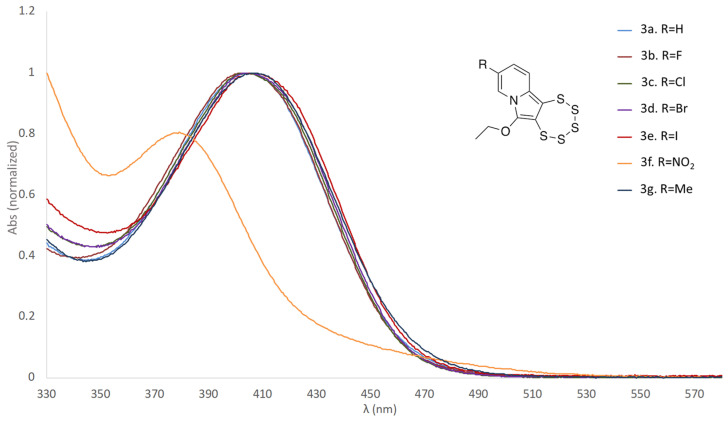
Superimposition of the normalized UV-vis spectra of the seven compounds (10^−4^ M in THF) of this study, highlighting the substantially distinct behavior of the nitro derivative **3f** and thereby the influence of this specific substituent on the relative energies of the frontier orbitals.

Since the nitro derivative **3f** exhibits a notably distinct color compared to all other pentathiepines synthesized via the molybdenum-mediated procedure, a UV-vis study was carried out with the position-C-9-derivatized compounds of the present investigation. The UV-vis spectra show a remarkable consistency in the maximum absorption wavelengths across the synthesized molecules, with the exception of the nitro derivative, which displays a considerable apparent blue shift in its absorption profile (Figure 7). The spectra of all derivatives excluding **3f** show only minimal blue or red shifts relative to each other. The nitro substituent in position C-9, therefore, quite notably influences the frontier orbital energies, whereas all other substituents merely have rather similar influences on the electronic structures of their molecules. This finding would also be in accordance with a substantially distinct behavior during preparation and supports the hypothesis that **3f**, likely, is formed via a different reaction mechanism. At the same time, it is also quite remarkable that none of the other six species show any substantial differences in their spectra, with essentially identical absorbances and very similar absorption wavelengths. Clearly, the substituents, apart from the nitro group, exert no relevant influence whatsoever on the relative energies of the frontier orbitals of the molecules.

#### 2.2.2. Computational Analysis

**Figure 8:**
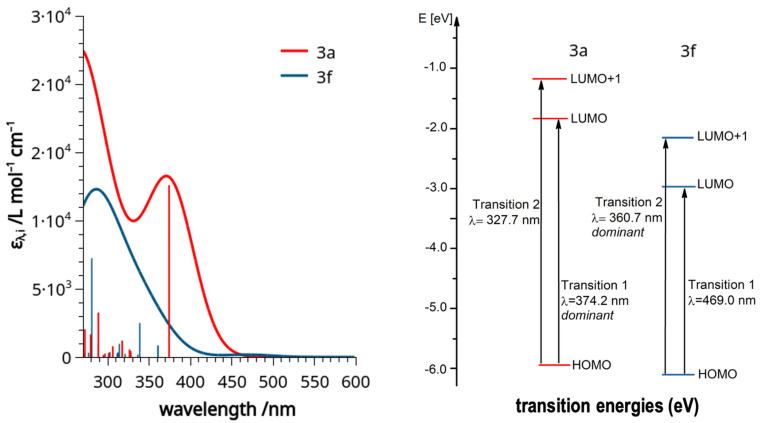
(**Left**): TD-DFT calculated UV-vis spectra on a B3LYP/def2-TZVP model basis of **3a** (red) and **3f** (blue) and the transition states the bands are based on. The calculated curves are given with a Gaussian width of 70 and a factor amplitude of 0.39 for **3a** and of 0.075 for **3f**. (**Right**): a graphical representation of the energies involved in the transitions and specifications of those that are dominant in the spectrum.

To better understand the measured experimental UV-vis data, density functional theory (DFT) calculations were performed, starting with the coordinates obtained from XRD measurements [32,33,34]. Considering the very similar UV-vis spectra of all the analyzed molecules (except for the nitro derivative **3f**), only compound **3a** was chosen for the comparison with **3f**. Compound **3a** has also the advantage of being the simplest one amongst the derivatives. A considerable deviation of the calculated spectra from the measured ones with regard to the absorption energies, due to the density functional calculation force field and base set of choice [35,36,37], was noted. However, since only a qualitative evaluation was targeted, this was not considered significant as the deviation is equally present for both computed molecules, and the same ‘shift’ is still observed between the maxima of absorptions. The difference between the HOMO and LUMO energies of **3a** and **3f** are −3.757 eV and −3.195 eV, respectively (Figure 8). The calculations show that the transitions, which are based on the electron density of the aromatic system, experience a redshift for the nitro derivative, which deprives the resonating moiety of electron density. Notably, the extinction of the HOMO–LUMO-transition, which is dominating in the spectra of **3a**–**e** and **3g**, is in **3f** very much diminished and, in fact, as the experiment shows, not practically observable. This means that we do not see a shift of an analogous band of **3f** relative to all the others, but rather the disappearance of the analogous band and concomitant emergence of another, distinct signal. This is the HOMO–LUMO+1 transition with a relatively strong extinction. The transitions to orbitals higher than the LUMO in the nitro derivative similarly experience a redshift relative to the other derivatives. The HOMO–LUMO+1 excitation of **3f** thereby just falls into the measurable wavelength range and can be observed at 360 nm. Because its extinction coefficient is quite high, this band is dominating the spectrum, and it is responsible for the distinct red color of **3f**.

**Figure 9:**
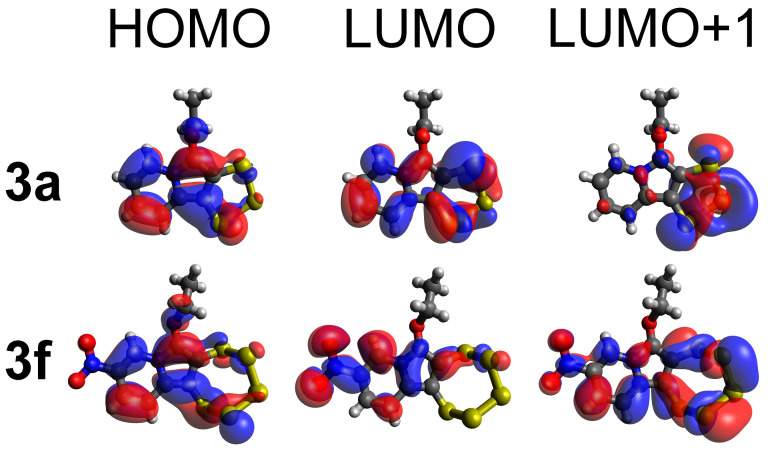
The HOMO, LUMO, and LUMO+1 frontier orbitals of the H- (**3a**) and NO_2_- (**3f**) derivatives of the indolizine pentathiepines. The strongest bands in the UV-vis spectrum arise from the HOMO–LUMO transition for **3a** and from the HOMO–LUMO+1 transition for **3f**.

From the visualization of the frontier molecular orbitals (MOs, Figure 9) it can be observed how the presence of a strongly electron-withdrawing group with a negative mesomeric (–M) effect, such as the nitro group (–NO_2_), profoundly influences the distribution of electron density on the indolizine. The two carbon atoms, belonging to both the pyrrolidine and pentathiepino rings, contribute to the frontier molecular orbitals and are notably affected by the change in the position C-9. The moiety acts as a mediator of electronic information between the two ring systems. This demonstrates that the yellow color of the typical compounds cannot be solely attributed to the polysulfide system [41], but also arises from the heteroaromatic backbone. Notably, the highest-energy occupied molecular orbitals (HOMOs) of **3a** and **3f** are virtually identical; i.e., their behaviors in a reaction with an electrophilic reaction partner should be relatively similar. The lowest-energy unoccupied molecular orbitals (LUMOs), in contrast, are rather distinct, with the orbitals distributed essentially throughout the entire molecule, except for the ethoxy substituent, in **3a**. In the LUMO of **3f**, the nitro substituent has a substantial contribution to the orbital, while four atoms of the pentathiepine moiety have (nearly) ceased to contribute quite in contrast to **3a**. The LUMO+1 in **3a** is almost entirely centered on the pentathiepine moiety. In **3f**, the LUMO+1 is more reminiscent of the LUMO in **3a**, with a distribution throughout the entire molecule except for the ethoxy substituent with contributions of the nitro, penthathiepine and aromatic moieties. One may conclude that the observable transitions in the UV-vis experiment for **3a** (HOMO to LUMO) and **3f** (HOMO to LUMO+1) take place between orbitals of pretty similar characteristics, while the strongly electron-withdrawing nitro-substituent changes the energies of the orbitals that are involved in such a way (‘pulling in’ one nitro-group-defined orbital in between as the LUMO of **3f**) that these transitions occur at notably distinct wavelengths and also with distinct extinction coefficients. All in all, the computational analysis validates the observations in the experimental UV-vis measurements and explains the conspicuously distinct color of the synthesized nitro derivative.

#### 2.2.3. NMR Spectroscopy

**Figure 10:**
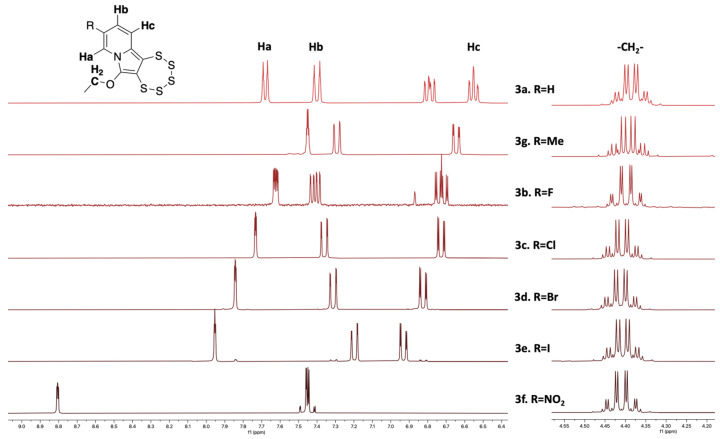
^1^H-NMR (300 MHz, CD_2_Cl_2_) spectra of the seven PTEs of this study with a zoom on aromatics and methylene (–CH_2_–) signals. The unaffected shape of the qd signal on the right stands out compared to the chemical shift and shape modification of the patterns on the left. N.B., the additional aromatic signal for **3a** goes back to the H in position 9, and in the spectrum of **3b,** some fluorine coupling is observed.

A uniform feature of the pentathiepines prepared via the molybdenum-mediated synthetic route is the presence of the ethoxy substituent adjacent to the pentathiepine moiety. The methylene group gives rise to a very specific signal in the ^1^H-NMR spectra, which is a key identifier for the formation of these pentathiepines (Figure 10) [20]. The two protons of this methylene are diastereotopic [42] and exhibit a distinctive A_3_MX signal pattern that remains consistent across various structures, regardless of the substituent at the position C-9 (or pretty much anywhere else). The reasons behind the induced geminal-CH_2_-anisochrony is related to enantiomeric pentathiepine chairlike inversion, exhaustively described in the work of Sato et al. [20,43]. The presence and nature of this notable signal evidence the common molecular architecture and the successful synthesis of the pentathiepine. This characteristic resonance might be exploited in future drug screening efforts for the elucidation of drug–target interactions and for detecting signals of metabolites using NMR [44]. In a comparison of the ^1^H-NMR spectra of all seven pentathiepines in this study, the ethoxy methylene signal has a conserved signal pattern and consistent resonance shift; i.e., it is essentially unaffected by the varied substituents in position C-9 of the pentathiepine. The aromatic regions, in contrast, are all distinct for the molecules. Again, the nitro derivative has the most distinct signals of all. With regard to the simplest PTE **3a**, the shifts of the two signals attributed to the Hb and Hc protons (see Figure 10 for denomination) are most similar to the methyl-substituted **3g**. For the four halogen-substituted pentathiepines, from the fluorine species **3b** to the iodine species **3e**, a consistent gradual downfield shifting can be observed for Hc and Ha, which apparently experience a gradual deshielding (or decrease in electron density of their molecular orbitals). For Hb, the opposite is observed, with a consistent and gradual upfield shift. For the nitro derivative **3f**, Hb and Hc merge to form an unusually shaped signal, which looks like a triplet with satellites but could not be analyzed in more detail. Again, this deviates significantly from the spectra of all other six PTEs, confirming a distinctive behavior unique to the nitro-substituted one. All aromatic hydrogens for the nitro derivative appear further (Hb), much further (Hc), or even extremely much further (Ha) downfield shifted in comparison to the six other pentathiepine derivatives. This implies that the nitro substituent deprives the aromatic system of a notable amount of electron density as would be expected from a nitro substituent, while the effect does not substantially extend beyond the aromatic system as evidenced by the minute relative methylene shifts.

#### 2.2.4. Single Crystal X-ray Diffraction Structural Analyses

**Figure 11:**
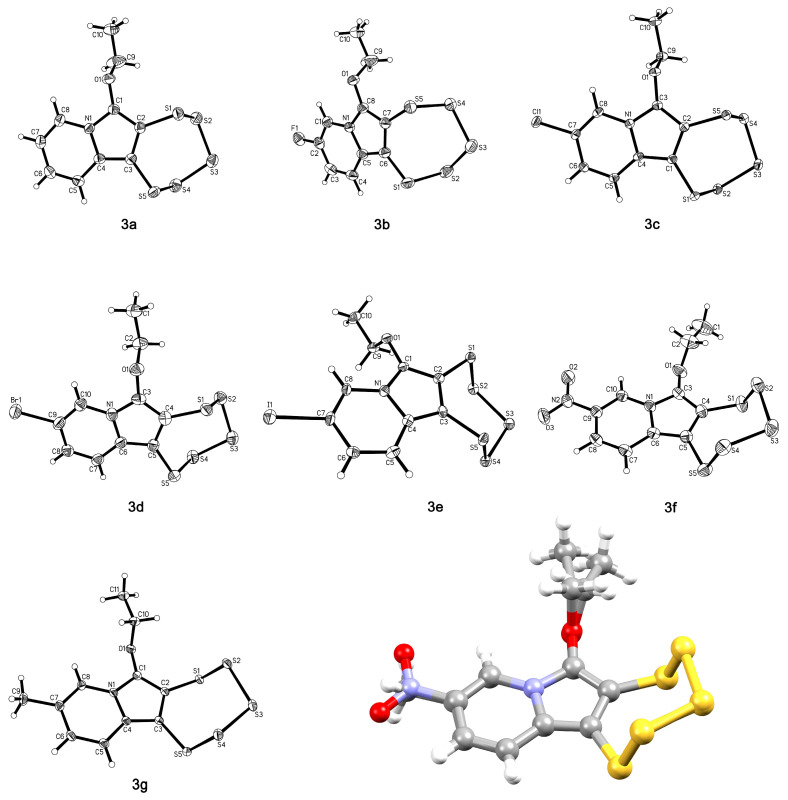
The molecular structures of pentathiepines **3a** to **3g,** and an overlay of **3f** and **3g** (bottom right). The ellipsoids of the seven molecular structures are shown at the 50% probability level.

All seven 1,2,3,4,5-pentathiepino[6,7-*a*]indolizines of this study could be crystallized sufficiently well in order to carry out single crystal X-ray diffraction structural analyses (Figure 11). Despite the variance in functional groups, all structures exhibit a nearly flawless superimposition with each other except for the varied substituent and the more flexible ethoxy group, as demonstrated by an overlay of **3f** and **3g**. These two were chosen as the most distinct in their electronic structures according to the NMR results. Notably, in all seven cases, both the ethoxy substituent and the five sulfur atom chains extend from the aromatic plane towards the same side of this plane. A search of the CSD [45] for a pentathiepine moiety fused to a five-membered ring with an adjacent ethoxy substituent gave five hits, all from our group [21,23,24]. The number of available ethoxy-substituted indolizine-derived pentathiepine molecular structures is, hence, with the present study, more than doubled. While the configuration of the pentathiepine itself is conserved throughout all examples, the ethoxy groups in the databank structures do extend towards one side of the aromatic system or the other relative to the sulfur atoms. Still, the very much conserved spatial arrangement of these pentathiepines would allow, for instance, for the building of computational models of related PTEs based on the available crystal data, and that may very well facilitate a screening of potential PTE-derived drugs in molecular docking studies even before they are actually made. In this context, the similarity of the seven structures of this study may prove to be quite valuable to the chemical and pharmaceutical communities interested in polysulfur compounds.

With regard to the metrical parameters, these are essentially unremarkable. Selected bond lengths were evaluated using the Mogul program [46] of the CSD software package (version 5.45, November 2023) where appropriate. The C–C bond lengths of the pentathiepine moiety range from 1.419(3) Å for **3b** to 1.431(3) Å for **3a**. These bond lengths strongly support the aromaticity of the indolizine five-membered ring. The chemical motif of the immediate environment of this C–C bond is with over a thousand hits far too common to carry out any meaningful analysis of the available data in this regard, while the observed C–C distances fall right into the center of the range in the deposited structures. The C–S bond lengths observed here range from 1.732(2) Å (**3a**) to 1.758(10) Å (**3e**) and all fall into the range of 1.729 Å to 1.768 Å (median value: 1.744 Å) [47,48,49] for related compounds with a ring structure consisting of five sulfur and two sp^2^-hybridized carbon atoms according to the Mogul investigation. The distances between the nitrogen and adjacent ethoxy-bearing carbon of the indolizine five-membered ring are found in between 1.361(9) Å (**3d**) and 1.378(4) Å (**3c**). As closely related structures with regard to the immediate environment of these two atoms, only 15 structures are found in the database with bond lengths ranging from 1.356 Å to 1.413 Å (median value: 1.374 Å) [50,51]. The N–C–O angles in the structures fall into the small range from 118.62(16)° (**3f**) to 120.7(7)° (**3d**). All metrical data are supplied in the Supplementary Materials, as are the crystal and refinement data.

As can be seen, the metrical parameter data are ordinary, the differences are minimal across the seven structures of this study, and trends with regard to substituent influence on the metrical parameters cannot be derived.

An interesting feature of pentathiepines is their chirality. Due to the pseudo-chair conformation of the polysulfide ring, the pentathiepines are inherently chiral [52,53]; that is, as long as the entire molecule exhibits no form of symmetry such as a mirror plane (*σ_v_*), a rotation axis (*C_n_*), or similar. The PTE compounds prepared using the molybdenum-mediated procedure, are generally chiral. They typically crystallize in space groups with symmetry elements that also generate the mirror enantiomer from the one that was refined with the sulfur chair extending into opposite sides of the aromatic plane. This results in 1:1 ratios of both enantiomers present in the cell. The energy barrier for the pseudo-sulfur-chair interconversion is around 29 kcal⋅mol^–1^, which is relatively high [43] considering that this is approximately three times the energy required for the interconversion of cyclohexane [54]. Since both enantiomers are obviously simultaneously formed during synthesis, it can be concluded that the thermodynamics and kinetics of these reactions for the two enantiomers are essentially the same, and once formed, the pentathiepines most likely do not change their stereochemistry afterwards.

**Figure 12:**
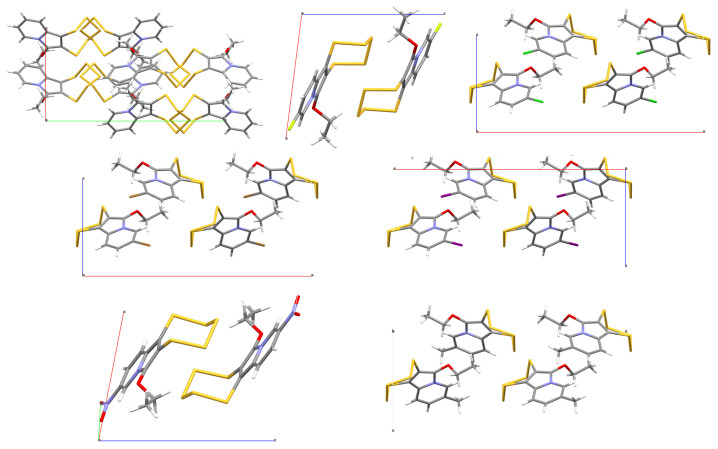
Unit cells of **3a** (top left, viewed along c), **3b** (top center, viewed along b), **3c** (top right, viewed along b), **3d** (center left, viewed along b), **3e** (center right, viewed along b), **3f** (bottom left, viewed along b), and **3g** (bottom right, viewed along b).

The space groups of the fluorine- and nitro- substituted PTEs are the same (*P*$1{;}^{\text{-}}$, Z = 2), and their packing with the two pentathiepine moieties of the molecules in the unit cell facing each other is similar (Figure 12). A minor difference is the fact that the aromatic moieties of one layer are exactly coplanar in the case of **3f,** because the nitro groups are engaged in in-plane hydrogen bonds, whereas those of **3b** are slightly spaced. In the cases of **3c**–**3e** and **3g** (Cl, Br, I, and CH_3_ substitutions), the space group is *Pca*2_1_, with four molecules in the unit cell. The packing patterns look surprisingly similar given the difference in the size/bulk of the substituents employed. The non-substituted **3a** is the only compound which crystallizes in the space group *Pbca*, with eight molecules in the unit cell, and it has a different packing pattern compared to any of the other six compounds. 

In summary, despite the extremely highly conserved molecular structures of these pentathiepines, the arrangements of the molecules in their crystals may vary quite substantially even if only a single substituent in the position C-9 is exchanged for another. However, pentathiepines with 9-substituents of a similar (isotropic or anisotropic) bulk apparently tend to crystallize in similar lattices.

## 3. Materials and Methods

### 3.1. Materials, Methods and Instrumentation

#### 3.1.1. General Experimental Procedures

The majority of experiments were carried out either under nitrogen or argon atmosphere using standard Schlenk techniques. In cases where the reactions were exposed to air, this is specifically stated. Reagents and starting materials were used as purchased without further purification. Dioxane was pre-dried over KOH and CuCl, then dried over Na before use. Dimethylformamide (DMF) was dried by refluxing under argon for 48 h over P_2_O_5_. Purification through column chromatography was conducted using silica gel VWR (particle size of 0.063–0.200 mm, 70–230 mesh ASTM). Compounds **1b** and **2e** were synthesized in accordance with the literature procedures [31,32]. Compounds **2a**, **2b**, **2c**, and **2d** were synthesized based on a similar procedure [55]. ^1^H-, ^13^C-, and ^19^F-NMR spectra were recorded on a Bruker Avance II 300 spectrometer (300, 75.5, and 282.4 MHz, respectively) using CD_2_Cl_2_ dried over activated zeolites as solvent. Chemical shifts (δ) are given in parts per million (ppm). ^1^H-NMR spectra were referenced to the peaks of residual protons of the deuterated solvent; ^13^C-NMR and ^19^F-NMR spectra were referenced to the deuterated solvent itself. Multiplicities are abbreviated as follows: s, singlet; d, doublet; t, triplet; q, quartet; m, multiplet; *J*, coupling constant (Hertz). IR spectra were recorded as KBr pellets with the FTIR spectrophotometer Shimadzu IRAffinity-1. UV-Vis spectra were recorded on a UV-3600 SHIMADZU UV-Vis-NIR spectrophotometer. Elemental analyses (C, H, N, and S) were carried out with an Elementar Vario MICRO Cube elemental analyzer. Mass spectra were acquired with an Advion Expression CMS. A Bruker Elute UHPLC system and Bruker compact QTOF-MS instrument operated with an APCI ionization source were used to measure the high-resolution accurate mass MS data. Melting points were measured with a SANIO Gallenkamp instrument. DFT calculations were performed using ORCA 5.0.3. The input xyz matrix was built using data from the SCXRD measurements. The geometries were optimized at the B3LYP (Def2-TZVP) level of theory with an empirical dispersion = GD3BJ level of DFT. To verify the stationary point, a frequency analysis was performed on experimental geometries from SCXRD templates. TD-DFT calculations were performed at the B3LYP/def2-TZVP level.

#### 3.1.2. Singe-Crystal X-ray Diffraction

Single-crystal X-ray diffraction (SCXRD) data of **3a**, **3c**, **3d**, **3e**, **3f**, and **3g** were recorded at 100 K on an XtaLAB Synergy diffractometer from Rigaku, with mirror monochromated Cu-*K*α-radiation (λ = 1.54184 Å). As the detector, a hybrid pixel array detector (HyPix) was used. Samples were mounted on LithoLoops produced by Molecular Dimensions fixed on pins produced by Hampton Research. The **3b** data were collected at 170 K on a STOE-IPDS 2T diffractometer with graphite-monochromated Mo-*K*α-radiation (λ = 0.71073 Å). The sample was mounted on a glass fiber. Absorption corrections were performed using X-Red32 and X-Shape (by STOE & Cie GmbH 2010, Darmstadt, Germany) in the case of the Mo source, or CrysAlisPro 1.171.42.61a (Rigaku OD, 2022, Tokyo, Japan) in the case of the Cu source. All structures were solved by direct or dual methods (SHELXS-2013 or SHELXT-2016/18) and refined by full-matrix least-squares techniques using the SHELXL executable and the WingX GUI. [56,57,58] All non-hydrogen atoms were refined with anisotropic displacement parameters. All hydrogen atoms were refined isotropically at calculated positions using a riding model, with their *U*_iso_ values constrained to 1.2 times *U*_eq_ of their pivot atoms for aromatic or methylene hydrogen atoms and to 1.5 times *U*_eq_ for the methyl atoms. The crystal of **3d** contained a minor racemic twin component which was addressed using the TWIN command for which the BASF parameter was refined to 0.08498. Similarly, for **3e**, there is a small minor twin component with a BASF of 0.05704. This structure also has some unassignable significant residual electron density which lies in between the oxygen (O1) and an aromatic hydrogen atom (H8) of an adjacent molecule. The location is too close to the refined molecules, so that SQUEEZE could not be applied, and at the same time, it is factually impossible that any atom resides there. The location is also not far away from the iodine and likely goes back to this very heavy element. One reflex was omitted as a clear outlier. General crystallographic, crystal, and refinement data are provided in the Supplementary Data File (Tables S1–S7). Crystallographic data were deposited with the Cambridge Crystallographic Data Centre, CCDC, 12 Union Road, Cambridge CB21EZ, UK. These data can be obtained free of charge on quoting the depository numbers CCDC 2308814 (**3a**), 2308815 (**3b**), 2308816 (**3c**), 2308817 (**3d**), 2308818 (**3e**), 2308819 (**3f**), and 2308820 (**3g**) by FAX (+44-1223-336-033), email (deposit@ccdc.cam.ac.uk), or their web interface (at http://www.ccdc.cam.ac.uk).

### 3.2. Syntheses

#### 3.2.1. Sonogashira Cross-Coupling Reaction Products

Unless otherwise specified, Sonogashira coupling reactions were conducted as follows: DMF (5.00 mL) and NEt_3_ (8.75 mL, 63.00 mmol, 10.00 equiv.) were introduced into an oven-dried Schlenk tube equipped with a side-arm stopcock and a stirring rod. The solution was degassed with nitrogen for 10 min and kept under N_2_ atmosphere throughout synthesis. The sequence of the addition of reactants was as follows: CuI (120 mg, 0.630 mmol, 0.10 equiv.), then 3,3-diethoxyprop-1-yne (1.10 mL, 7.560 mmol, 1.20 equiv.). The solution turned yellow. PPh_3_ (330 mg, 1.260 mmol, 0.20 equiv.) was added along with the appropriate 5-substituted 2-halogenopyridine (6.30 mmol, 1.00 equiv.). Finally, Pd(OAc)_2_ (71 mg, 0.315 mmol, 0.05 equiv.) was introduced, resulting in the solution’s color changing to reddish brown within seconds. The mixture was stirred at 50 °C for 72 h. The reaction progress was monitored using TLC (eluent ratio 80/20 = Hex/EtOAc, *v*/*v*, 254 nm UV lamp) and MS. Upon completion, the solvent was evaporated to dryness under vacuum using a cooling trap. The remaining residue was extracted with DCM and washed three times with saturated NH_4_Cl solution. Lastly, column chromatography was carried out (eluent Hex/EtOAc = 80/20, *v*/*v*).

2-(3,3-diethoxyprop-1-yn-1-yl)pyridine (**2a**): yield: 43%, pale brown oil, R_f_ = 0.2 (Hex/EtOAc = 80/20). ^1^H NMR (CD_2_Cl_2_, 300 MHz): δ 8.47 (ddd, 1H, *J* = 5.0, 1.8, and 1.0 Hz), 7,57 (td, 1H, *J* = 7.8 and 1.8 Hz), 7,37 (dt, 1H, *J* = 7.9 and 1.5 Hz), 7.17 (ddd, 1H, *J* = 7.61, 4.86, and 1.2 Hz), 5.38 (s, 1H), 3.75–3.50 (m, 4H), and 1.15 (t, 6H, *J* = 7.0 Hz). ^13^C{^1^H} NMR (CD_2_Cl_2_, 75.5 MHz): δ 150.5, 142.6, 136.4, 127.7, 123.7, 92.9, 84.4, 61.5, and 15.2. MS (APCI) *m*/*z*: [M + H]^+^ 206.2. HRMS (APCI/Q-TOF) *m*/*z*: [M + H]^+^ calculated for C_12_H_16_NO_2_ 206.1176; found 206.1182.

5-fluoro-2-(3,3-diethoxyprop-1-yn-1-yl)pyridine (**2b**): yield: 38%, pale brown crystalline solid, R_f_ = 0.1 (Hex/EtOAc = 80/20, *v*/*v*). ^1^H NMR (CD_2_Cl_2_, 300 MHz): δ 8.42 (dd, 1H, *J* = 2.9 Hz), 7.46 (m, 2H), 5.44 (s, 1H), 3.82–3.57 (m, 4H), and 1.23 (t, 6H, *J* = 7.1 Hz). ^13^C{^1^H} NMR (CD_2_Cl_2_, 75.5 MHz): δ 139.2, 138.9, 128.9, 123.6, 91.9, 61.5, and 15.7. ^19^F{1H} NMR (CD_2_Cl_2_, 282.4 MHz): δ 124.61. MS (APCI) *m*/*z*: [M + H]^+^ 164.2. HRMS (APCI/Q-TOF) *m*/*z*: [M + H]^+^ calculated for C_12_H_15_NO_2_F 224.1081; found 224.1083.

5-chloro-2-(3,3-diethoxyprop-1-yn-1-yl)pyridine (**2c**): reaction was conducted at room temperature in dry acetonitrile, yield: 68%, white crystalline solid, R_f_ = 0.3 (Hex/EtOAc = 80/20, *v*/*v*). ^1^H NMR (CD_2_Cl_2_, 300 MHz): δ 8.52 (dd, 1H, *J* = 2.5 and 0.8 Hz), 7.65 (dd, 1H, *J* = 8.3 and 2.5 Hz), 7.41 (dd, 1H, *J* = 8.4 and 0.8 Hz), 5.45 (s, 1H), 3.81–3.57 (m, 4H), and 1.22 (t, 6H, *J* = 7.1 Hz). ^13^C{^1^H} NMR (CD_2_Cl_2_, 75.5 MHz): δ 149.5, 140.6, 136.3, 132.3, 128.3, 91.9, 85.5, 83.4, 61.5, and 15.2. MS (APCI) *m*/*z*: [M + H]^+^ 194.2 and 196.2. HRMS (APCI/Q-TOF) *m*/*z*: [M + H]^+^ calculated for C_12_H_15_N_2_ClO_4_ 240.0786; found 240.0794.

5-bromo-2-(3,3-diethoxyprop-1-yn-1-yl)pyridine (**2d**): reaction was conducted at room temperature in dry acetonitrile, yield: 71%, pale brown waxy solid, R_f_ = 0.3 (Hex/EtOAc = 80/20, *v*/*v*). ^1^H NMR (CD_2_Cl_2_, 300 MHz): δ 8.62 (dd, 1H, *J* = 2.4 and 0.8 Hz), 7.81 (dd, 1H, *J* = 8.3 and 2.4 Hz), 7.36 (dd, 1H, *J* = 8.4 and 0.8 Hz), 5.44 (s, 1H), 3.81-3.57 (m, 4H), and 1.23 (t, 6H, *J* = 7.1 Hz). ^13^C{^1^H} NMR (CD_2_Cl_2_, 75.5 MHz): δ 151.7, 141.0, 139.2, 128.8, 121.1, 92.0, 85.7, 83.5, 61.6, and 15.3. MS (APCI) *m*/*z*: [M + H]^+^ 238.1 and 240.1. HRMS (APCI/Q-TOF) *m*/*z*: [M + H]^+^ calculated for C_12_H_15_N_2_BrO_4_ 284.0281; found 284.0284.

5-nitro-2-(3,3-diethoxyprop-1-yn-1-yl)pyridine (**2f**): yield: 76%, pale yellow oil, R_f_ = 0.5 (Hex/EtOAc = 80/20, *v*/*v*). ^1^H NMR (CD_2_Cl_2_, 300 MHz): δ 9.36 (dd, 1H, *J* = 2.7 and 0.7 Hz), 8.46 (dd, 1H, *J* = 8.6 and 2.7 Hz), 7.65 (dd, 1H, *J* = 8.6 and 0.8 Hz), 5.49 (s, 1H), 3.83-3.60 (m, 4H), and 1.24 (t, 6H, *J* = 7.1). ^13^C{^1^H} NMR (CD_2_Cl_2_, 75.5 MHz): δ 147.8, 145.7, 143.5, 131.8, 127.8, 92.0, 89.5, 82.9, 61.7, and 15.2. MS (APCI) *m*/*z*: [M + H]^+^ 237.3. HRMS (APCI/Q-TOF) *m*/*z*: [M + H]^+^ calculated for C_12_H_15_N_2_O_4_ 251.1026; found 251.1032.

5-methyl-2-(3,3-diethoxyprop-1-yn-1-yl)pyridine (**2g**): yield: 69%, pale yellow oil, R_f_ = 0.3 (Hex/EtOAc = 80/20, *v*/*v*). ^1^H NMR (CD_2_Cl_2_, 300 MHz): δ 8.31 (dd, 1H, *J* = 1.5 and 0.8 Hz), 7.38 (dd, 1H, *J* = 8.0 and 0.7 Hz), 7.26 (dd, 1H, *J* = 8.0 and 1.1 Hz), 5.36 (s, 1H), 3.74–3.49 (m, 4H), 2.24 (s, 3H), and 1.15 (t, 6H, *J* = 7.1 Hz). ^13^C{^1^H} NMR (CD_2_Cl_2_, 75.5 MHz): δ 151.0, 139.6, 136.8, 133.9, 127.1, 92.0, 84.6, 83.7, 61.4, 18.6, and 15.2. MS (APCI) *m*/*z*: [M + H]^+^ 174.2. HRMS (APCI/Q-TOF) *m*/*z*: [M + H]^+^ calculated for C_13_H_18_NO_2_ 220.1332; found 220.1339.

#### 3.2.2. Synthetic Procedures for the Copper-Mediated Finkelstein Nucleophilic Substitution

5-iodo-2-(3,3-diethoxyprop-1-yn-1-yl)pyridine (**2e**): NaI (31.48 g, 210.00 mmol, 60.00 equiv.), and CuI (0.067 g, 0.350 mmol, 0.10 equiv.) were placed in a 250 mL 3-neck round-bottom flask with a stirring rod, and the mixed powders were vacuum-dried at 120 °C for 2 h using a cooling trap to eliminate any moisture. After drying, the flask was brought to room temperature and a cycle of three times nitrogen–vacuum was applied. Then, alkyne **2d** (1g, 3.5 mmol, 1 equiv.) and *N*,*N*′-dimethylenthylenediamine (0.038 mL, 0.35 mmol, 0.10 equiv.) were added while flushing with nitrogen. Subsequently, 100 mL of dry, anaerobic 1,4-dioxane was added, and a vacuum-dried condenser was attached. The reaction was refluxed for 5 days, and the conversion of the starting material was tracked using APCI mass spectrometry. Upon completion of the reaction, indicated by no further conversion, the solvent was evaporated under vacuum. The residue was filtered to eliminate salts, washed with DCM, and then extracted three times with water. This whole process was repeated on the obtained product once more. Later, a purification via a chromatographic column using Hex/EtOAc = 80/20, *v*/*v*, was conducted, resulting in a 94% yield. The remaining contamination at ca. 5–6% consisted of unreacted **2d**. It was, as of yet, not possible to decrease it any further. Pale yellow oil, R_f_ = 0.3 (Hex/EtOAc = 80/20, *v*/*v*). ^1^H NMR (CD_2_Cl_2_, 300 MHz): δ 8.77 (d, 1H, *J* = 3.0 Hz), 7.98 (dd, 1H, *J* = 8.0 and 3.0 Hz), 7.23 (d, 1H, *J* = 8 Hz), 5.43 (s, 1H), 3.81–3.56 (m, 4H), and 1.22 (t, 6H, *J* = 7 Hz). ^13^C{^1^H} NMR (CD_2_Cl_2_, 75.5 MHz): δ 156.5, 144.7, 141.2, 129.2, 91.8, 86.0, 83.6, 61.6, and 15.3. MS (APCI) *m*/*z*: [M + H]^+^ 287.3. HRMS (APCI/Q-TOF) *m*/*z*: [M + H]^+^ calculated for C_12_H_15_NIO_2_ 332.0142; found 332.0147.

#### 3.2.3. Synthetic Procedures for Sandmeyer Reaction

5-fluoro-2-bromopyridine (**1b**): 5-amino-2-bromopyridine (5.00 g, 28.90 mmol, 1.00 equiv.) was dissolved in 10 mL of ethanol. A 50% solution of tetrafluoroboric acid in water (6.10 mL, 34.70 mmol, 1.20 equiv.) was added, lowering the reaction mix temperature to 0 °C using an ice bath. NaNO_2_ (2.20 g, 31.79 mmol, 1.10 equiv.) was dissolved in 5 mL of water and gradually added to the cooled solution. The reaction was stirred for 1 h at 0 °C. Complete conversion of the amine was monitored using TLC (Hex/EtOAc = 70/30, *v*/*v*) and the ninhydrin stain. Upon full consumption of the starting material, 50 mL of Et_2_O was added, resulting in a pale yellow precipitate, positive to the β-naphthol test. The precipitate was promptly filtered, washed with more Et_2_O, and the product, a tetrafluoroborate diazonium salt, was directly utilized for the subsequent step. It was added to 100 mL of hexane and heated at 40 °C for 24 h. A reddish color developed as gas evolved. When no more diazonium salt was detected, the reaction was stopped, and the solvent was removed under vacuum. The residue was extracted with DCM, washed three times with water, confirmed by mass, and directly used for the Sonogashira reaction. The yield reached for the crude product was 57%. MS (APCI) *m*/*z*: [M + H]^+^ 163.1.

#### 3.2.4. Synthetic Procedures for Pentathiepine and Pyrrole Rings Closing

Pentathiepine syntheses were executed side by side at the same time to facilitate a comparative analysis of the reaction yields. The reactants, including alkynes, the molybdenum complex, and sulfur, were sourced from the same batches throughout.

In the first procedure, following the method described by Zubair et al. [21], the appropriate quantities of alkynes (1.00 mmol, 1.00 equiv.), molybdenum oxo-tetrabisulfido complex (0.50 mmol, 0.50 equiv.), and elemental sulfur (1.00 mmol, 1.00 equiv.) were introduced into flame-sealed Schlenk tubes containing a magnetic stirring bar, purged of air under vacuum. Then, the system underwent three cycles of nitrogen–vacuum purging, after which 3 mL of dry anaerobic DMF was added. The resulting heterogeneous solution was stirred at 50 °C for 48 h. The reaction was stopped, and the solvent removed under vacuum using a cooling trap. The product was purified using column chromatography commencing with 100% hexane and transitioning in gradient to an increased polarity of the eluent up to a mix of 95% hexane and 5% EtOAc, *v*/*v*. The initial use of pure hexane ensured the removal of any sulfur residual from the reaction mixture. While shelf sulfur is not soluble in hexane, the sulfur in the reaction mixture is. This goes back to a change in morphology during the reaction, which is still being investigated by us. Its presence (R_f_ = 0.95) was checked via TLC with a 254 nm UV lamp.

The second procedure was performed by dissolving the three reactants with the same amounts in 3 mL of non-anhydrous DMF in ambient atmosphere. The heterogeneous solution was stirred at 50 °C for 48 h and purified as stated above.

6-Ethoxy-[1,2,3,4,5]pentathiepino[6,7-*a*]indolizine (**3a**): yield (under air): 34%, yield (under N_2_): 45%, yellow crystalline powder, R_f_ = 0.2 (Hex/EtOAc = 95/5, *v*/*v*). ^1^H NMR (CD_2_Cl_2_, 300 MHz): δ 7.76 (dt, 1H, *J* = 7.1 and 1.1 Hz), 7.48 (dt, 1H, *J* = 9.2 and 1.2 Hz), 6.87 (ddd, 1H, *J* = 9.1, 6.6, and 1.1 Hz), 6.63 (td, 1H, *J* = 6.9 and 1.2 Hz), 4.46 (qd, 2H, *J* = 7.1 and 2.5 Hz), and 1.45 (t, 3H, *J* = 7.1 Hz). ^13^C{^1^H} NMR (CD_2_Cl_2_, 75.5 MHz): δ 140.8, 131.0, 121.54, 121.31, 118.3, 112.6, 109.2, 72.7, and 15.5. UV-vis (THF) λ_max_ = 404.5 nm. IR (KBr disk, cm^−1^): ν = 2924s, 2854s, 1623w, 1538s, 1516w, 1492w, 1463m, 1362m, 1344m, 1284m, 1223m, 1025m, 996w, 886w, 858w, 739s, 663w, and 627w. mp (°C): 107. Elemental analysis calculated for C_10_H_9_NS_5_O (%): C: 37.59; H: 2.84; N: 4.38; and S: 50.17. Found: C: 38.00; H: 2.66; N: 4.27; and S: 49.57.

6-Ethoxy-9-fluoro-[1,2,3,4,5]pentathiepino[6,7-*a*]indolizine (**3b**): yield (under air): 5%, yield (under N_2_): 11%, yellow crystalline powder, R_f_ = 0.4 (Hex/EtOAc = 95/5, *v*/*v*). Due to low product yield and availability, HRMS was performed instead of elemental analysis. ^1^H NMR (CD_2_Cl_2_, 300 MHz): δ 7.63 (ddd, 1H, *J* = 5.3, 2.1, and 0.9 Hz), 7.41 (ddd, 1H, *J* = 9.9, 5.3, and 0.9 Hz), 6.73 (ddd, 1H, *J* = 10.0, 8.0, and 2.2 Hz), 4.40 (dq, 2H, *J* = 7.1 and 1.6 Hz), and 1.38 (t, 3H, *J* = 7.1 Hz). ^13^C{^1^H} NMR (CD_2_Cl_2_, 75.5 MHz): δ 155.8, 128.6, 119.8, 108.0, 107.6, 95.0, 72.8, and 15.7. ^19^F{1H} NMR (CD_2_Cl_2_, 282.4 MHz) δ 140.53. UV-vis (THF) λ_max_ = 403.5 nm; IR (KBr disk, cm^−1^): ν = 2952s, 2853s, 1743s (CF), 1651w, 1522m, 1504m, 1466m, 1362w, 1255m, 1155br, 1100m, 1014m, 929w, 868w, 814m, 787m, and 722m. MS (APCI) *m*/*z*: [M + H]^+^ 338.3. mp (°C): 179. HRMS (APCI/Q-TOF) *m*/*z*: [M + H]^+^ calculated for C_10_H_9_N_2_S_5_OF 337.9266; found 337.9275.

6-Ethoxy-9-chloro-1,2,3,4,5-pentathiepino[6,7-*a*]indolizine (**3c**): yield (under air): 22%, yield (under N_2_): 31%, yellow crystalline powder, R_f_ = 0.3 (Hex/EtOAc = 95/5, *v*/*v*). ^1^H NMR (CD_2_Cl_2_, 300 MHz): δ 7.75 (dd, 1H, *J* = 1.8 and 1.0 Hz), 7.36 (dd, 1H, *J* = 9.6 and 1.0 Hz), 6.73 (dd, 1H, *J* = 9.6 and 1.7 Hz), 4.41 (qd, 2H, *J* = 7.1 and 2.3 Hz), and 1.38 (t, 3H, *J* = 7.1 Hz). ^13^C{^1^H} NMR (CD_2_Cl_2_, 75.5 MHz) δ 140.5, 128.6, 122.4, 121.0, 119.0, 118.8, 113.8, 72.5, and 15.4. UV-vis (THF) λ_max_ = 402.0 nm; IR (KBr disk, cm^−1^): ν = 1534s, 1508w, 1491w, 1474w, 1436w, 1388w, 1368w, 1351m, 1325s, 1268s, 1228m, 1158m, 1145m, 1104m, 1056m, 1010s, 989m, 986m, 864m, 800s, 700m, and 682m. MS (APCI) *m*/*z*: [M + H]^+^ 355.5. mp (°C): 159. Elemental analysis calculated for C_10_H_8_NS_5_ClO (%): C: 33.94; H: 2.28; N: 3.96; and S: 45.29. Found: C: 33.90; H: 2.38; N: 3.72; and S: 43.04.

6-Ethoxy-9-bromo-[1,2,3,4,5]pentathiepino[6,7-*a*]indolizine (**3d**): yield (under air): 68%, yield (under N_2_): 38%, yellow crystalline powder, R_f_ = 0.3 (Hex/EtOAc = 95/5, *v*/*v*). ^1^H NMR (CD_2_Cl_2_, 300 MHz): δ 7.92 (dd, 1H, *J* = 1.7 and 0.9 Hz), 7.39 (dd, 1H, *J* = 9.5 and 1.0 Hz), 6.90 (dd, 1H, *J* = 9.5 and 1.7 Hz), 4.49 (qd, 2H, *J* = 7.0 and 2.2 Hz), and 1.46 (s, 3H, *J* = 7.0 Hz). ^13^C{^1^H} NMR (CD_2_Cl_2_, 75.5 MHz): δ 151.7, 141.0, 139.2, 128.8, 121.1, 92.0, 85.7, 83.5, 61.6, and 15.3. UV-vis (THF) λ_max_ = 406.0 nm; IR (KBr disk, cm^−1^): ν = 1618w, 1533s, 1506w, 1491w, 1436w, 1386w, 1367w, 1351w, 1326s, 1268s, 1228s, 1144w, 1131w, 1104w, 1045m, 1009s, 984m, 885m, 861m, 832m, 790s, 720s, 694m, and 670s. MS (APCI) *m*/*z*: [M + H]^+^ 398.3, 400.3. mp (°C): 163. Elemental analysis calculated for C_10_H_8_NS_5_BrO (%): C: 30.15; H: 2.02; N: 3.52; and S: 40.24. Found: C: 30.5; H: 1.95; N: 3.38; and S: 39.63.

6-Ethoxy-9-iodo-[1,2,3,4,5]pentathiepino[6,7-*a*]indolizine (**3e**): yield (under air): 70%, yield (under N_2_): 70%, yellow crystalline powder, R_f_ = 0.3 (Hex/EtOAc = 95/5, *v*/*v*). ^1^H NMR (CD_2_Cl_2_, 300 MHz): δ 7.96 (dd, 1H, *J* = 1.5 and 0.9 Hz), 7.20 (dd, 1H, *J* = 9.4 and 1.0 Hz), 6.94 (dd, 1H, *J* = 9.4 and 1.5 Hz), 4.41 (qd, 2H, *J* = 7.1 and 2.5 Hz), and 1.38 (s, 3H, *J* = 7.1). ^13^C{^1^H} NMR (CD_2_Cl_2_, 75.5 MHz): δ 128.7, 126.0, 119.1, 113.3, 110.7, 75.7, 72.5, and 15.3. UV-vis (THF) λ_max_ = 406.5 nm; IR (KBr disk, cm^−1^): ν = 1610w, 1531s, 1503w, 1486w, 1434w, 1386w, 1350m, 1325s, 1269s, 1227s, 1137m, 1039w, 1008s, 980w, 880w, 835w, 792s, 690w, and 660m. MS (APCI) *m*/*z*: [M + H]^+^ 446.3. mp (°C): 158.

6-Ethoxy-9-nitro-[1,2,3,4,5]pentathiepino[6,7-*a*]indolizine (**3f**): yield (under air): 73%, yield (under N_2_): 0%, red crystalline powder, R_f_ = 0.1 (Hex/EtOAc = 95/5, *v*/*v*). ^1^H NMR (CD_2_Cl_2_, 300 MHz): δ 8.80 (dd, 1H, *J* = 1.8 and 1.0 Hz), 7.60-7.33 (m, 2H), 4.54 (qd, 2H, *J* = 7.0 and 1.7 Hz), and 1.43 (t, 3H, *J* = 7.1 Hz). ^13^C{^1^H} NMR (CD_2_Cl_2_, 75.5 MHz): δ 142.8, 138.0, 129.6, 122.8, 118.9, 115.9, 113.7, 73.2, and 15.7. UV-vis (THF) λ_max_ = 378.5 nm; IR (KBr disk, cm^−1^): ν = 3094w, 2964w, 1625m, 1540s, 1469s, 1423m, 1379w, 1322s, 1256w, 1218w, 1170m, 1122w, 1065w, 920w, 894w, 826m, 739m, 740m, and 689w. MS (APCI) *m*/*z*: [M + H]^+^ 365.3. Elemental analysis calculated for C_10_H_8_N_2_S_5_O (%): C: 32.95; H: 2.21; N: 7.69; and S: 43.98. Found: C: 30.59; H: 1.77; N: 6.50; and S: 39.6. mp (°C): 140.

6-Ethoxy-9-methyl-[1,2,3,4,5]pentathiepino[6,7-*a*]indolizine (**3g**): yield (under air): 21%, yield (under N_2_): 32%, yellow crystalline powder, R_f_ = 0.3 (Hex/EtOAc = 95/5, *v*/*v*). ^1^H NMR (CD_2_Cl_2_, 300 MHz): δ 7.44 (h, 1H, *J* = 1.3 Hz), 7.36-7.25 (m, 1H), 6.63 (ddd, 1H, *J* = 9.3, 1.5, and 0.4 Hz), 4.35 (qd, 2H, *J* = 7.0 and 3.1 Hz), 2.14 (d, 3H, *J* = 1.3 Hz), and 1.36 (t, 3H, *J* = 7.0 Hz). ^13^C{^1^H} NMR (CD_2_Cl_2_, 75.5 MHz): δ 140.3, 129.3, 124.7, 122.2, 118.3, 117.4, 113.0, 108.5, 72.3, 18.1, and 15.4. UV-vis (THF) λ_max_ = 407.0 nm; IR (KBr disk, cm^−1^): ν = 2974w, 1536s, 1413w, 1352w, 1337m, 1284s, 1237w, 1211s, 1337s, 1015s, 915w, 865w, 837w, 669m, and 650w. MS (APCI) *m*/*z*: [M + H]^+^ 334.4. mp (°C): 120. Elemental analysis calculated for C_11_H_11_NS_5_O (%): C: 39.61; H: 3.32; N: 4.20; and S: 48.06. Found: C: 40.42; H: 3.36; N: 4.14; and S: 47.65.

## 4. Conclusions

The syntheses of 1,2,3,4,5-pentathiepines (indolizine derived and ethoxy substituted) via the molybdenum-mediated route were explored under inert gas conditions and in air. Regularly, both varieties are successful, albeit with most often better yields under inert gas. The exception is the derivative with a nitro substituent in the position C-9. For this, only the preparation in air succeeded, while the one under inert gas gave zero yield. It is hypothesized that here a distinct reaction mechanism is effective, although as of yet, this could not be elucidated. The molecular structures of the seven 1,2,3,4,5-pentathiepino[6,7-*a*]indolizines with an ethoxy substitution adjacent to the pentathiepine moiety of this study (as well as those already published) are quite notably conserved regardless of the varied substituent employed here in the position C-9. Their packing patterns do vary though, which is at least to some extent based on intermolecular interactions such as hydrogen bonding (most notable for the nitro-substituted derivative). The relative energies of the frontier orbitals are, according to their UV-vis spectra, essentially the same for all except for the nitro-substituted derivative, which shows a considerable apparent ‘blue shift’ in the UV-vis spectrum. This shift is, in fact, not a shift but a different transition compared to all other derivatives. As would be expected, the nitro substituent decreases the HOMO–LUMO gap energy, which would and does result in a red shift. The HOMO–LUMO transition for the nitro is of so low extinction that it is not observable, but the HOMO–LUMO+1 transition (also red-shifted relative to the other pentathiepines) becomes observable with sufficient extinction and is responsible for the distinct red color of the nitro derivative. The electron-withdrawing effect of the nitro group is quite evident in the ^1^H-NMR shifts of the aromatic protons, but it does not reach beyond the aromatic system as the remaining protons are essentially unaffected. The nitro substituent, with its notable influence on the electronic structure of the pentathiepine it is attached to, can be confirmed as a suitable group to tune the compound’s properties quite substantially. For the other substituents, this is not really the case, at least not in the position that was chosen for the variation. In order to exert a more prominent influence on reactivates and/or stabilities, more complex substituents and/or distinct positions will have to be explored in the future. The observed general stability of these derivatives, on the other hand, very strongly encourages further exploring them for biological/pharmaceutical applications.

## Data Availability

The crystallographic data were deposited with the CCDC and are available by download using the deposition numbers (see Section 3.1.2). Spectra, if not part of the manuscript, are provided in the Supplementary Materials. Original data are available from the authors upon request.

## Supplementary Materials

The following supporting information can be downloaded at https://www.mdpi.com/article/10.3390/molecules29010216/s1: Figure S1–S15: ^1^H-, ^13^C-, and ^19^F-NMR spectra of **3a**, **3b**, **3c**, **3d**, **3e**, **3f**, and **3g**; Figure S16–S30: ^1^H-, ^13^C-, and ^19^F-NMR spectra of **2a**, **2b**, **2c**, **2d**, **2e**, **2f**, and **2g**; Figure S31–S37: UV-vis spectra of **3a**, **3b**, **3c**, **3d**, **3e**, **3f**, and **3g**; Figure S38–S44: IR spectra of **3a**, **3b**, **3c**, **3d**, **3e**, **3f**, and **3g**; Figure S45–S51: APCI mass spectra of **3a**, **3b**, **3c**, **3d**, **3e**, **3f**, and **3g**; Figure S52–S58: APCI mass spectra of **2a**, **2b**, **2c**, **2d**, **2e**, **2f**, and **2g**; Tables S1–S7: quantum chemical calculations for **3a** and **3f**; Tables S8–S14: crystal and structure refinement data for **3a**, **3b**, **3c**, **3d**, **3e**, **3f**, **and 3g**; synthesis of **1b**.
