# Supplementary material for: Synthesis of Seven Indolizine-Derived Pentathiepines: Strong Electronic Structure Response to Nitro Substitution in Position C-9
Source: Molecules. 2023 Dec 30;29(1):216. doi: 10.3390/molecules29010216 (PMC10780577; doi:10.3390/molecules29010216)
Supplement: Supplementary file 1 [file molecules-29-00216-s001.zip › molecules-2775047-supplementary.pdf]

Supporting Information

Synthesis of seven indolizine derived pentathiepines – strong electronic structure response to nitro substitution in position C-9

Roberto Tallarita [1], Lukas M. Jacobsen [1], Benedict J. Elvers[1], Stefan Richter[1], Siva S. M. Bandaru[1], Jevy V. Correia[1] and Carola Schulzke[1,*]

[1] Bioinorganic Chemistry, Institute of Biochemistry, University of Greifswald, Felix-Hausdorff-Str. 4, 17489 Greifswald, Germany; roberto.tallarita@uni-greifswald.de; lukasmanuel.jacobsen@stud.uni-greifswald.de; benedict.elvers@uni-greifswald.de; stefan.richter@uni-greifswald.de; siva.bandaru@uni-greifswald.de; correiaj@uni-greifswald.de; carola.schulzke@uni-greifswald.de
* Correspondence: carola.schulzke@uni-greifswald.de; Tel.: +49 3034 420 4321

Table of content

Figg. S1 – S15. ^{1}H, ^{13}C and ^{19}F NMR spectra of **3a, 3b**, **3c**, **3d**, **3e**, **3f**, **3g**.
Figg. S16 – S30 ^{1}H, ^{13}C and ^{19}F NMR spectra of **2a, 2b**, **2c**, **2d**, **2e**, **2f**, **2g**.
Figg. S31 – S37. UV-Vis spectra of **3a, 3b**, **3c**, **3d**, **3e**, **3f**, **3g**.
Figg. S38 – S44. IR spectra of **3a**, **3b**, **3c**, **3d**, **3e**, **3f**, **3g**.
Figg. S45 – S51. APCI Mass spectra of **3a**, **3b**, **3c**, **3d**, **3e**, **3f**, **3g**.
Figg. S52 – S58. APCI Mass spectra of **2a**, **2b**, **2c**, **2d**, **2e**, **2f**, **2g**.
Tables S1 – S7. Quantum Chemical Calculations for **3a** and **3f**.
Tables S8 – S14. Crystal data and structure refinement for **3a, 3b**, **3c**, **3d**, **3e**, **3f**, **3g**.
Synthesis of **1b**

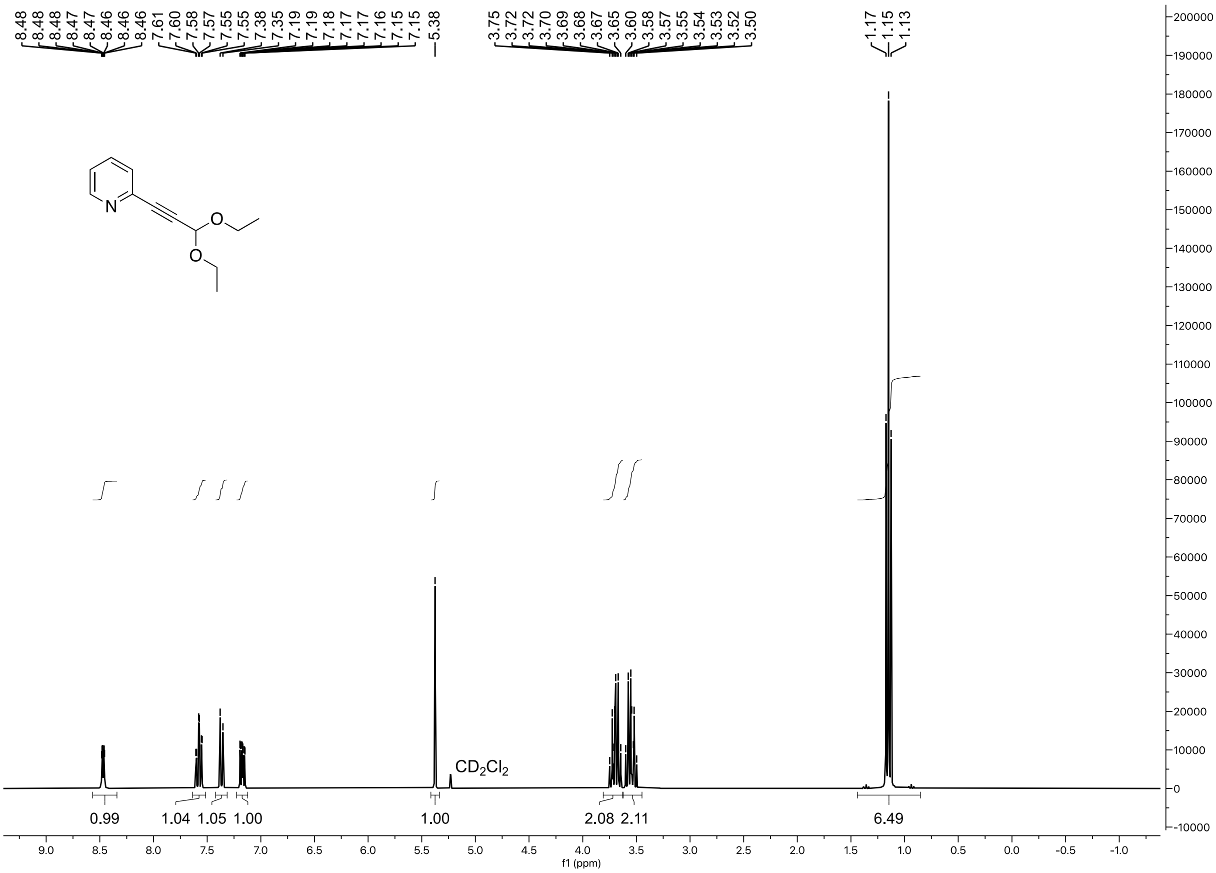

Figure S1. ^{1}H NMR spectrum of 2-(3,3-diethoxyprop-1-yn-1-yl)pyridine (**2a**).

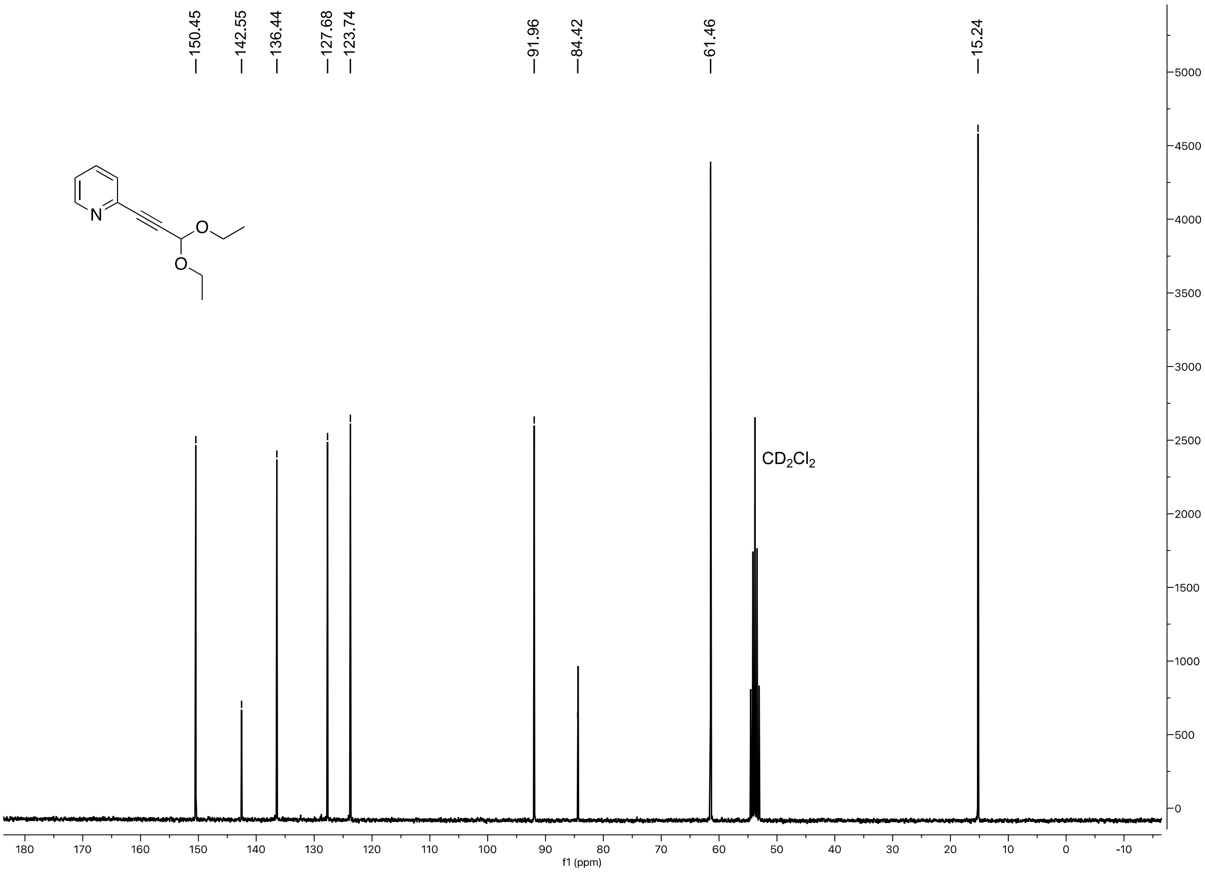

Figure S2. ^{13}C NMR spectrum of 2-(3,3-diethoxyprop-1-yn-1-yl)pyridine (**2a**).

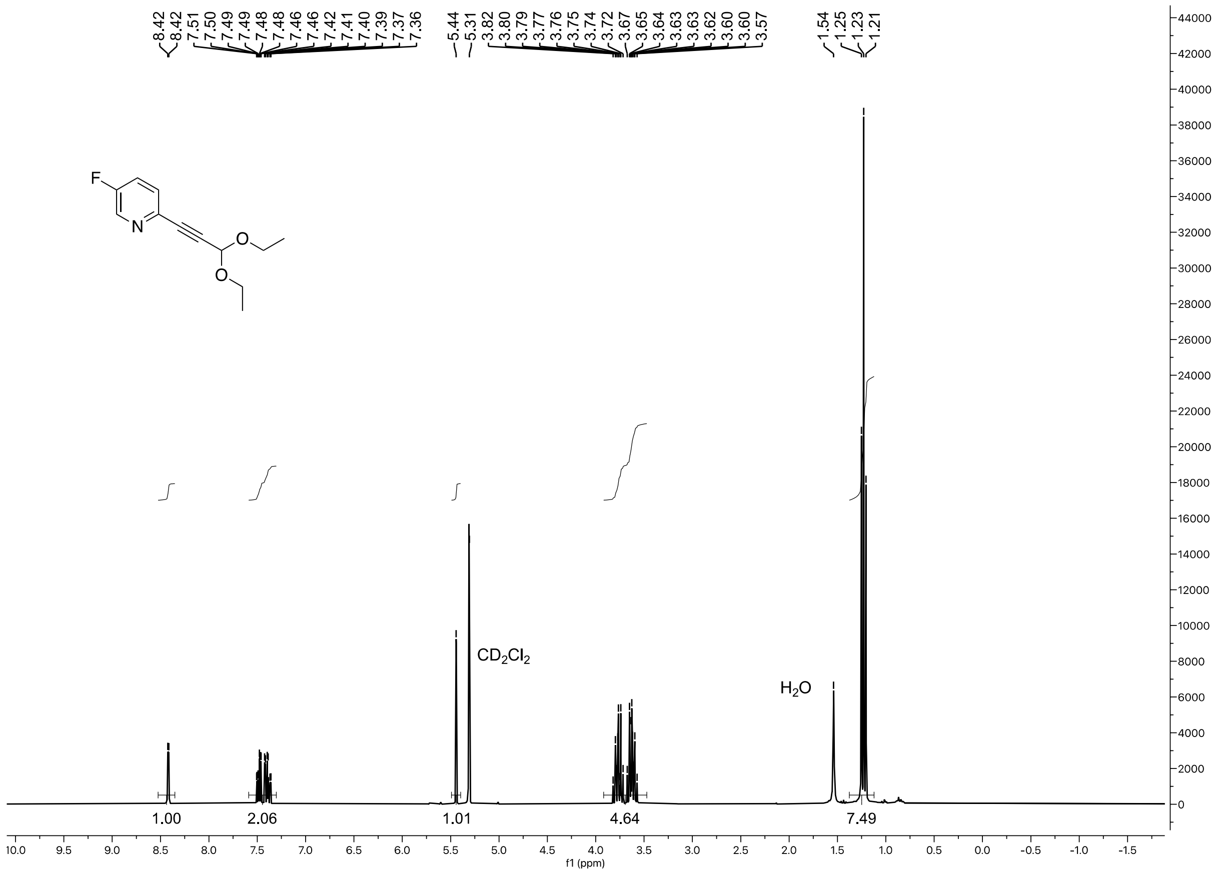

Figure S3. ^{1}H NMR spectrum of 5-fluoro-2-(3,3-diethoxyprop-1-yn-1-yl)pyridine (**2b**).

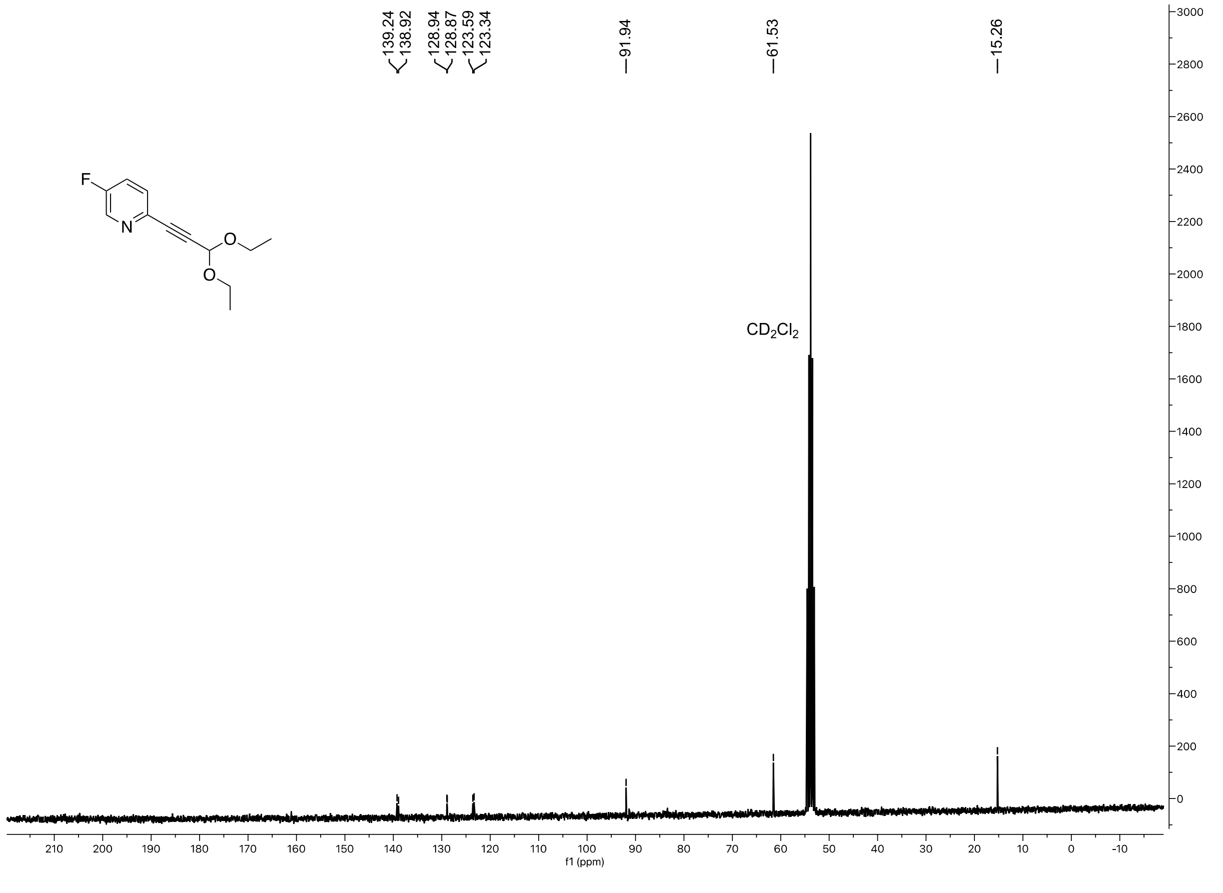

Figure S4. ^{13}C NMR spectrum of 5-fluoro-2-(3,3-diethoxyprop-1-yn-1-yl)pyridine (**2b**).

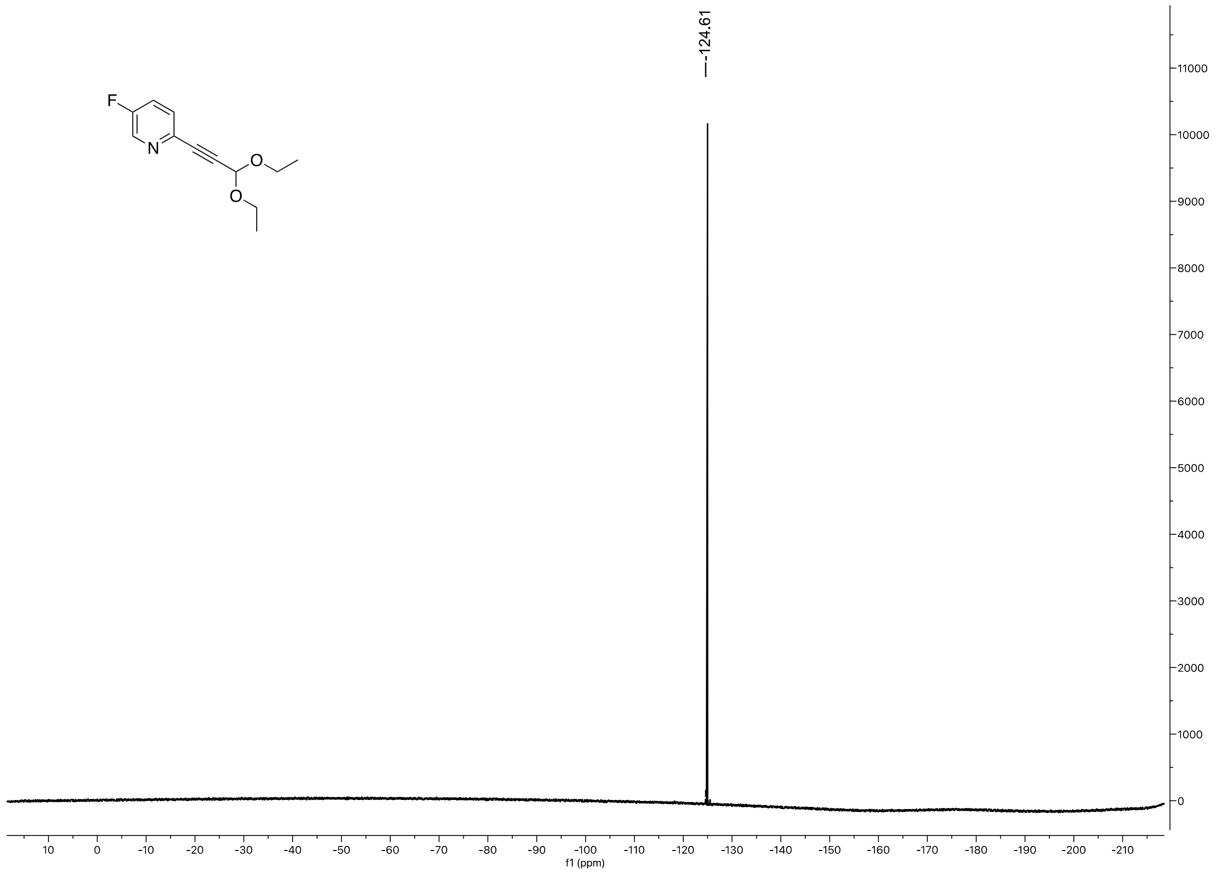

Figure S5. ^{19}F NMR spectrum of 5-fluoro-2-(3,3-diethoxyprop-1-yn-1-yl)pyridine (**2b**).

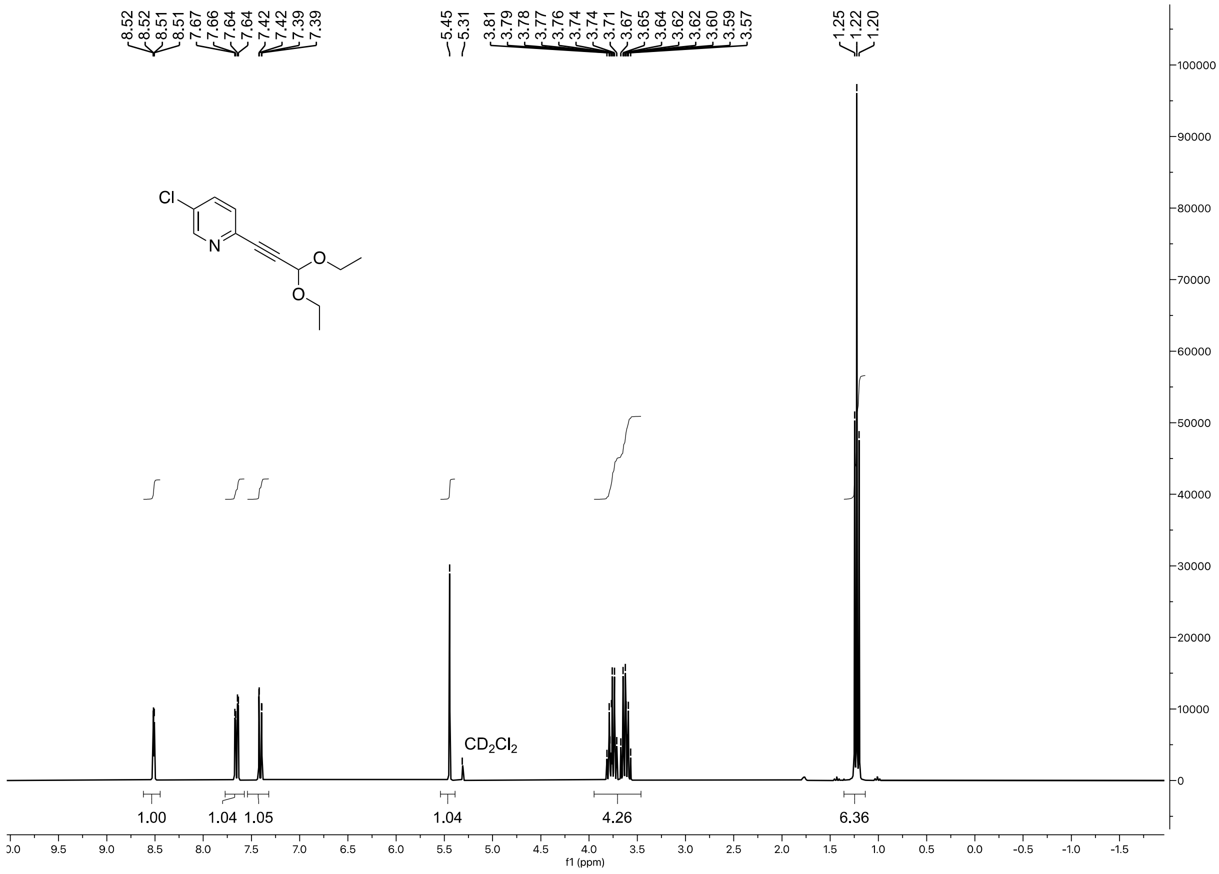

Figure S6. ^{1}H NMR spectrum of 5-chloro-2-(3,3-diethoxyprop-1-yn-1-yl)pyridine (**2c**).

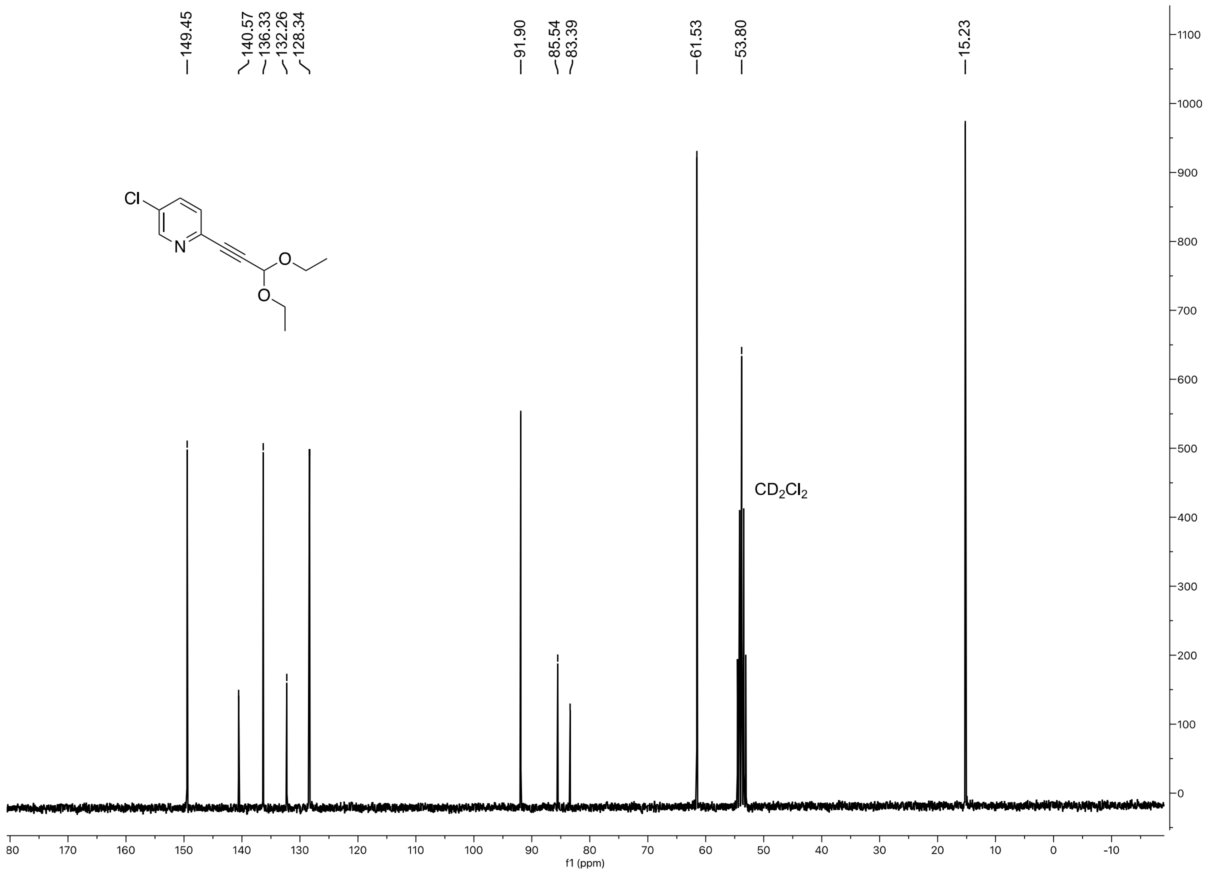

Figure S7. ^{13}C NMR spectrum of 5-chloro-2-(3,3-diethoxyprop-1-yn-1-yl)pyridine (**2c**).

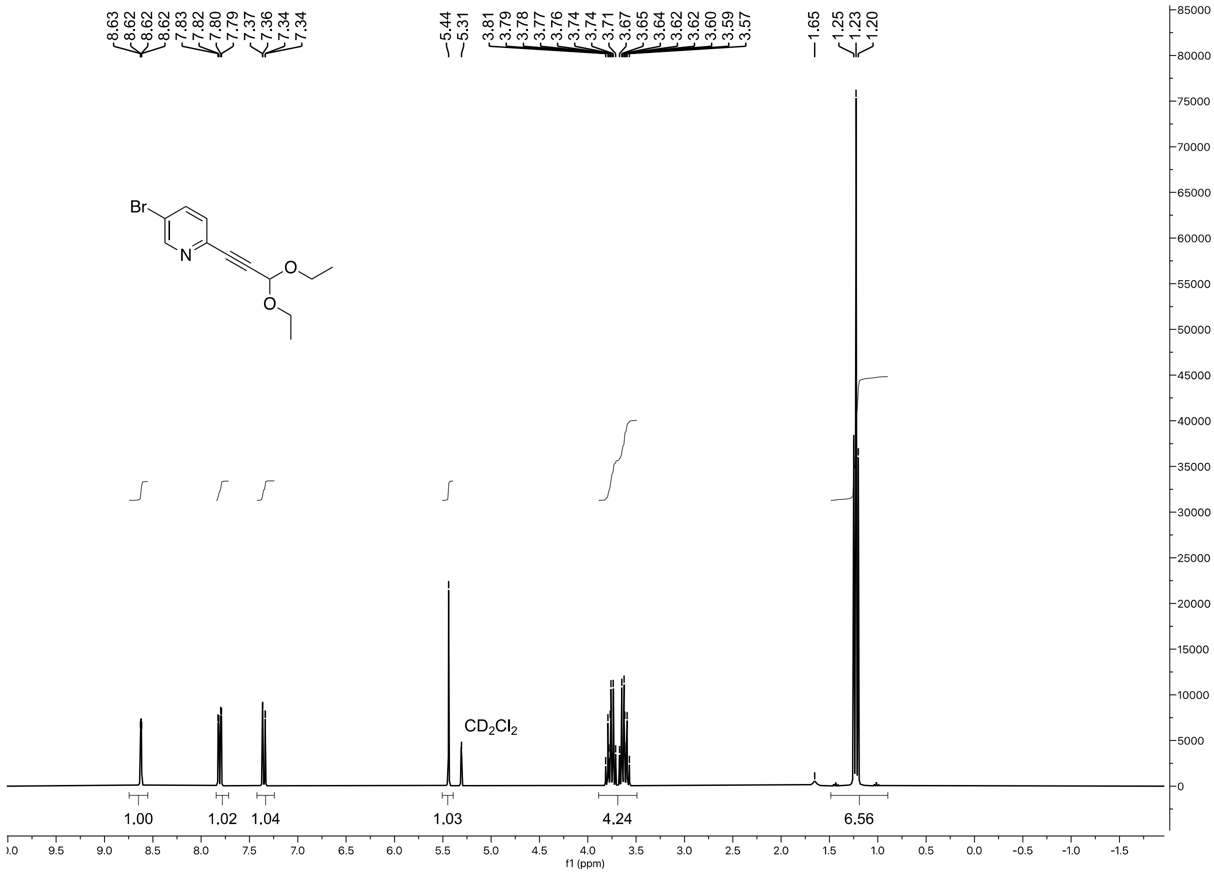

Figure S8. ^{1}H NMR spectrum of 5-bromo-2-(3,3-diethoxyprop-1-yn-1-yl)pyridine (**2d**).

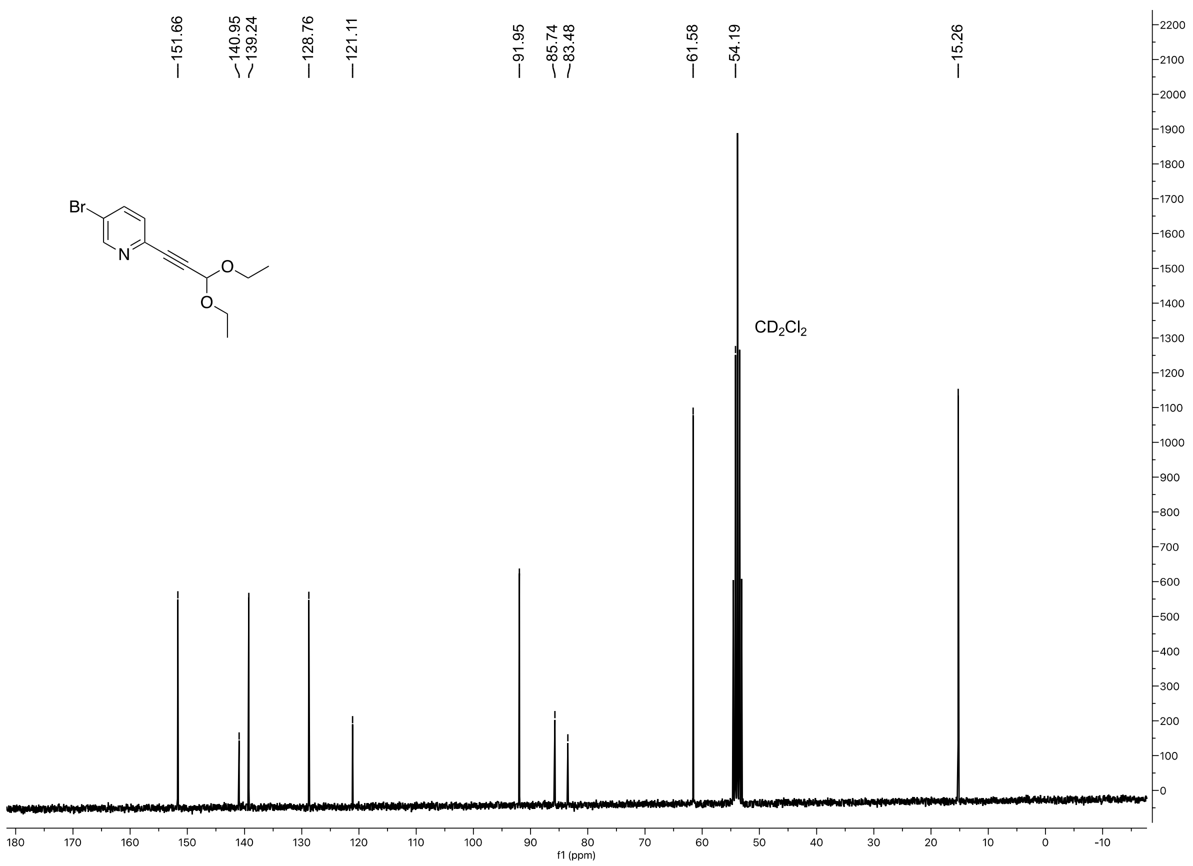

Figure S9. ^{13}C NMR spectrum of 5-bromo-2-(3,3-diethoxyprop-1-yn-1-yl)pyridine (**2d**).

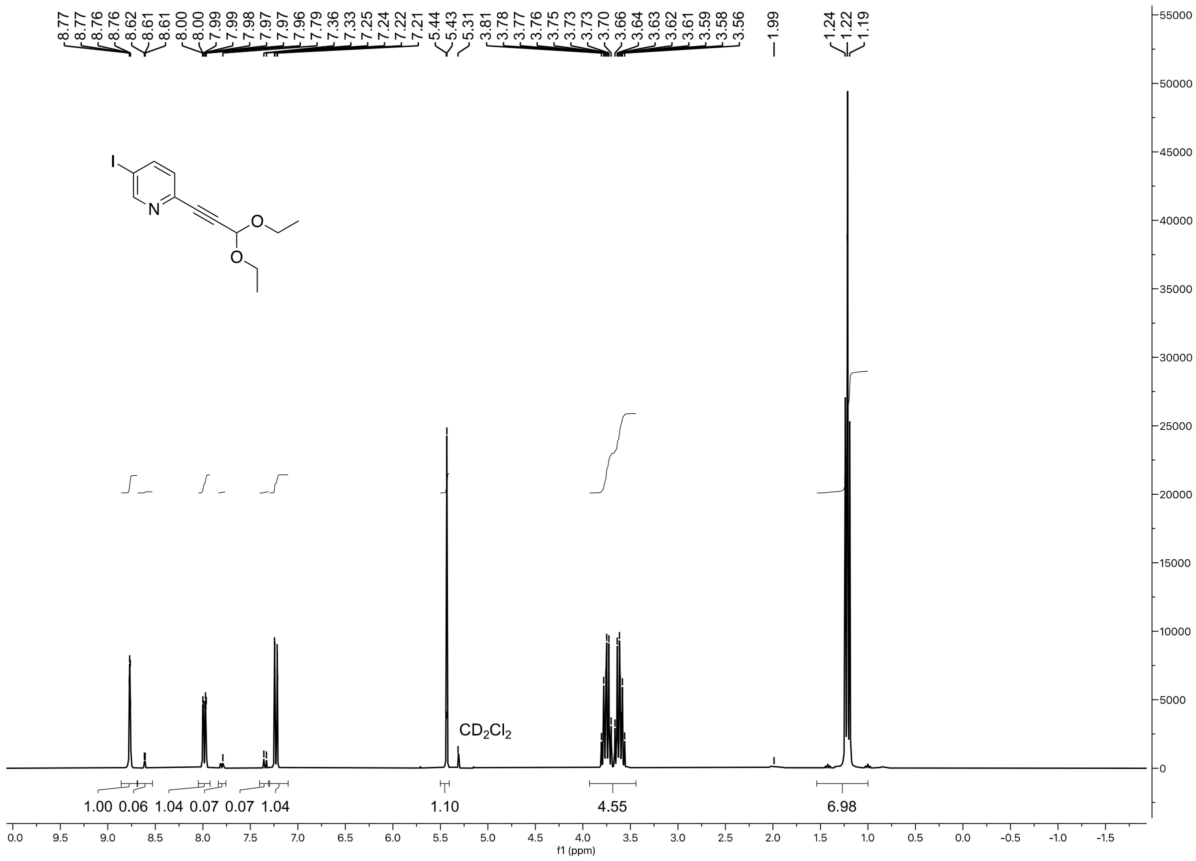

Figure S10. ^{1}H NMR spectrum of 5-iodo-2-(3,3-diethoxyprop-1-yn-1-yl)pyridine (**2e**).

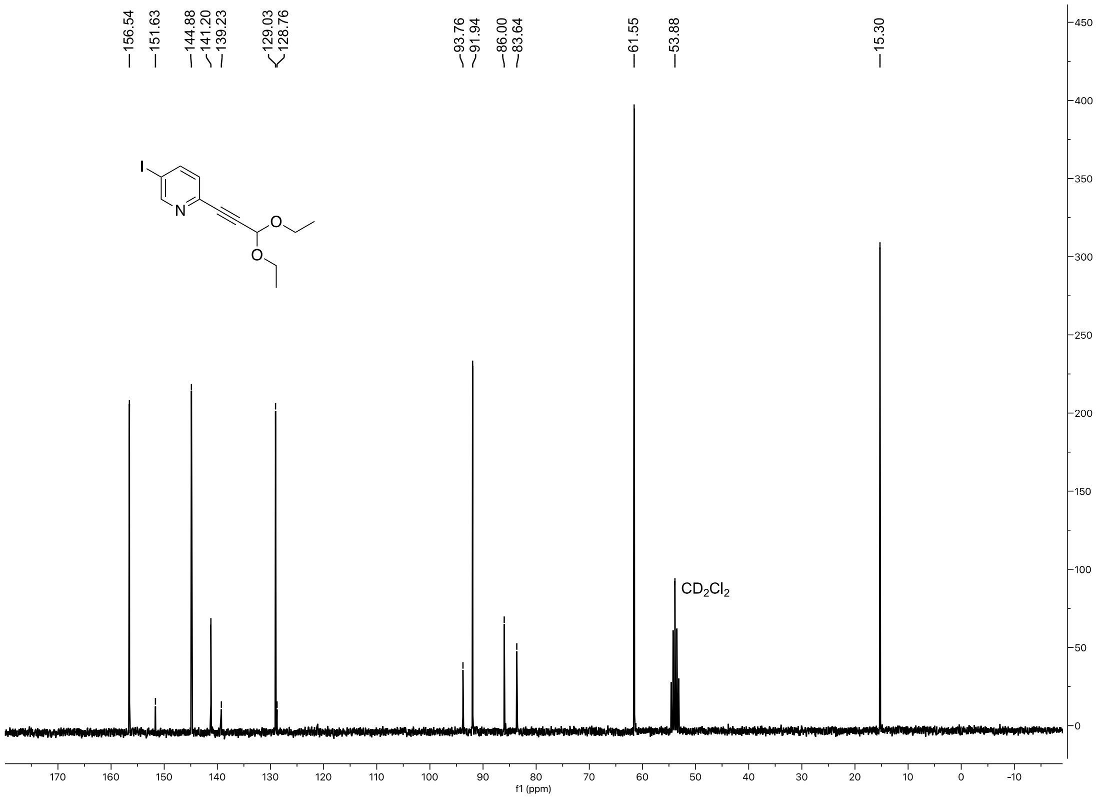

Figure S11. ^{13}C NMR spectrum of 5-iodo-2-(3,3-diethoxyprop-1-yn-1-yl)pyridine (**2e**)

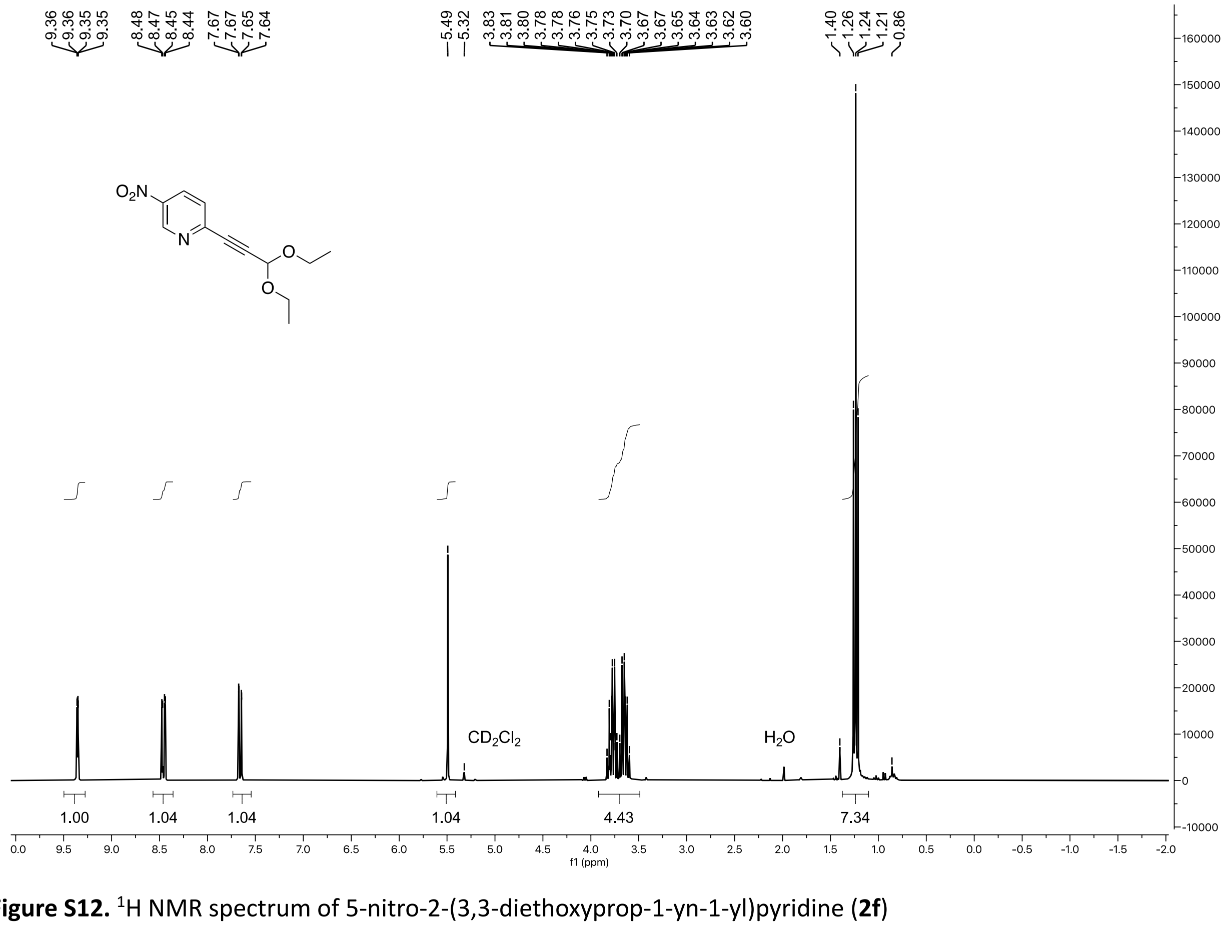

Figure S12. ^{1}H NMR spectrum of 5-nitro-2-(3,3-diethoxyprop-1-yn-1-yl)pyridine (**2f**)

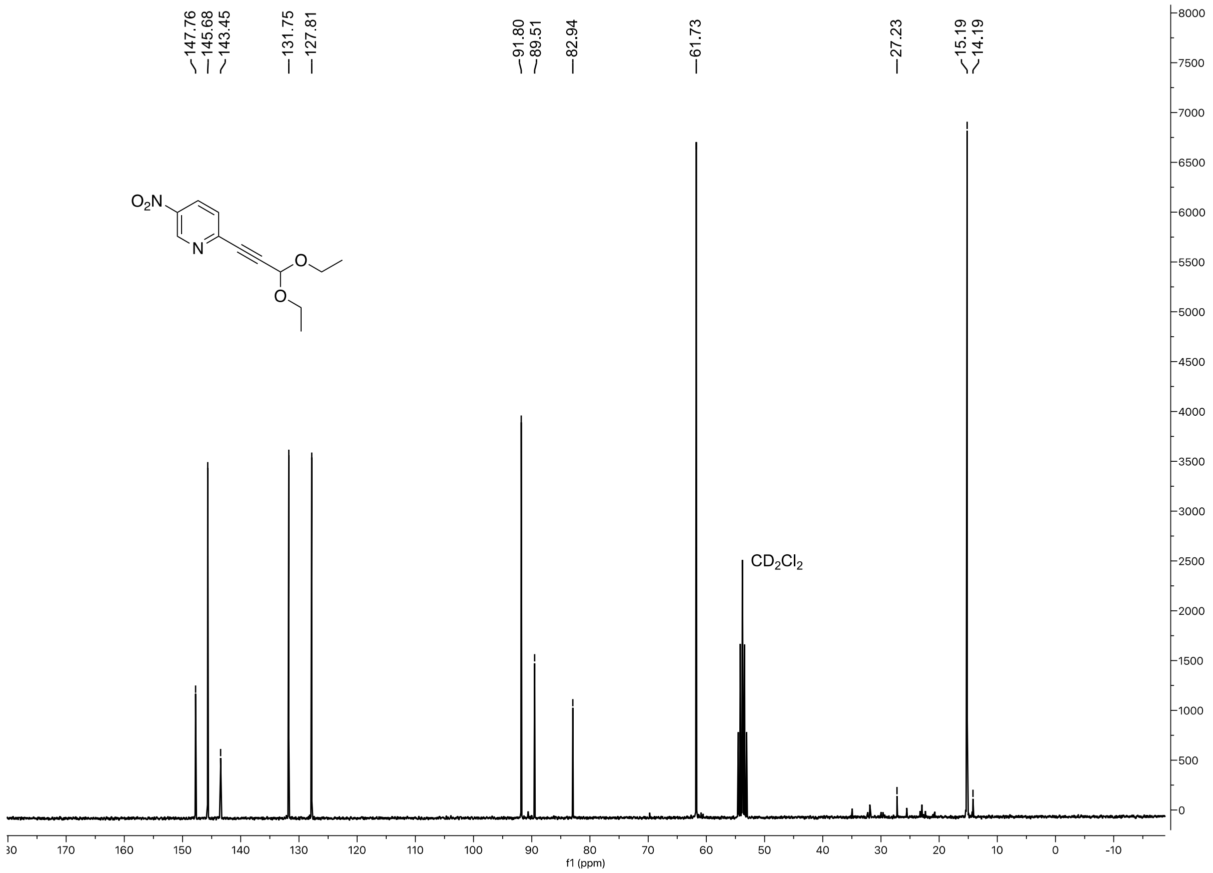

Figure S13. ^{13}C NMR spectrum of 5-nitro-2-(3,3-diethoxyprop-1-yn-1-yl)pyridine (**2f**).

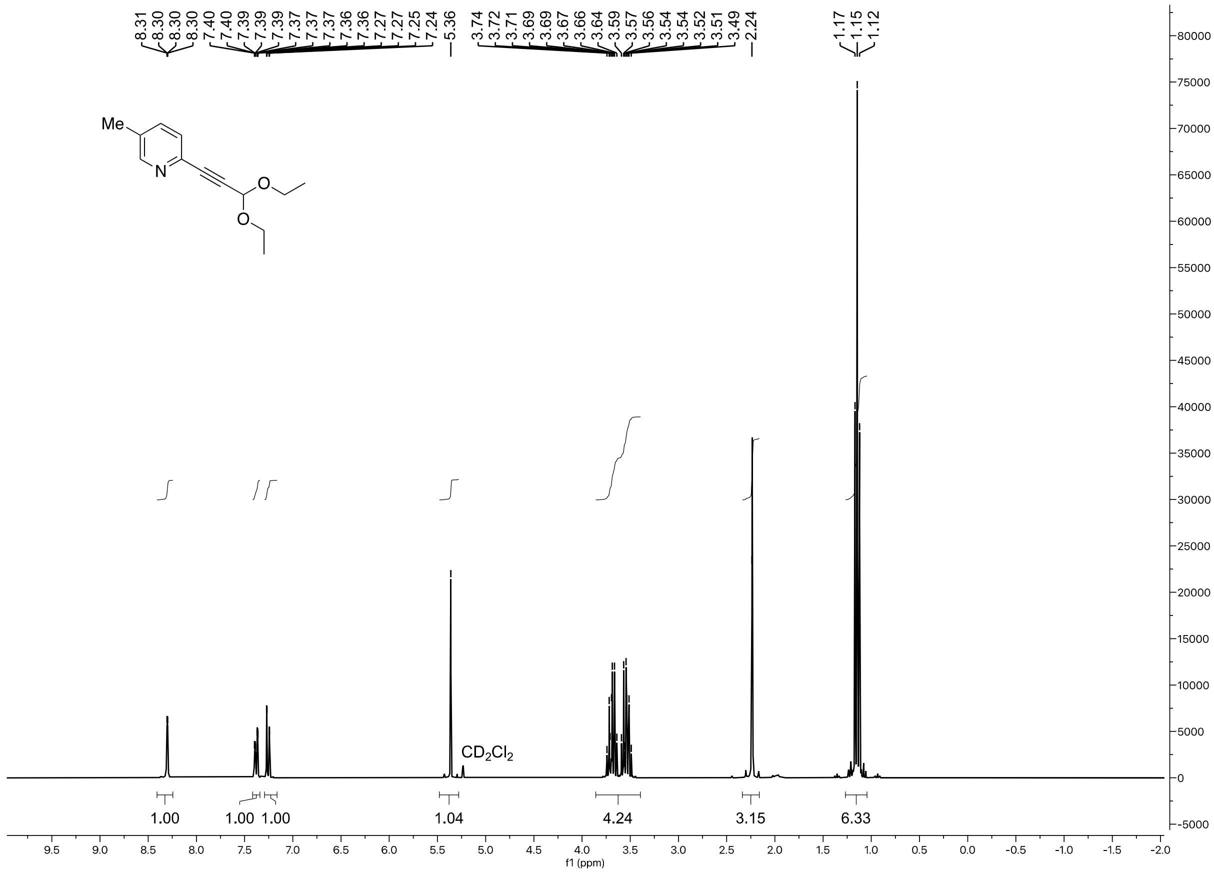

Figure S14. ^{1}H NMR spectrum of 5-methyl-2-(3,3-diethoxyprop-1-yn-1-yl)pyridine (**2g**).

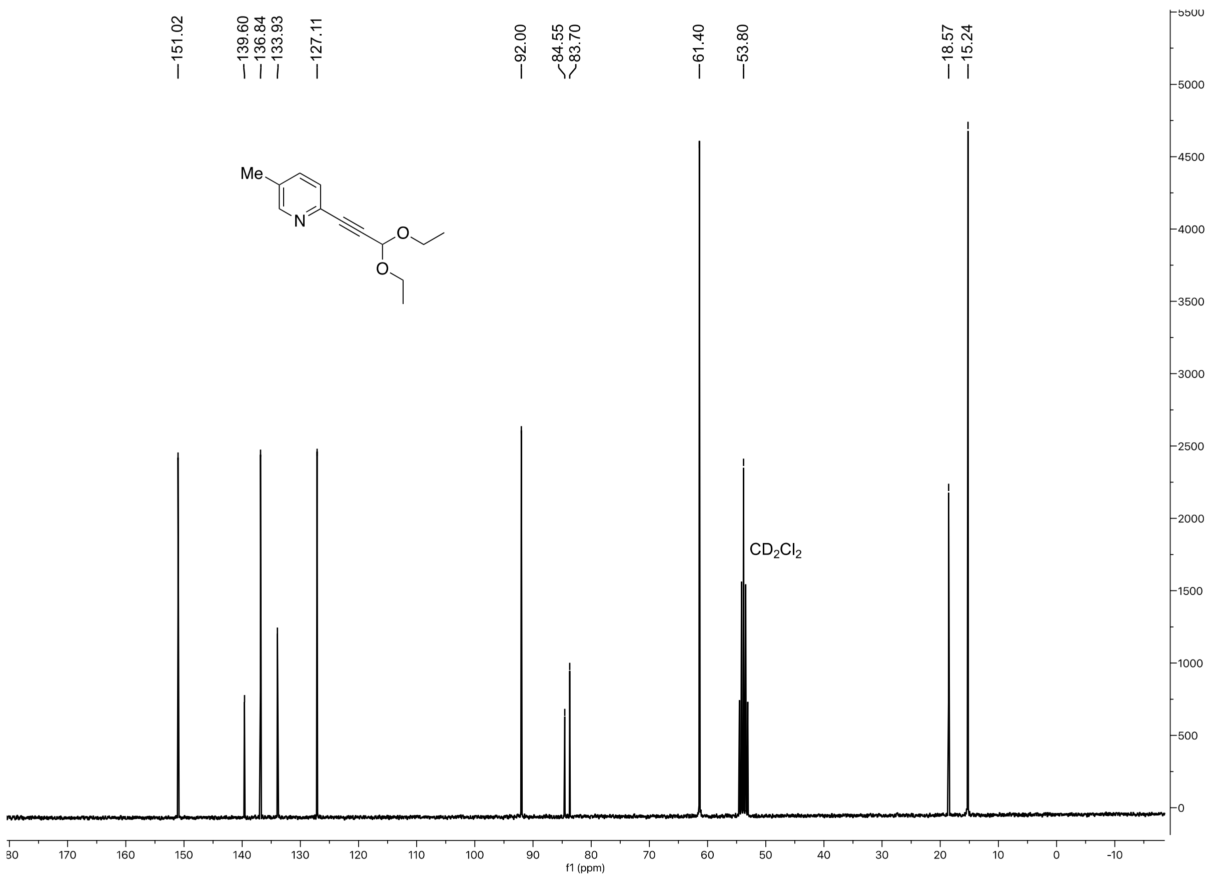

Figure S15. ^{13}C NMR spectrum of 5-methyl-2-(3,3-diethoxyprop-1-yn-1-yl)pyridine (**2g**).

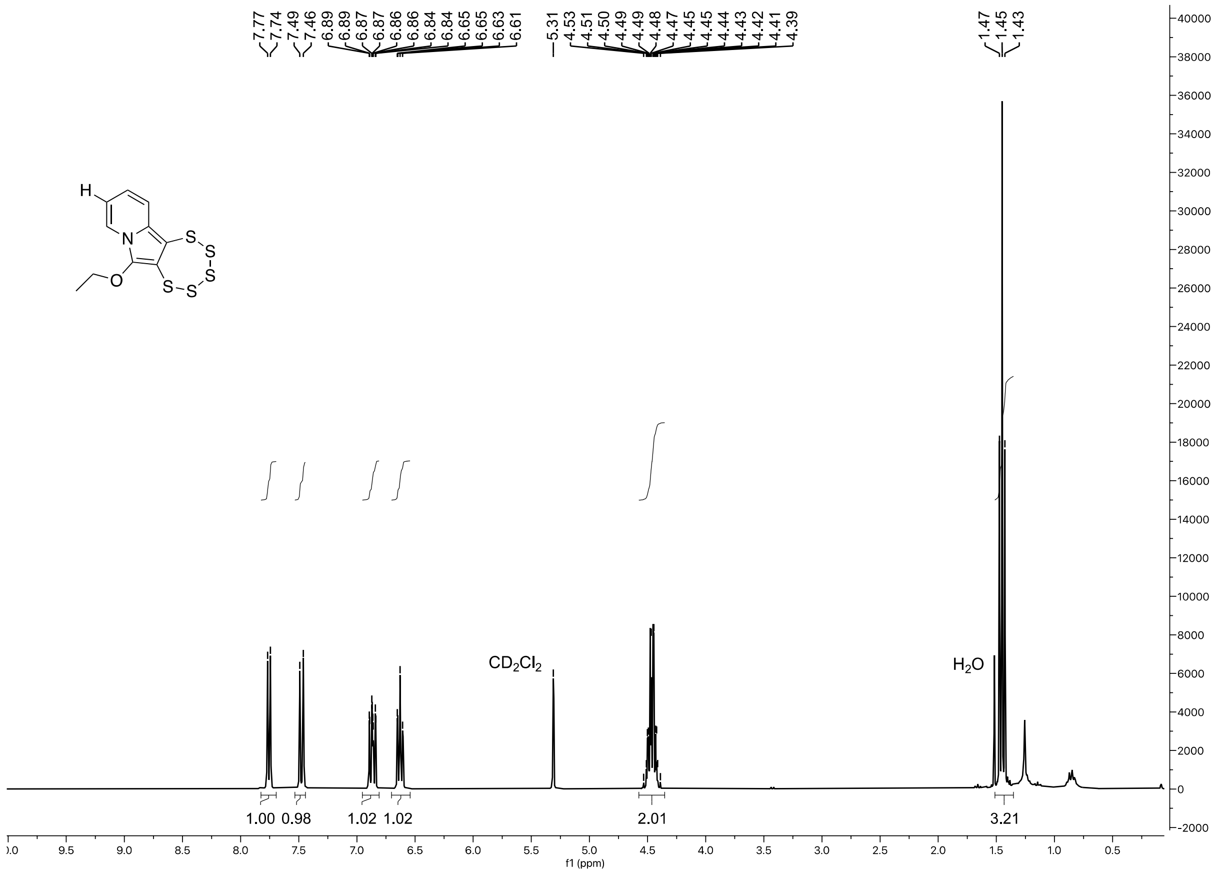

Figure S16. ^{1}H NMR spectrum of 6-ethoxy-[1,2,3,4,5]pentathiepino[6,7-a]indolizine (**3a**).

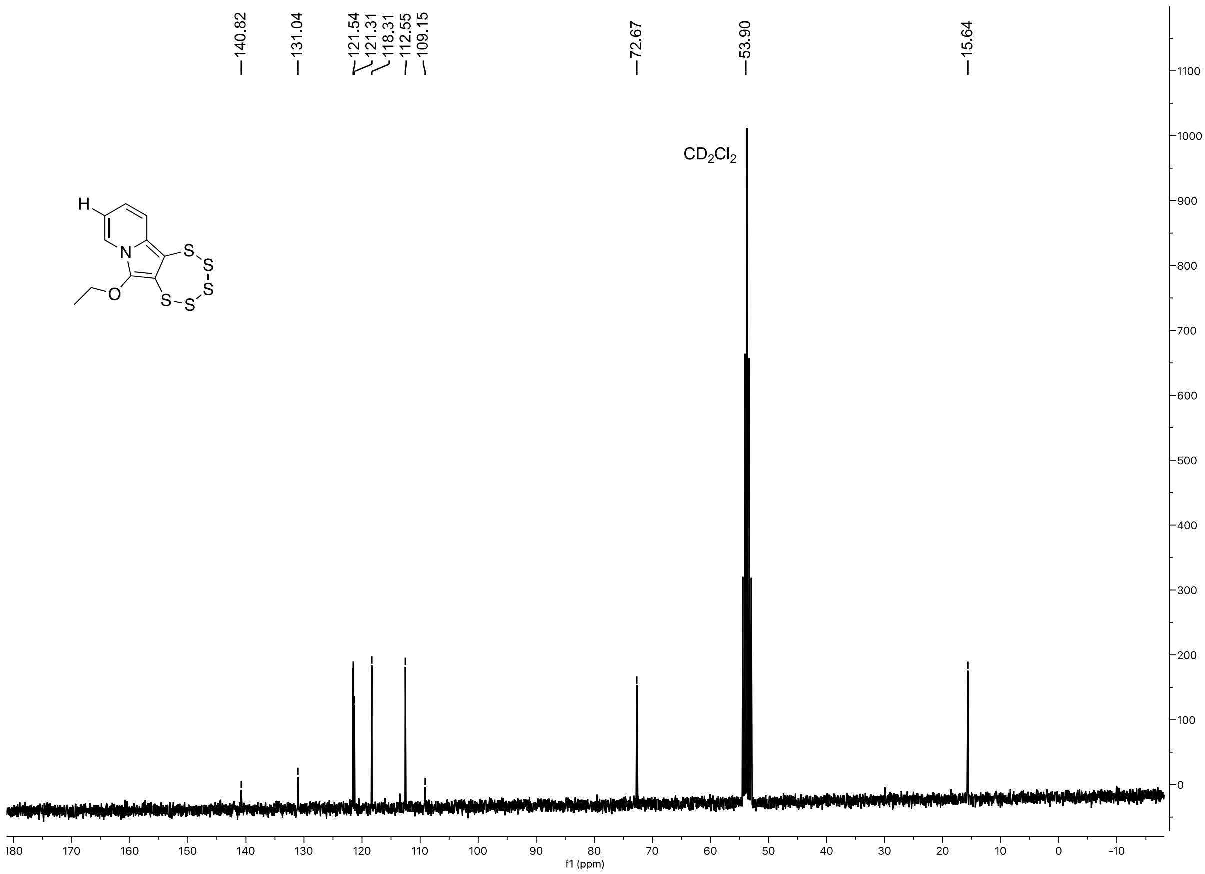

Figure S17. ^{13}C NMR spectrum of 6-ethoxy-[1,2,3,4,5]pentathiepino[6,7-a]indolizine (**3a**).

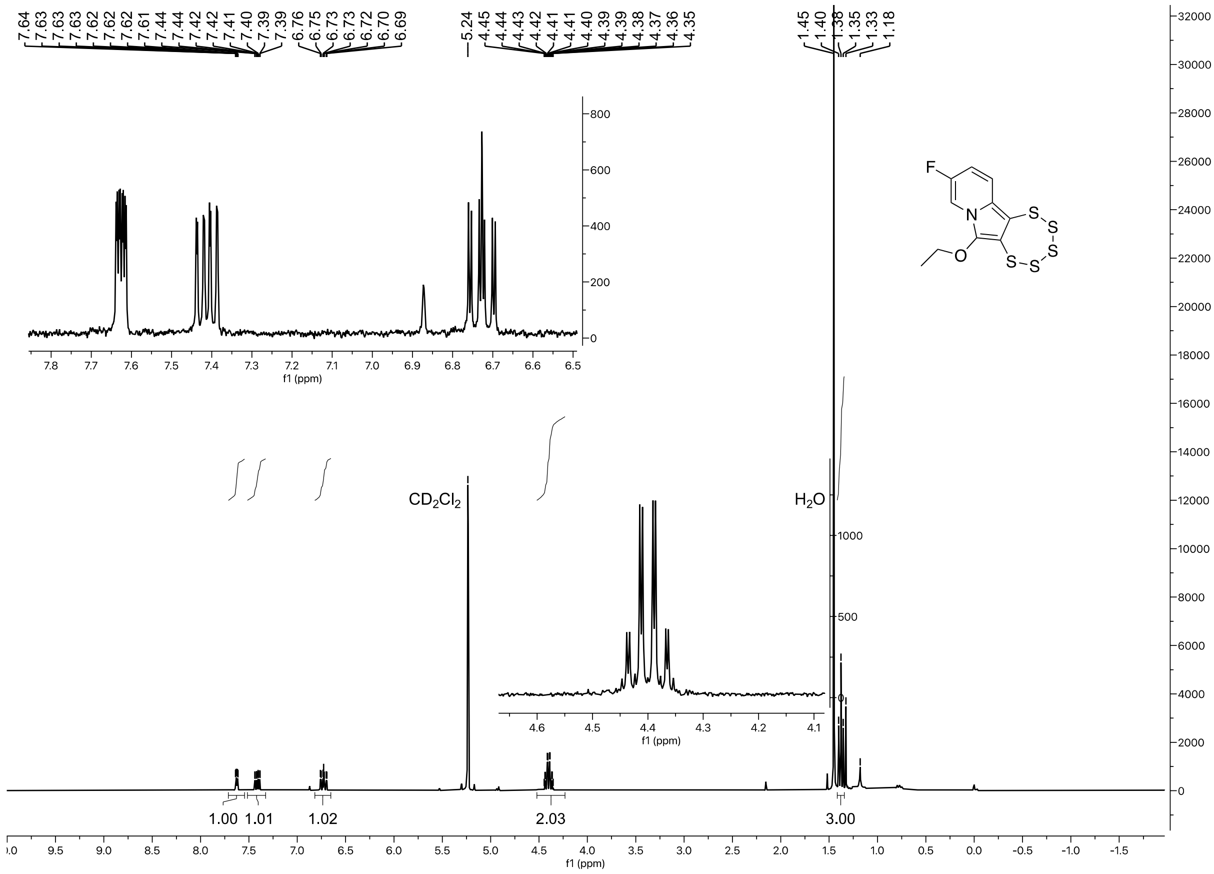

Figure S18. ^{1}H NMR spectrum of 6-ethoxy-9-fluoro-[1,2,3,4,5]pentathiepino[6,7-a]indolizine with zoom on signals (**3b**).

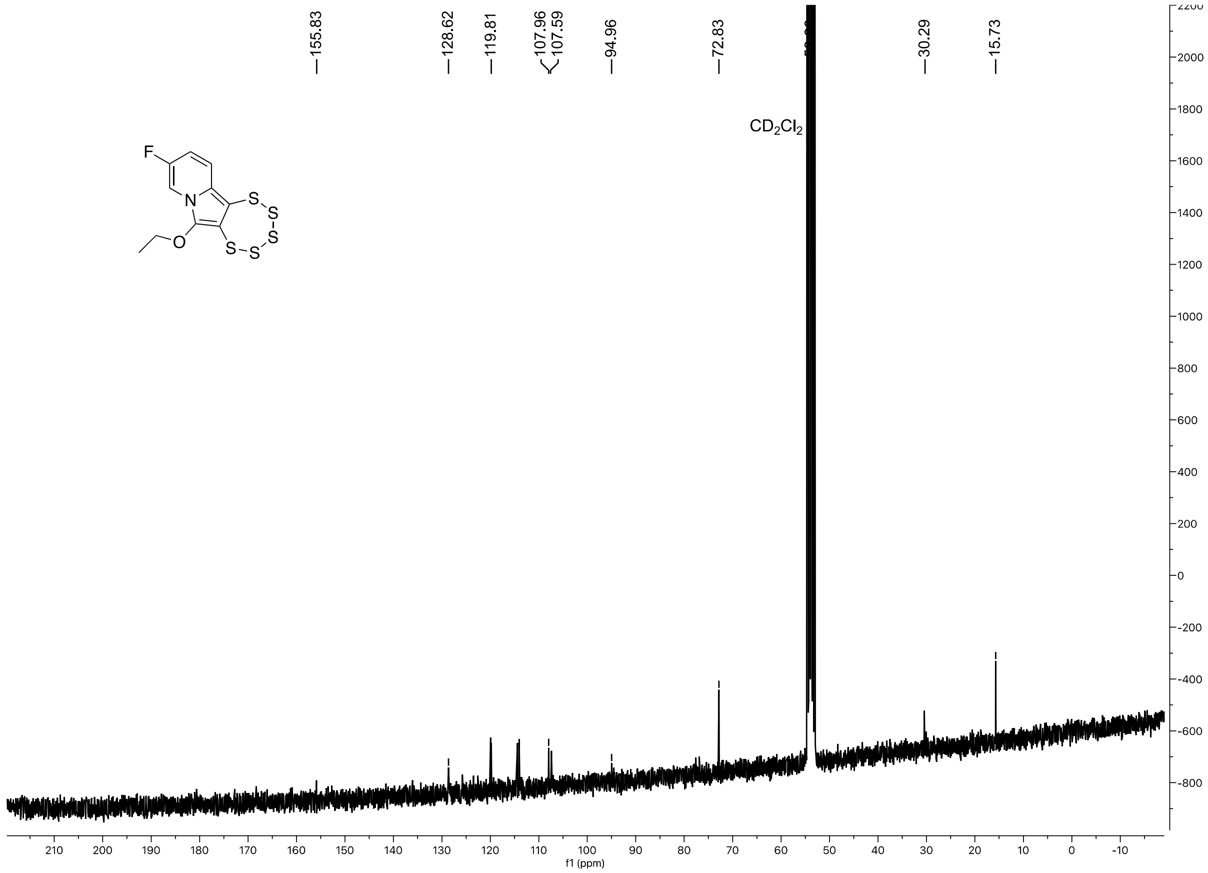

Figure S19. ^{13}C NMR spectrum of 6-ethoxy-9-fluoro-[1,2,3,4,5]pentathiepino[6,7-a]indolizine (**3b**). The baseline drift is due to the essential zooming for peak observation, consequence of a diluted sample, caused by the low reaction yield.

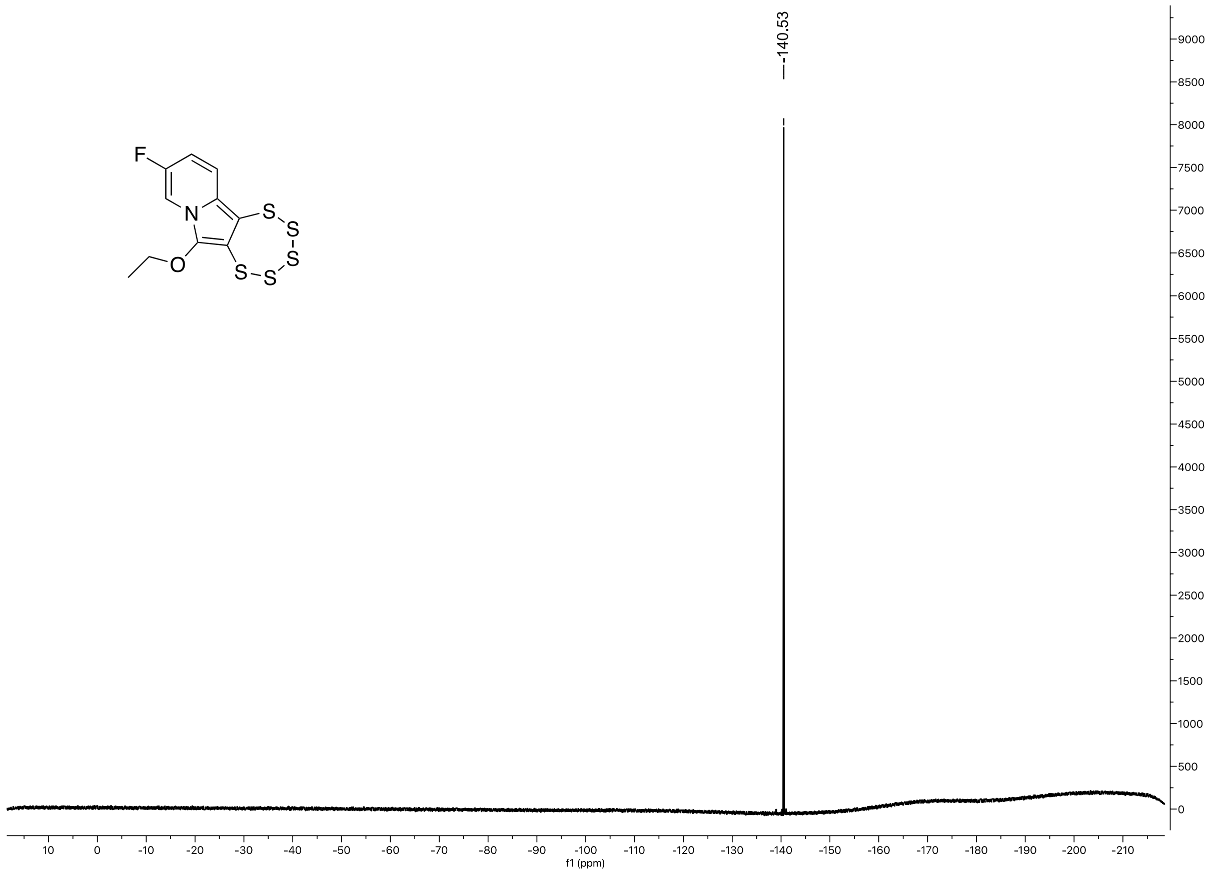

Figure S20. ^{19}F NMR spectrum of 6-ethoxy-9-fluoro-[1,2,3,4,5]pentathiepino[6,7-a]indolizine (**3b**).

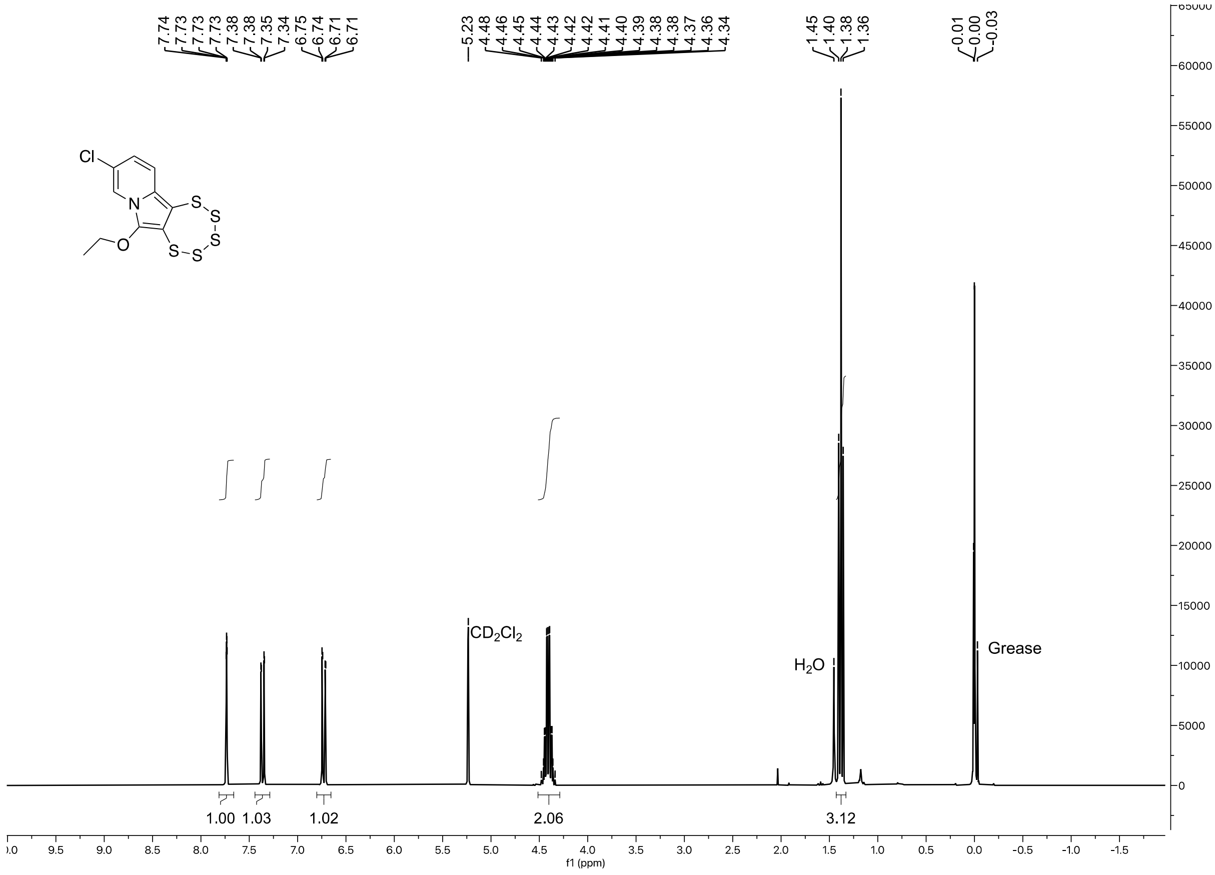

Figure S21. ^{1}H NMR spectrum 6-ethoxy-9-chloro-[1,2,3,4,5]pentathiepino[6,7-a]indolizine (**3c**).

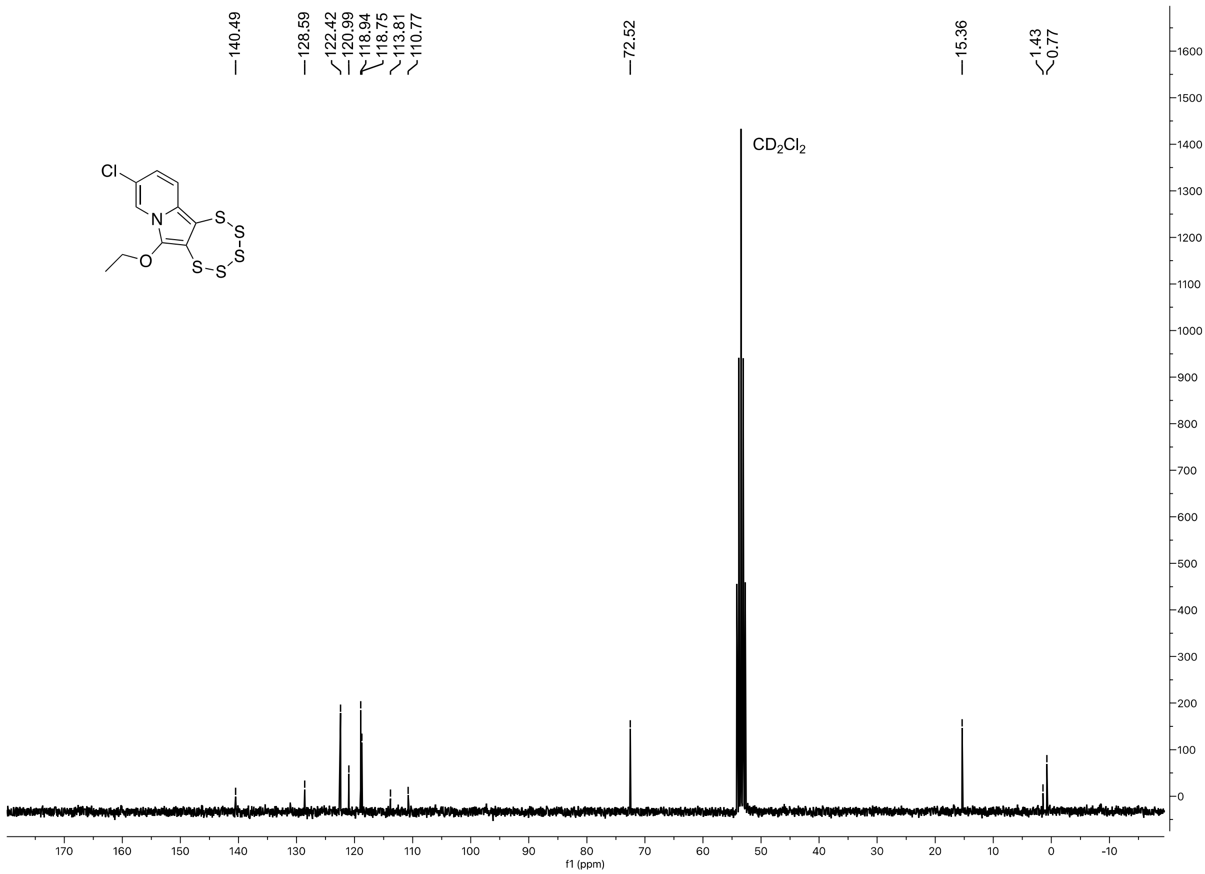

Figure S22. ^{13}C NMR spectrum 6-ethoxy-9-chloro-[1,2,3,4,5]pentathiepino[6,7-a]indolizine (**3c**).

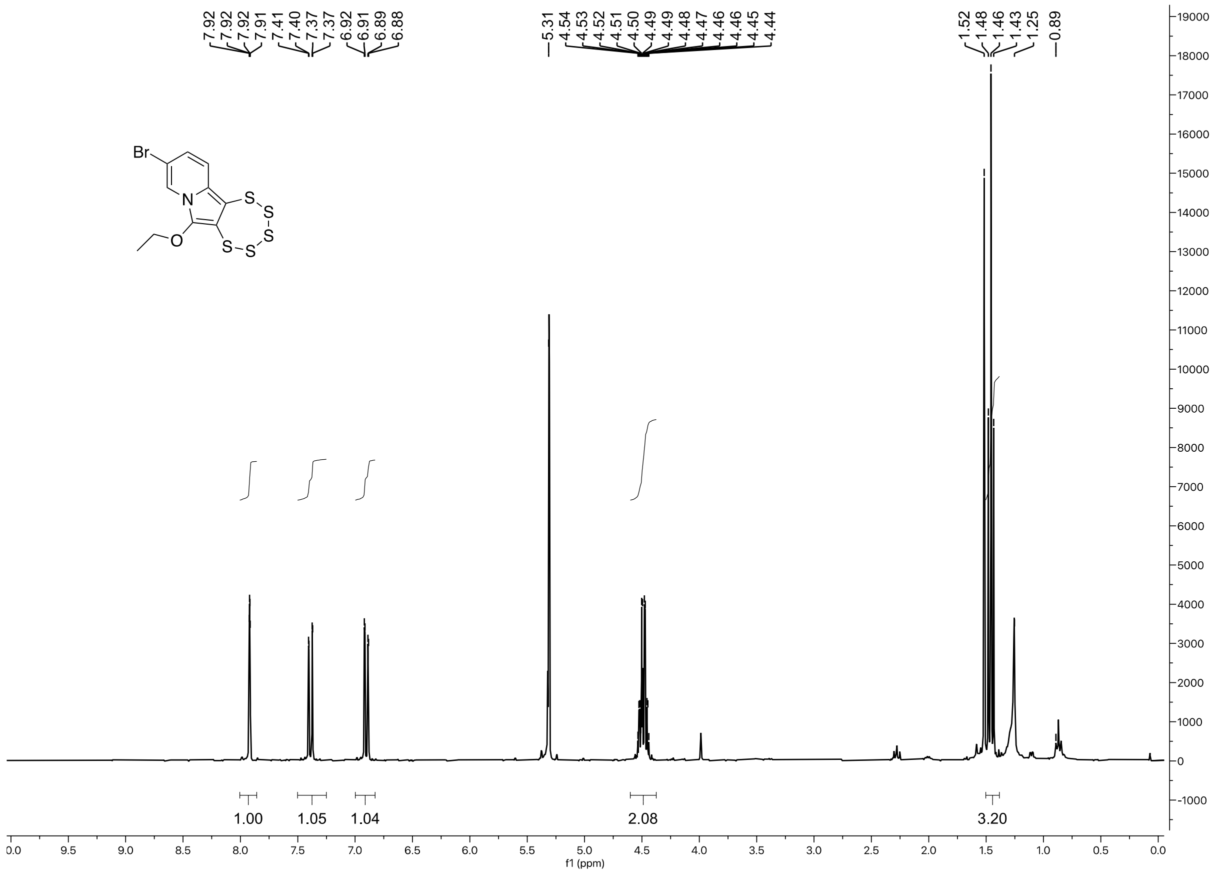

Figure S23. ^{1}H NMR spectrum of 6-ethoxy-9-bromo-[1,2,3,4,5]pentathiepino[6,7-a]indolizine (**3d**).

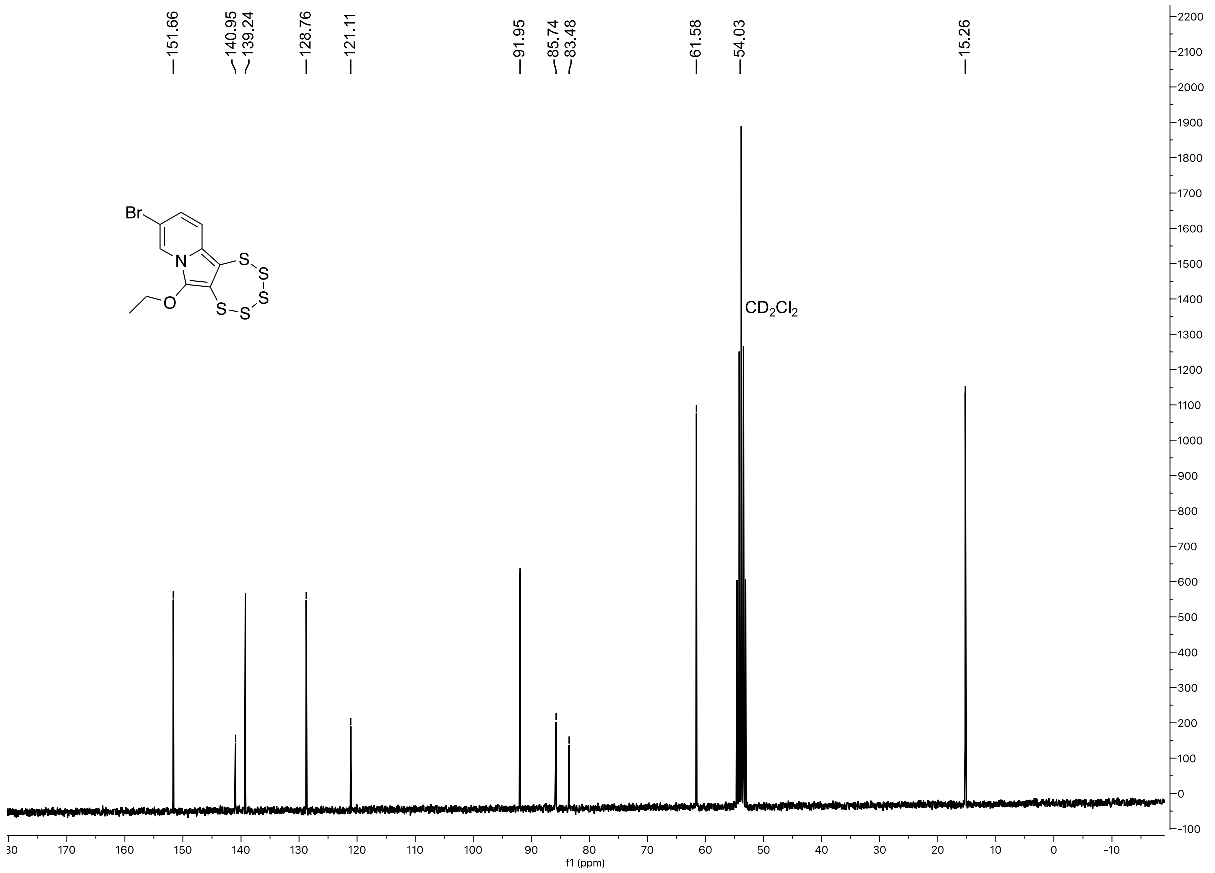

Figure S24. ^{13}C NMR spectrum of 6-ethoxy-9-bromo-[1,2,3,4,5]pentathiepino[6,7-a]indolizine (**3d**).

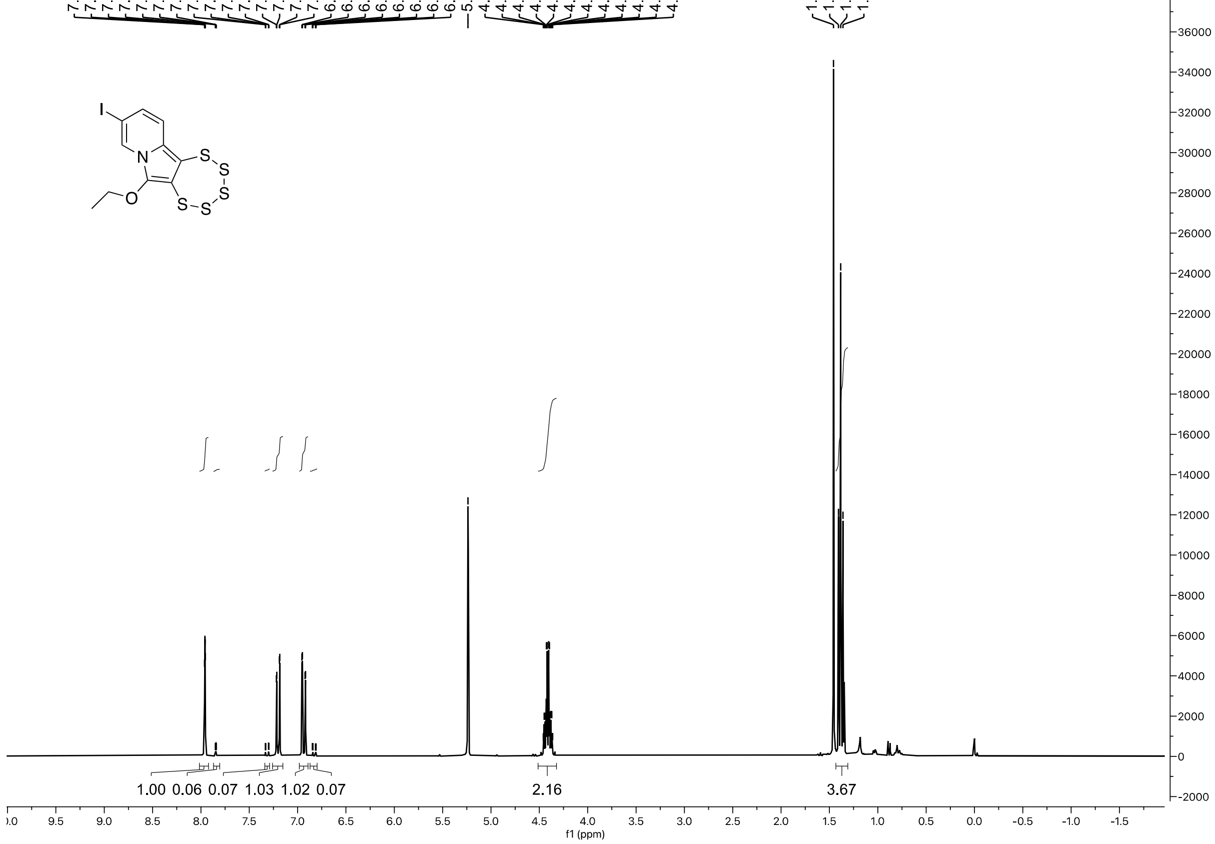

Figure S25. ^{1}H NMR spectrum of 6-ethoxy-9-iodo-[1,2,3,4,5]pentathiepino[6,7-a]indolizine (**3e**).

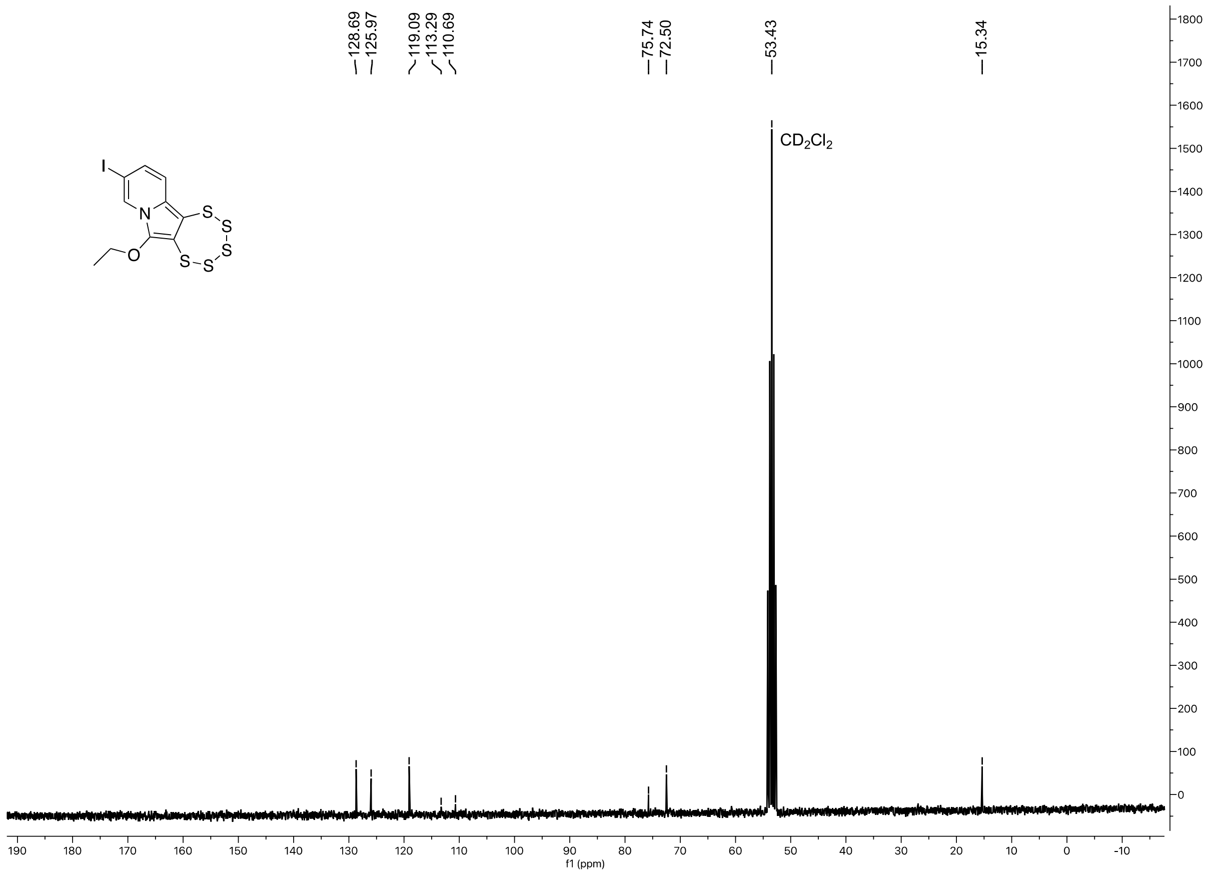

Figure S26. ^{13}C NMR spectrum of 6-ethoxy-9-iodo-[1,2,3,4,5]pentathiepino[6,7-a]indolizine (**3e**).

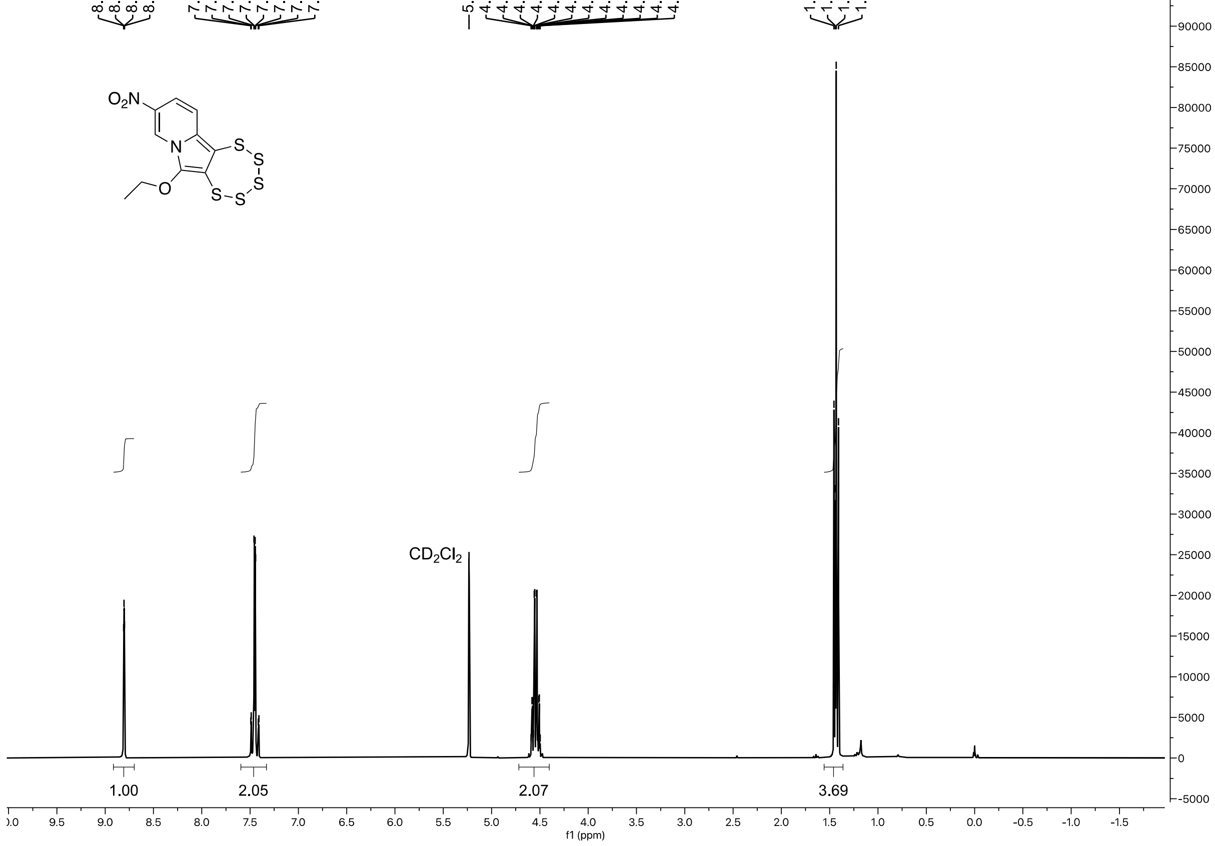

Figure S27. ^{1}H NMR spectrum of 6-ethoxy-9-nitro-[1,2,3,4,5]pentathiepino[6,7-a]indolizine (**3f**).

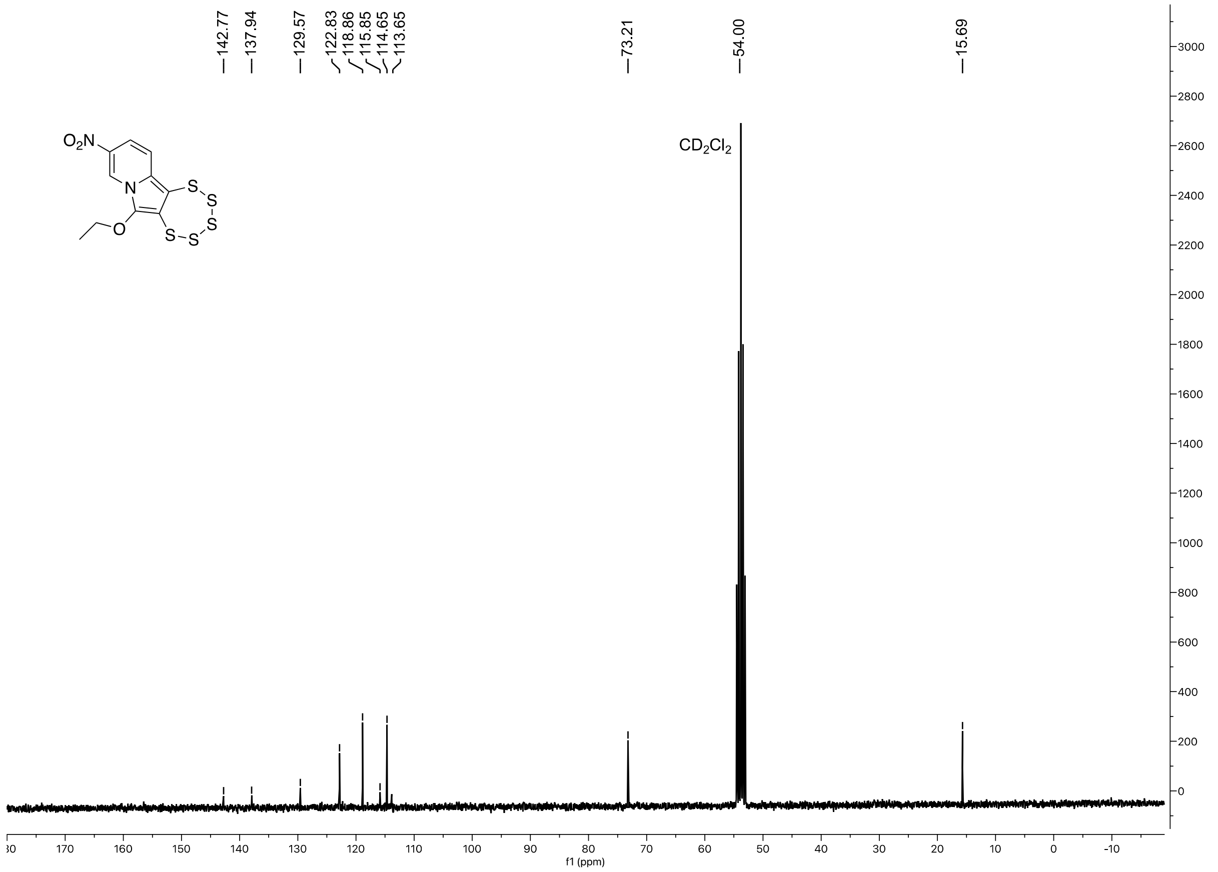

Figure S28. ^{13}C NMR spectrum of 6-ethoxy-9-nitro-[1,2,3,4,5]pentathiepino[6,7-a]indolizine (**3f**).

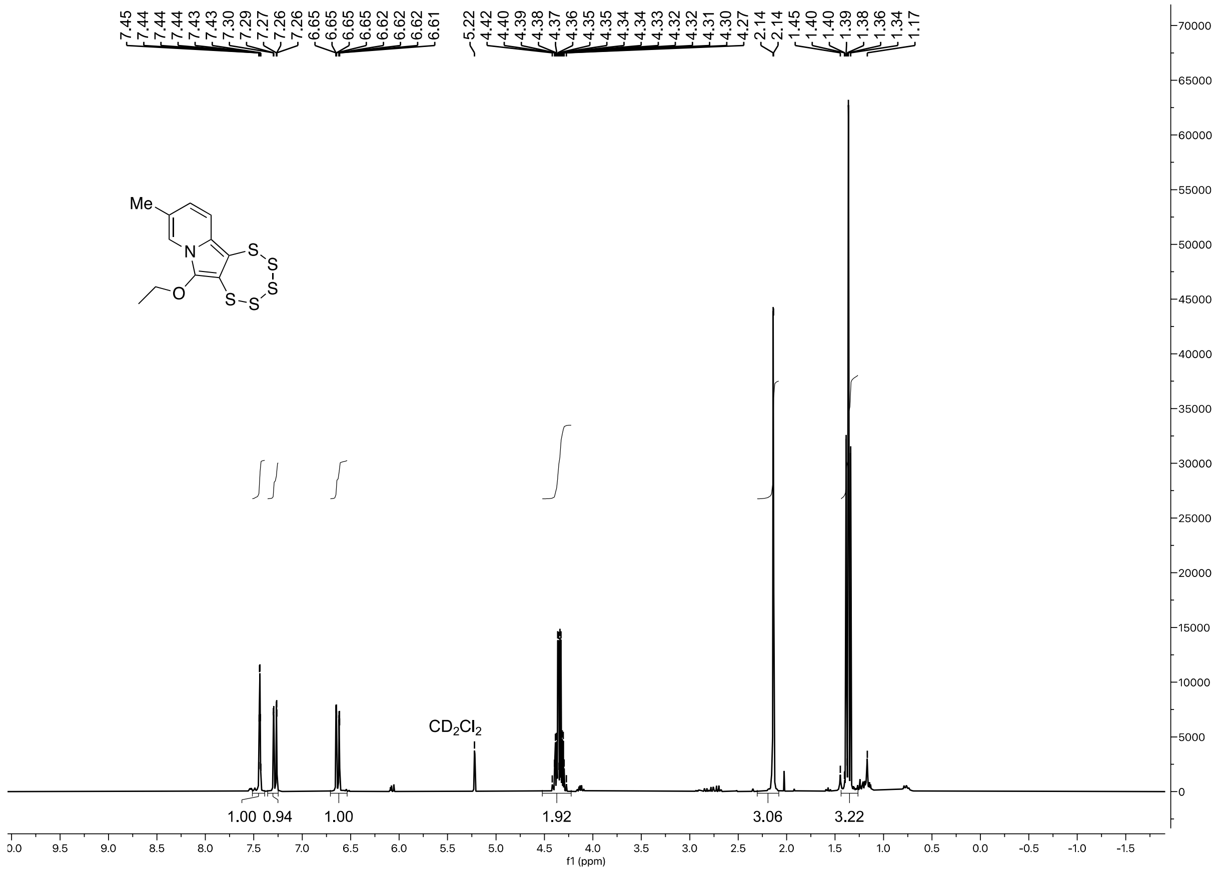

Figure S29. ^{1}H NMR spectrum of 6-ethoxy-9-methyl-[1,2,3,4,5]pentathiepino[6,7-a]indolizine (**3g**).

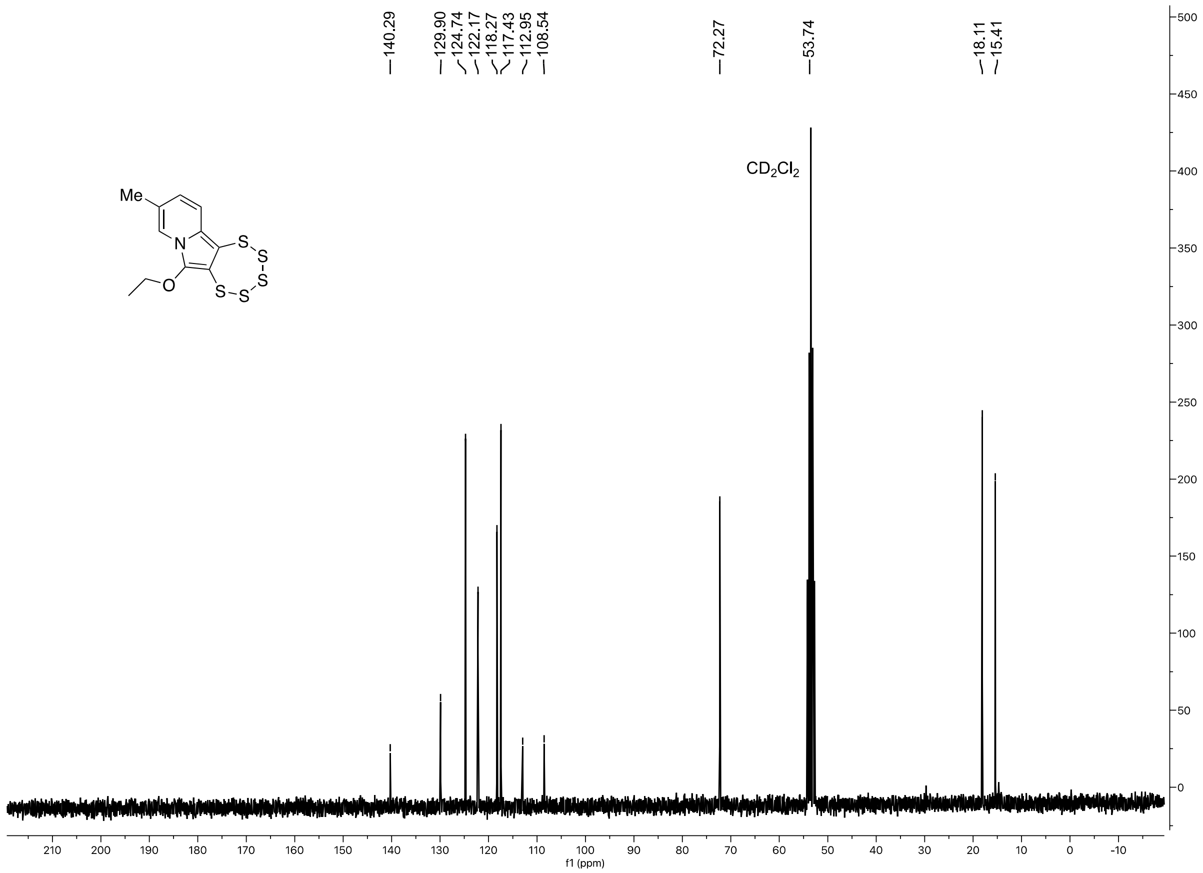

Figure S30. ^{13}C NMR spectrum of 6-ethoxy-9-methyl-[1,2,3,4,5]pentathiepino[6,7-a]indolizine (**3g**).

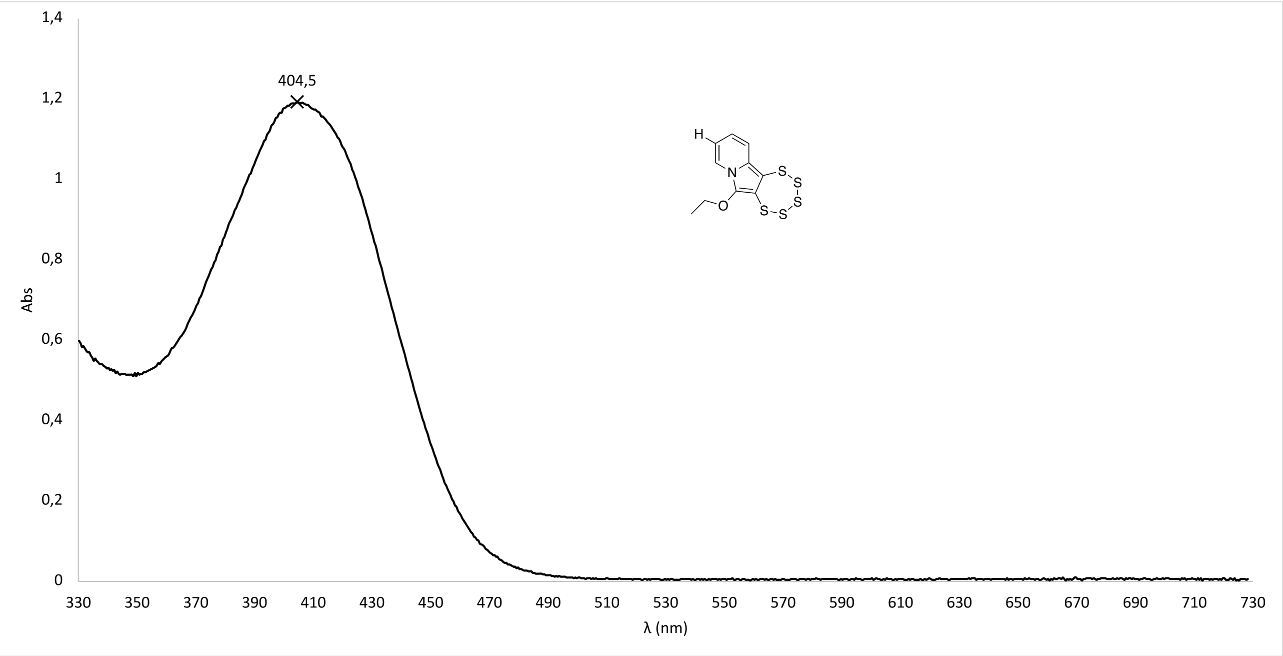

Figure S31. UV-Vis spectrum of 6-ethoxy-[1,2,3,4,5]pentathiepino[6,7-a]indolizine (**3a**).

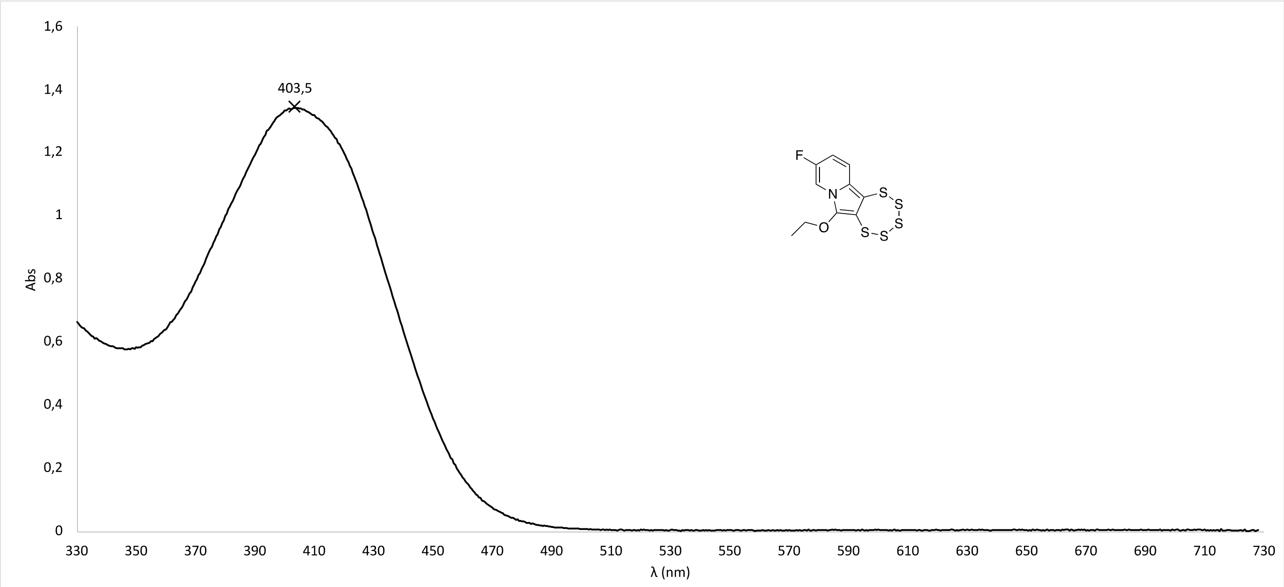

Figure S32. UV-Vis spectrum of 6-ethoxy-9-fluoro-[1,2,3,4,5]pentathiepino[6,7-a]indolizine (**3b**).

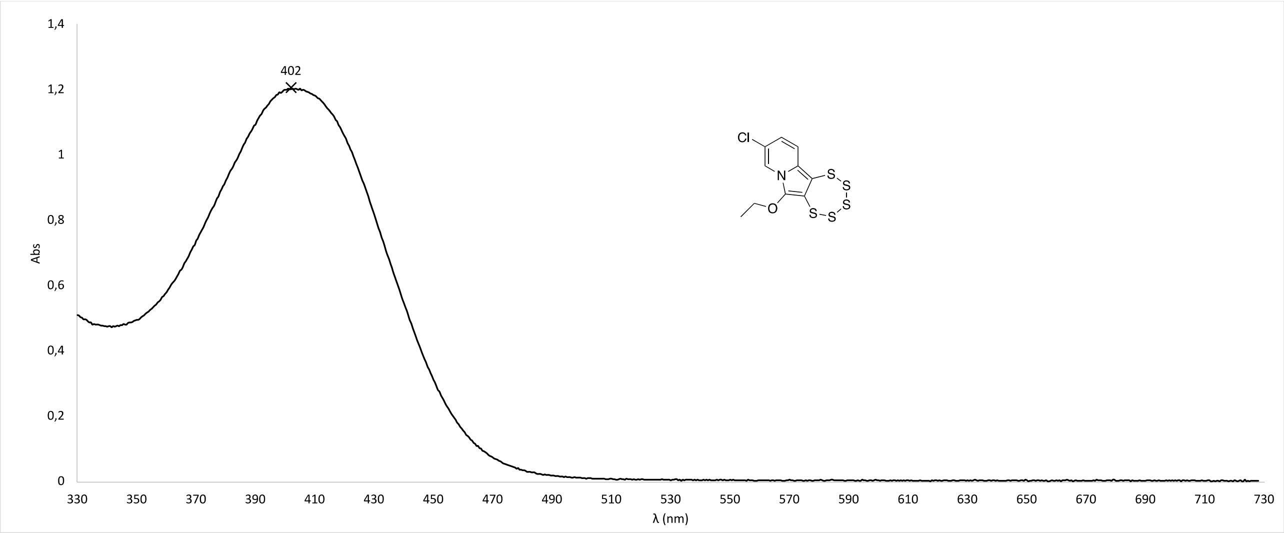

Figure S33. UV-Vis spectrum of 6-ethoxy-9-chloro-[1,2,3,4,5]pentathiepino[6,7-a]indolizine (**3c**).

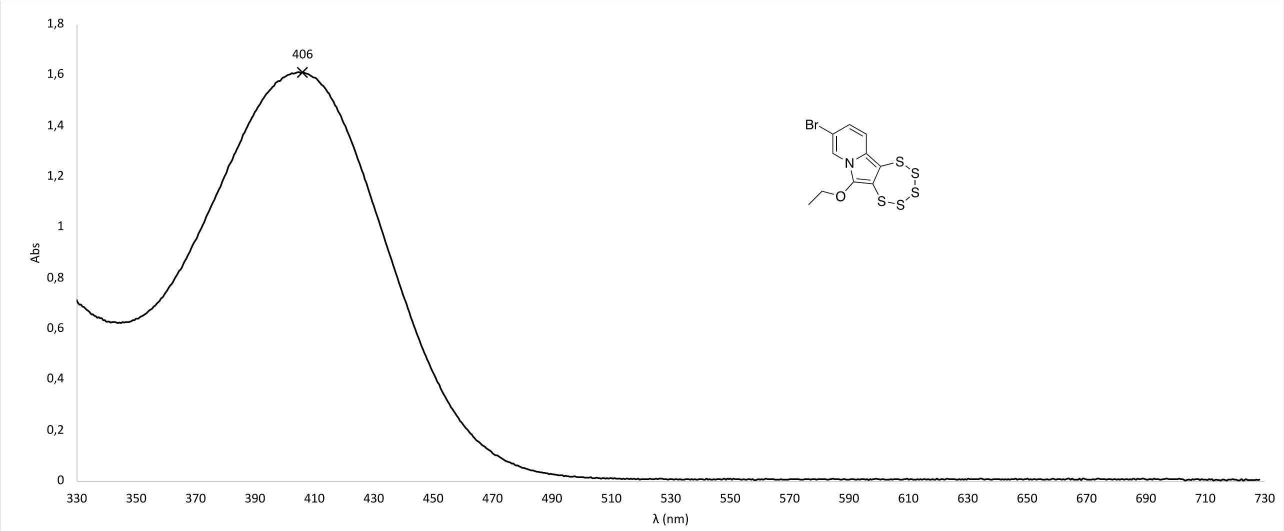

Figure S34. UV-Vis spectrum of 6-ethoxy-9-bromo-[1,2,3,4,5]pentathiepino[6,7-a]indolizine (**3d**).

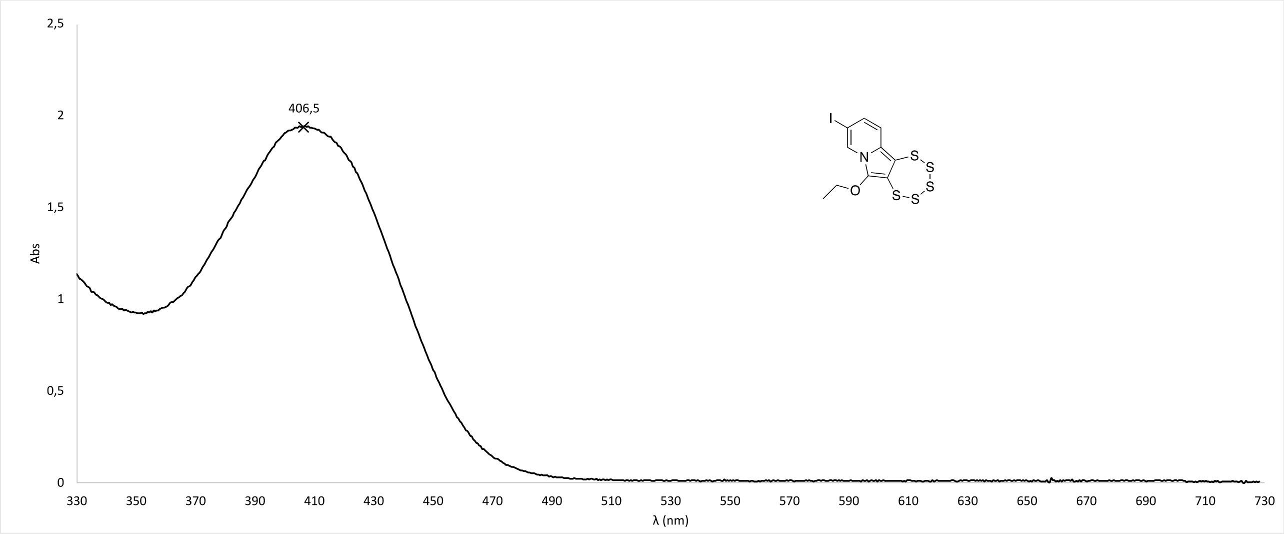

Figure S35. UV-Vis spectrum of 6-ethoxy-9-iodo-[1,2,3,4,5]pentathiepino[6,7-a]indolizine (**3e**).

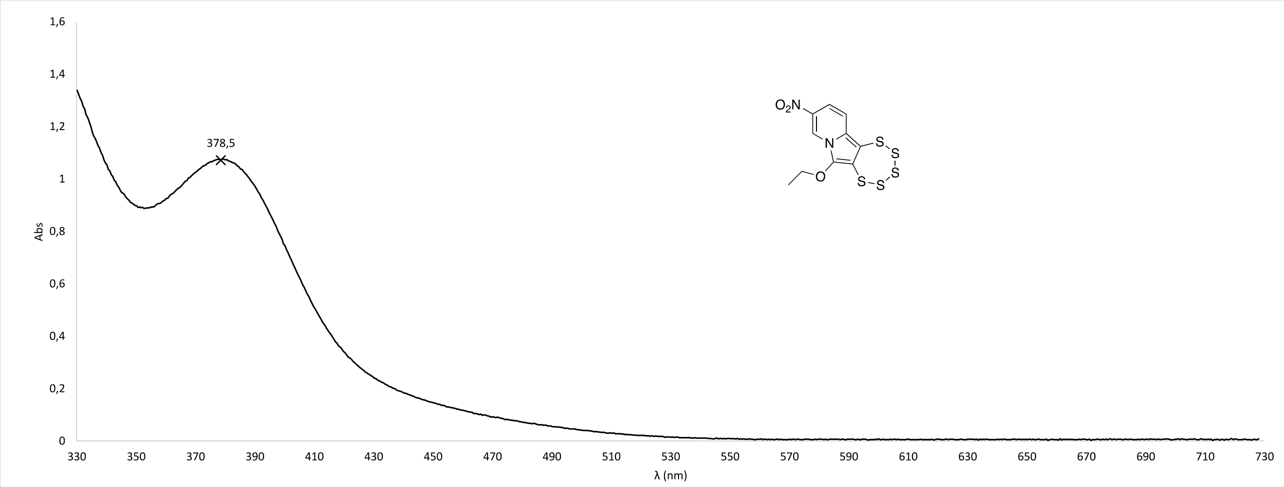

Figure S36. UV-Vis spectrum of 6-ethoxy-9-nitro-[1,2,3,4,5]pentathiepino[6,7-a]indolizine (**3f**).

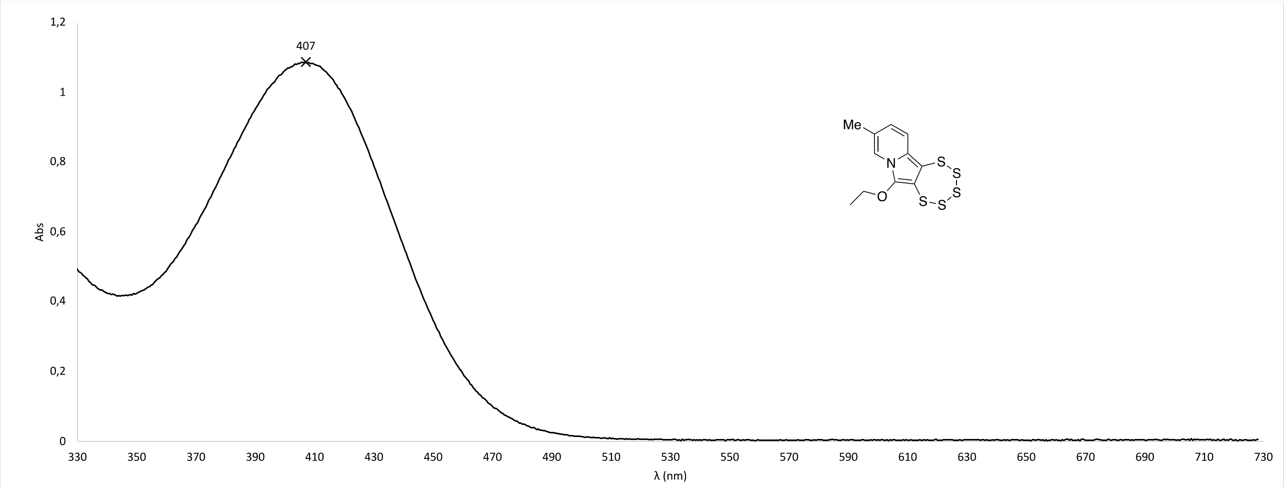

Figure S37. UV-Vis spectrum of 6-ethoxy-9-methyl-[1,2,3,4,5]pentathiepino[6,7-a]indolizine (**3g**).

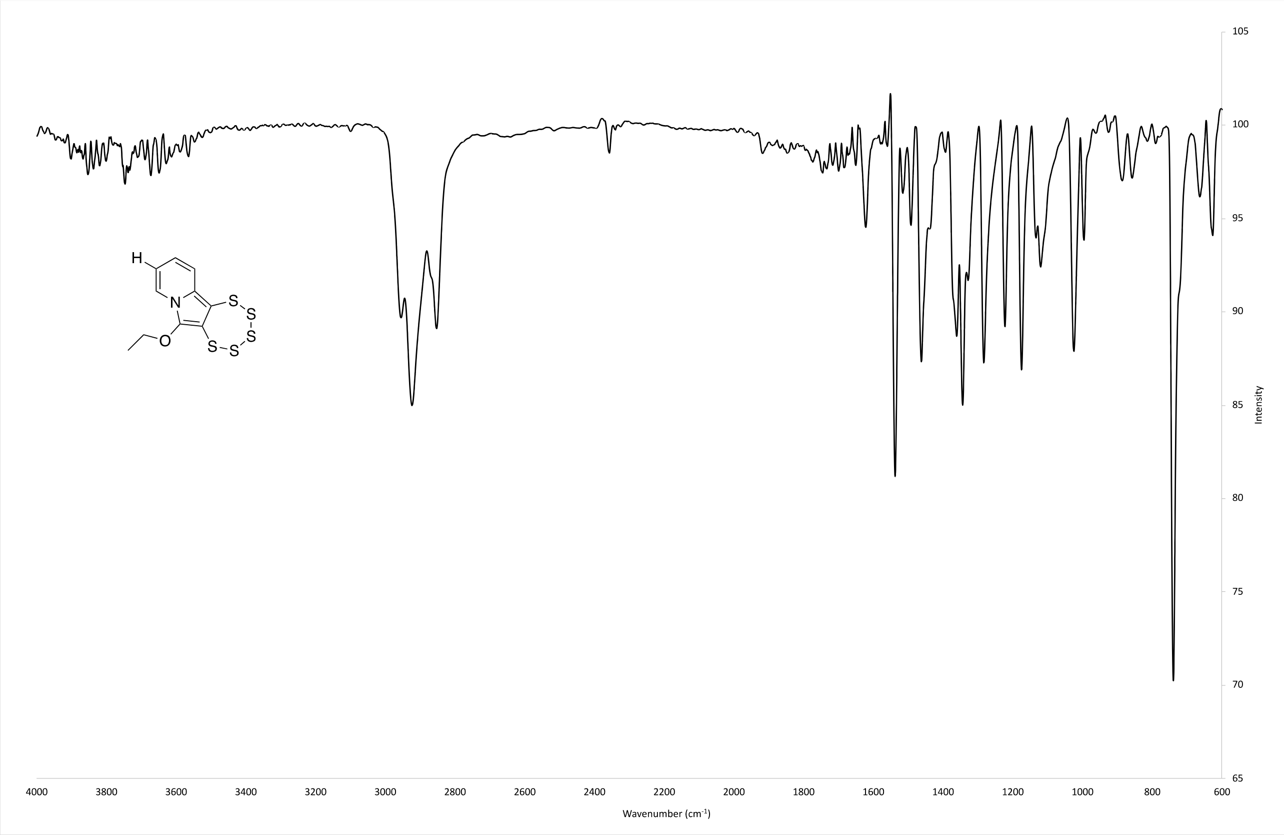

Figure S38. IR spectrum of 6-ethoxy-[1,2,3,4,5]pentathiepino[6,7-a]indolizine (**3a**).

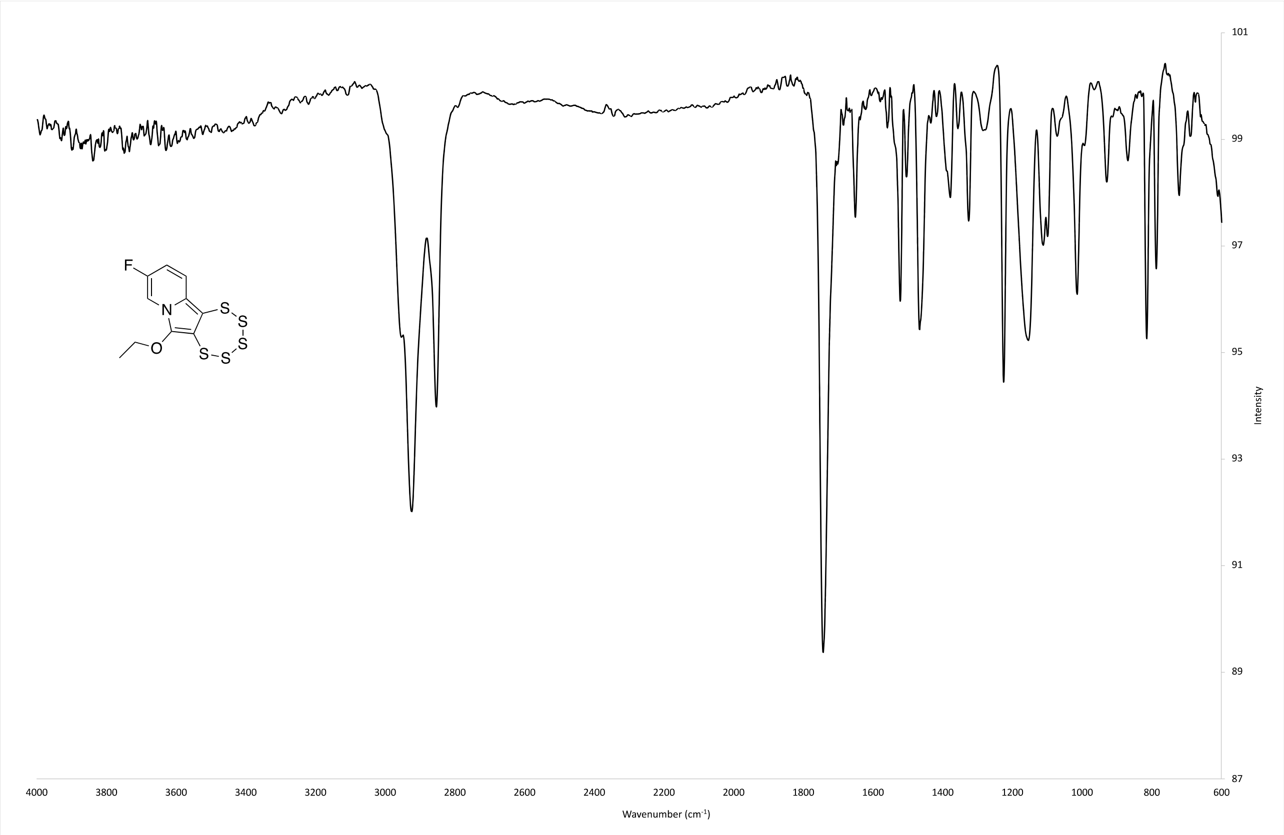

Figure S39. IR spectrum of 6-ethoxy-9-fluoro-[1,2,3,4,5]pentathiepino[6,7-a]indolizine (**3b**).

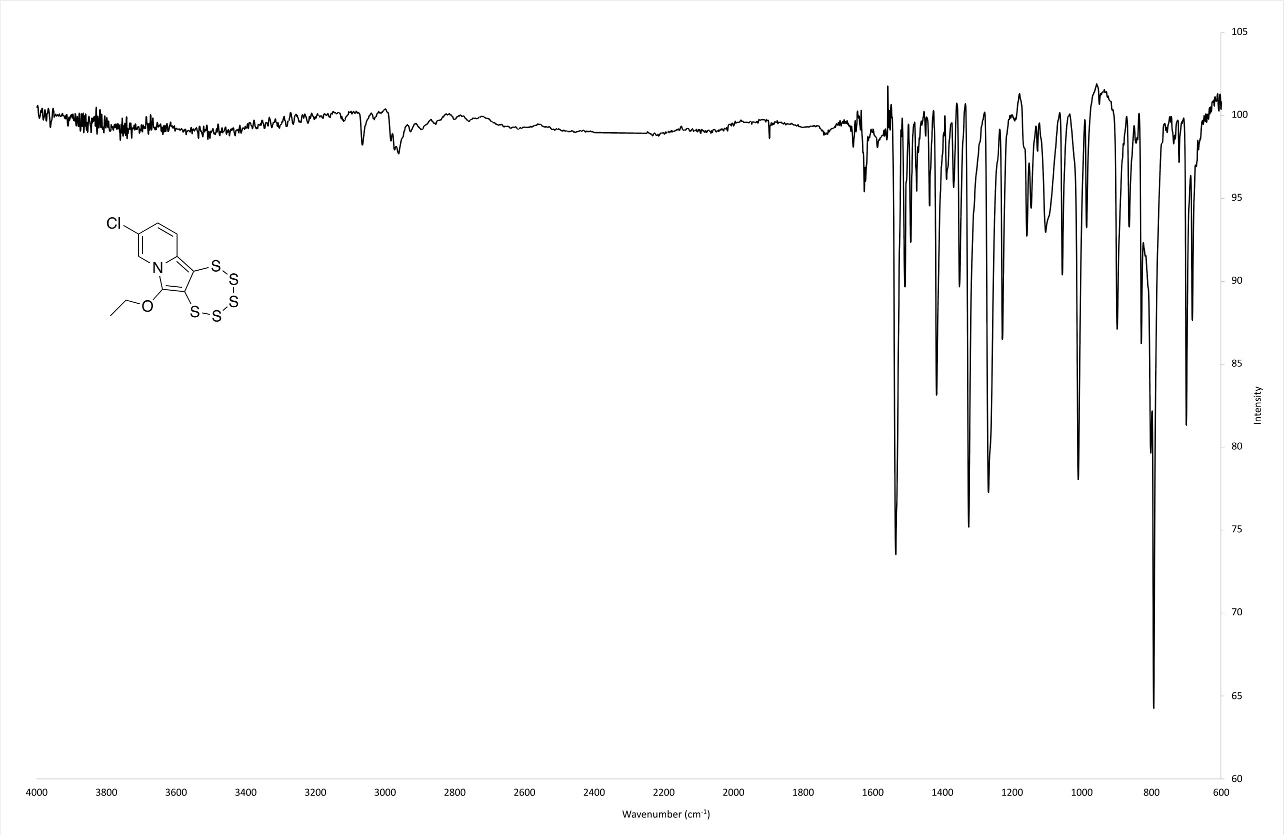

Figure S40. IR spectrum of 6-ethoxy-9-chloro-[1,2,3,4,5]pentathiepino[6,7-a]indolizine (**3c**).

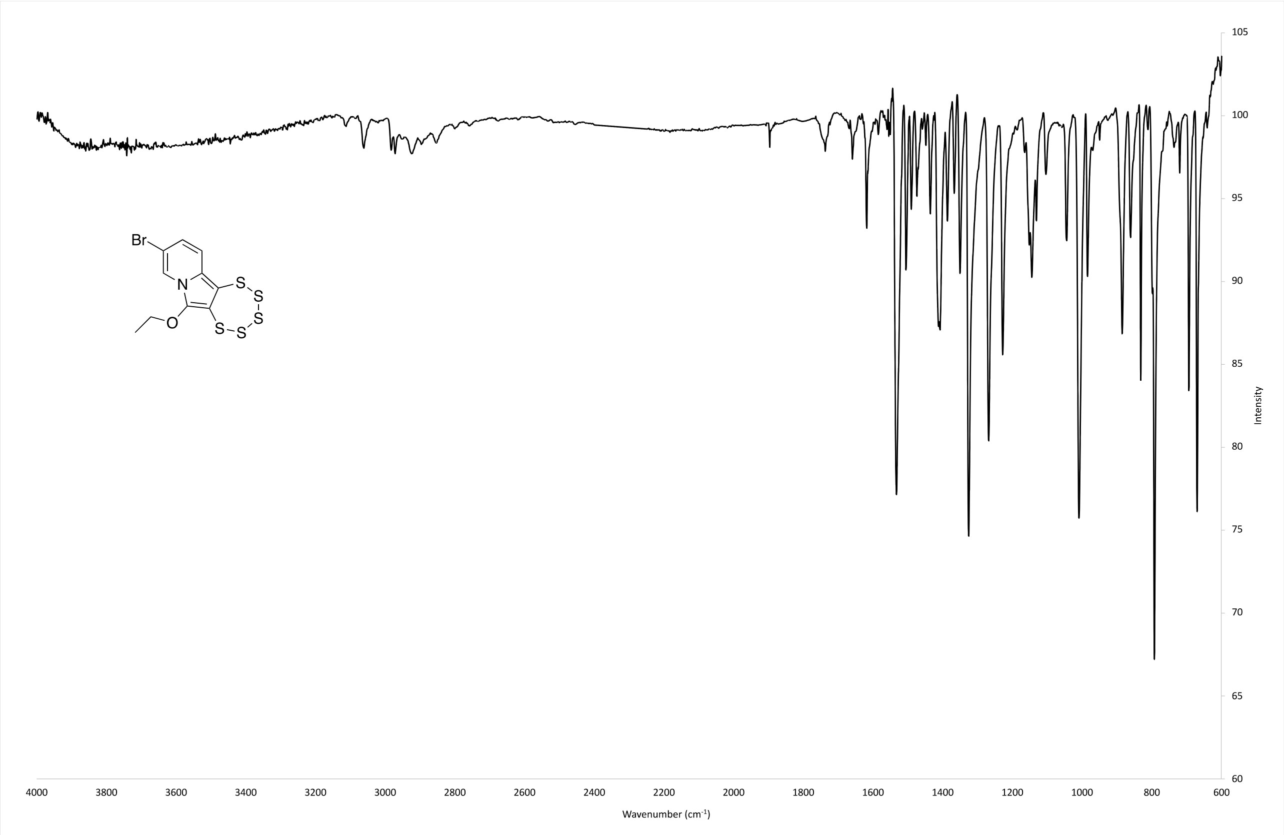

Figure S41. IR spectrum of 6-ethoxy-9-bromo-[1,2,3,4,5]pentathiepino[6,7-a]indolizine (**3d**).

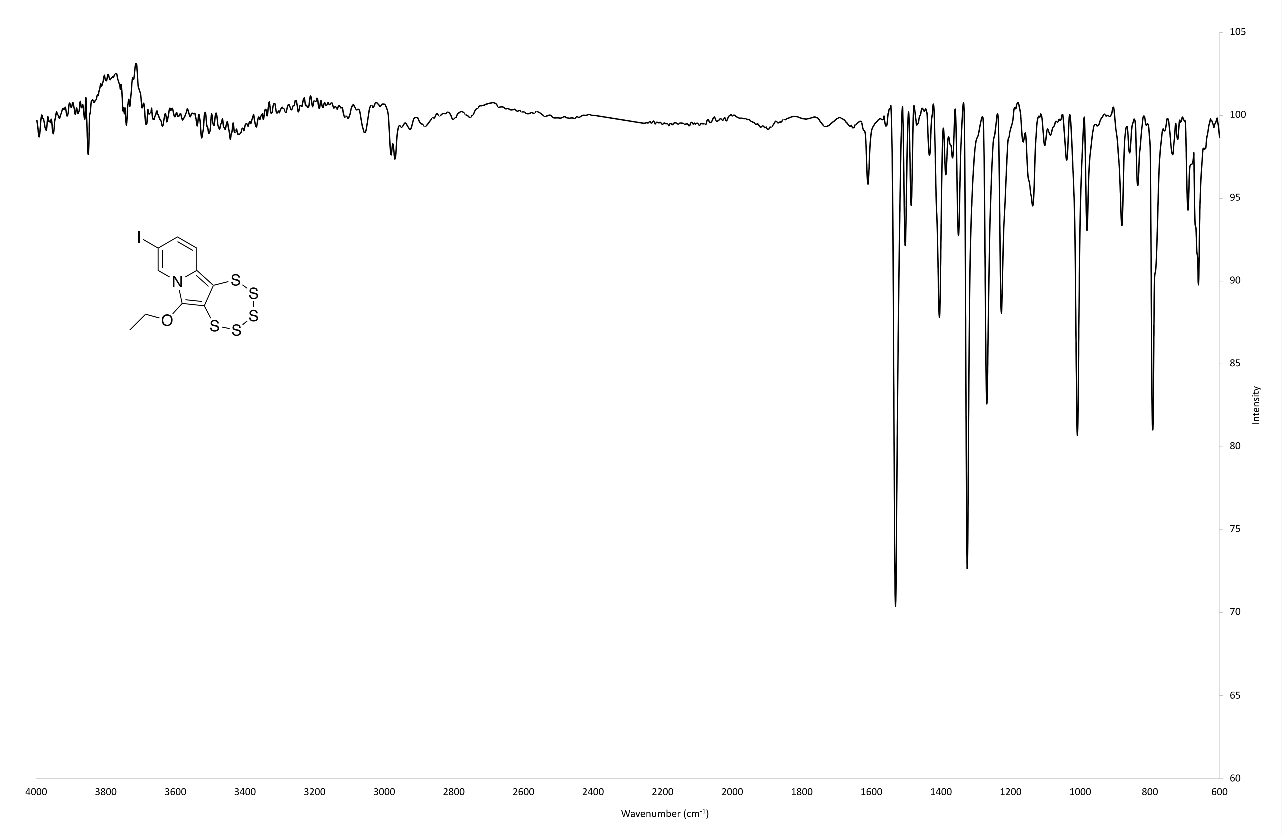

Figure S42. IR spectrum of 6-ethoxy-9-iodo-[1,2,3,4,5]pentathiepino[6,7-a]indolizine (**3e**).

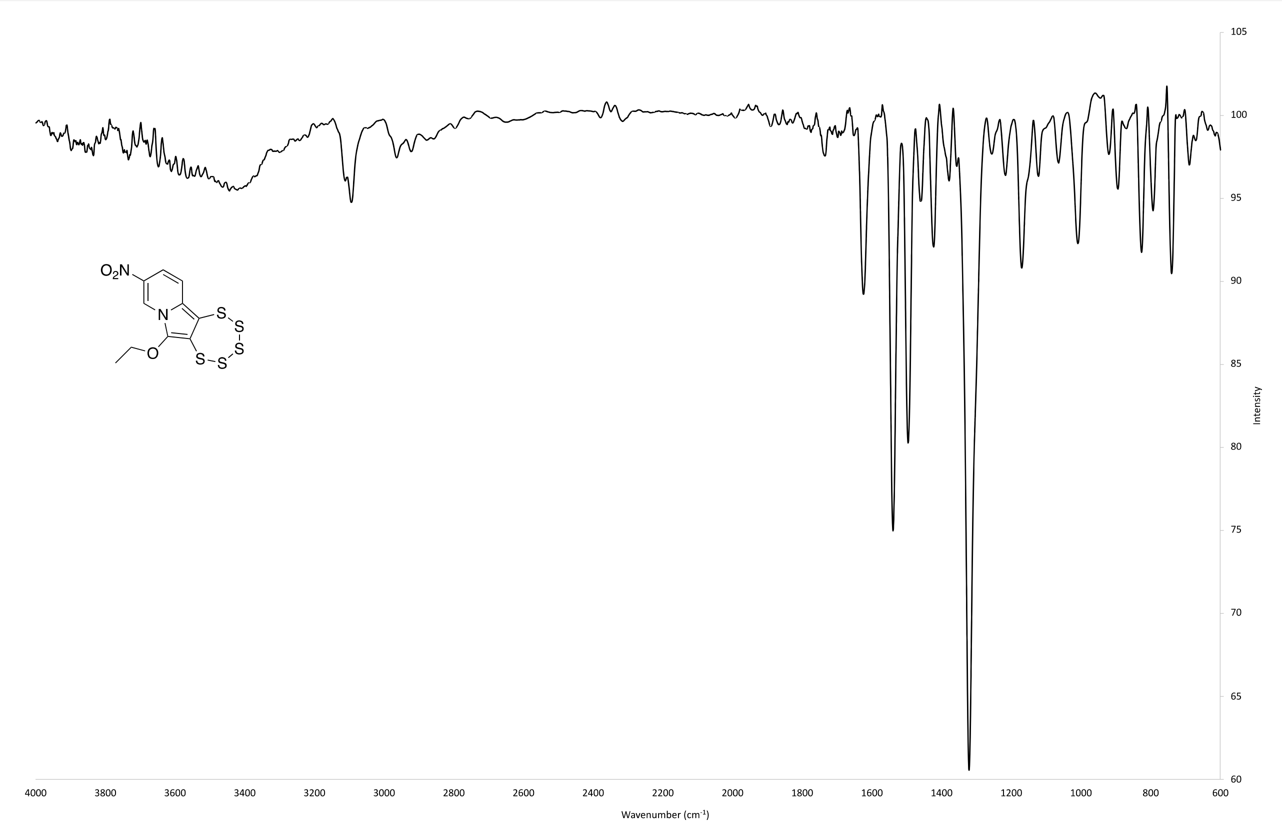

Figure S43. IR spectrum of 6-ethoxy-9-nitro-[1,2,3,4,5]pentathiepino[6,7-a]indolizine (**3f**).

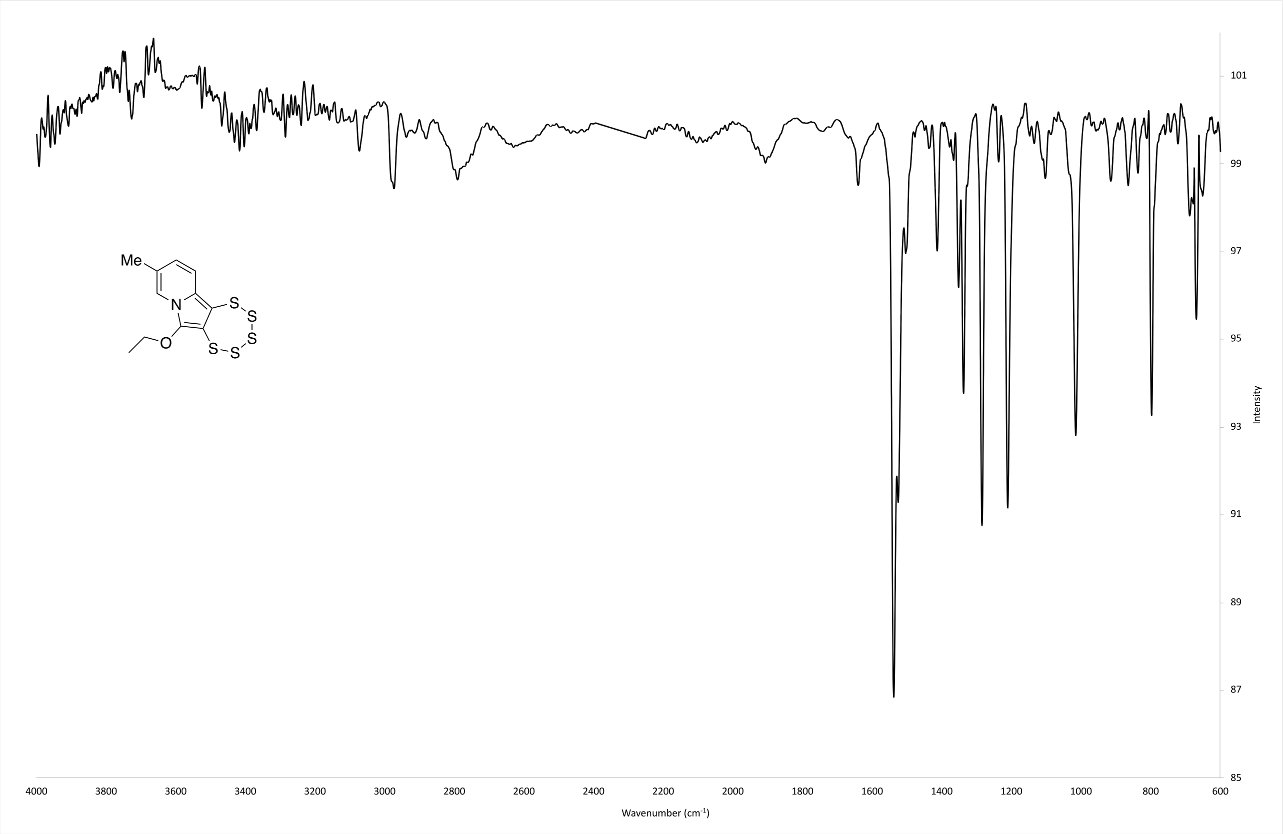

Figure S44. IR spectrum of 6-ethoxy-9-methyl-[1,2,3,4,5]pentathiepino[6,7-a]indolizine (**3g**).

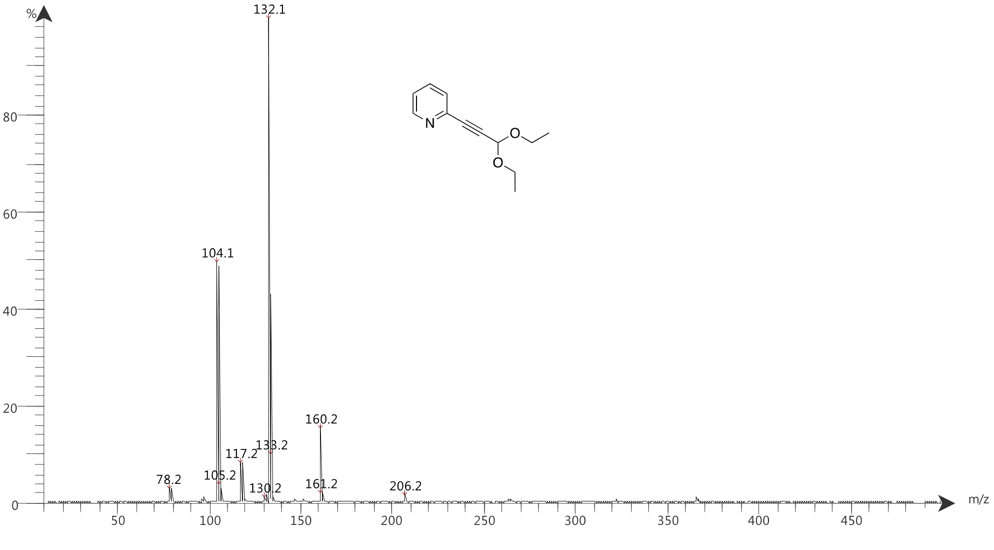

Figure S45. APCI Mass spectrum of 2-(3,3-diethoxyprop-1-yn-1-yl)pyridine (**2a**).

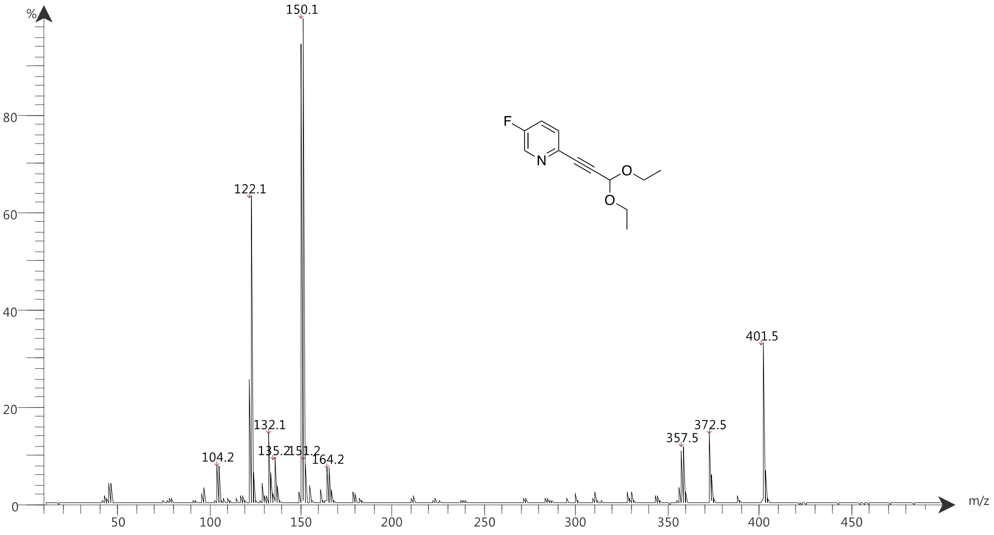

Figure S46. APCI Mass spectrum of 5-fluoro-2-(3,3-diethoxyprop-1-yn-1-yl)pyridine (**2b**).

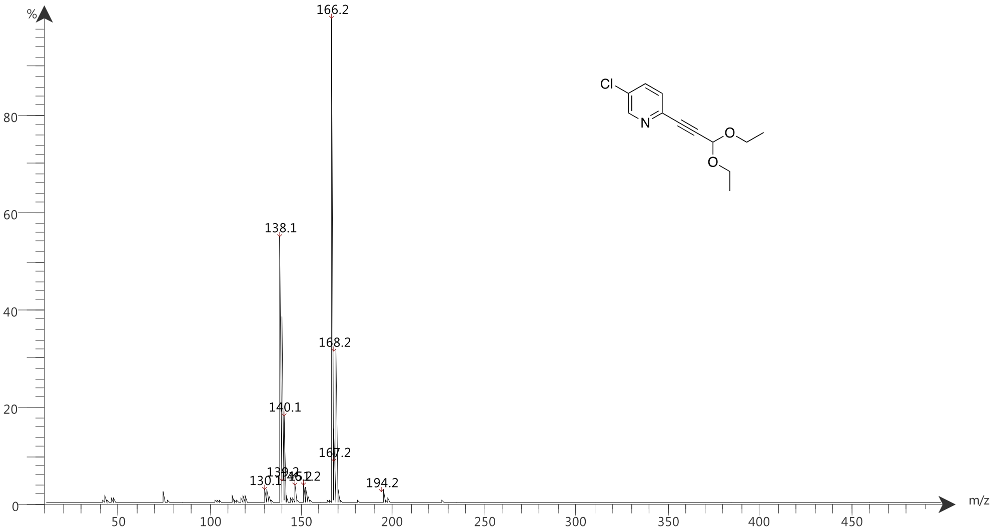

Figure S47. APCI Mass spectrum of 5-chloro-2-(3,3-diethoxyprop-1-yn-1-yl)pyridine (**2c**).

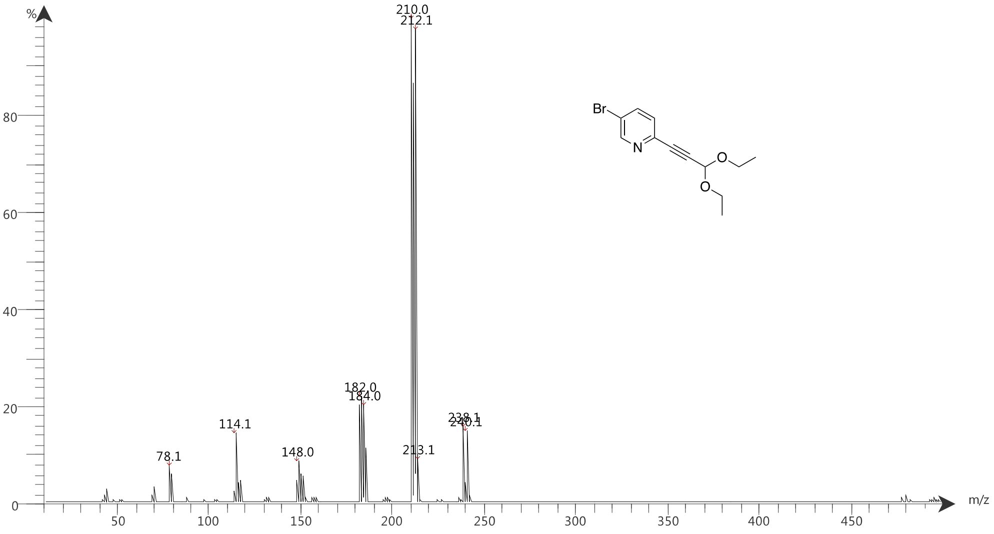

Figure S48. APCI Mass spectrum of 5-bromo-2-(3,3-diethoxyprop-1-yn-1-yl)pyridine (**2d**).

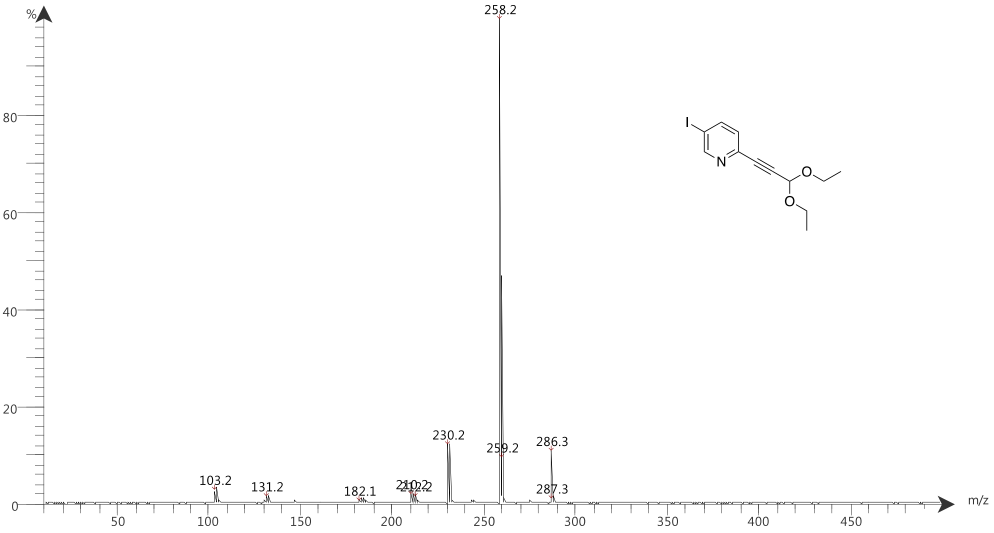

Figure S49. APCI Mass spectrum of 5-iodo-2-(3,3-diethoxyprop-1-yn-1-yl)pyridine (**2e**).

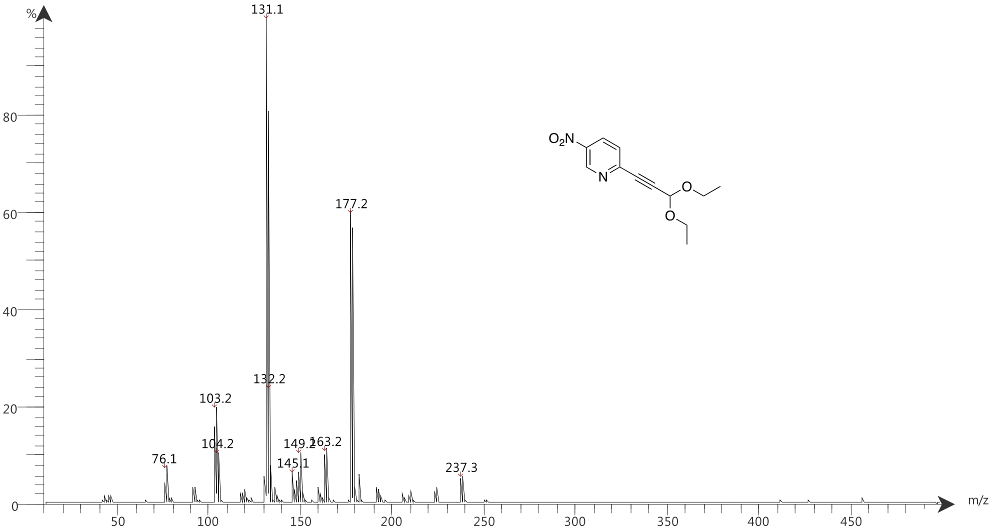

Figure S50. APCI Mass spectrum of 5-nitro-2-(3,3-diethoxyprop-1-yn-1-yl)pyridine (**2f**).

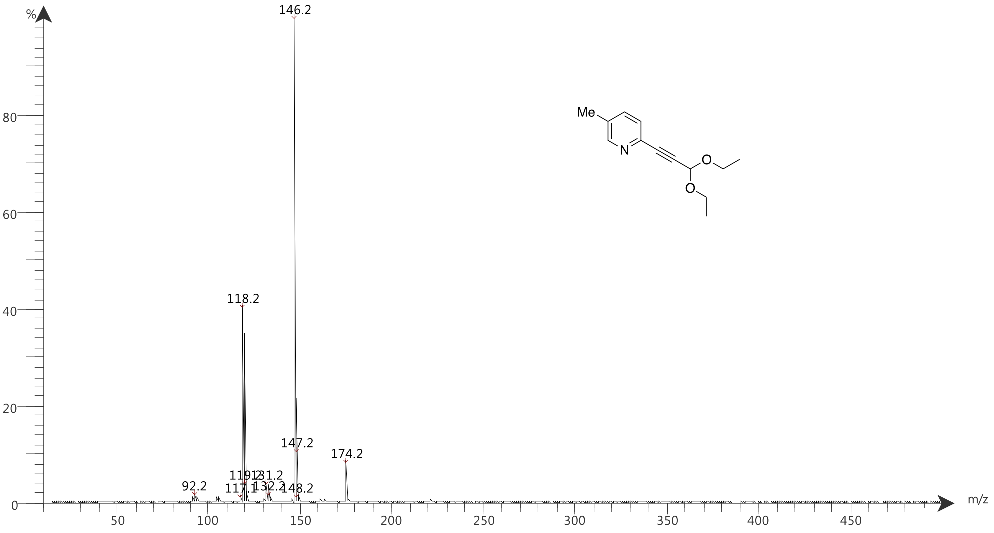

Figure S51. APCI Mass spectrum of 5-methyl-2-(3,3-diethoxyprop-1-yn-1-yl)pyridine (**2g**).

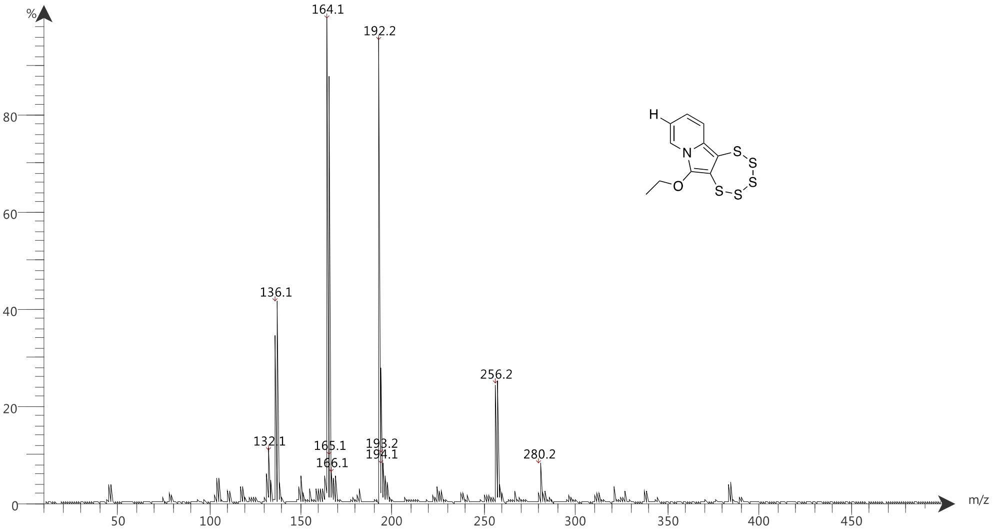

Figure S52. APCI Mass spectrum of 6-ethoxy-[1,2,3,4,5]pentathiepino[6,7-a]indolizine (**3a**).

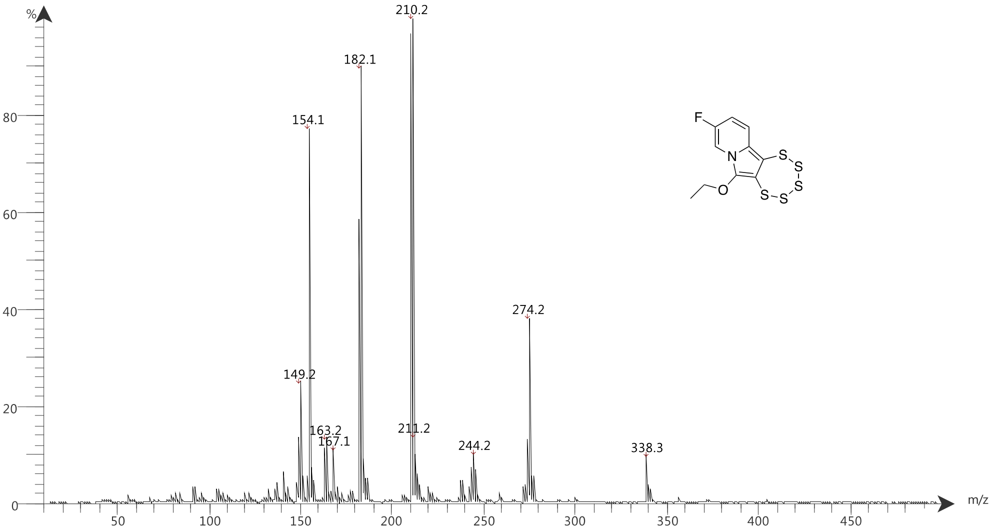

Figure S53. APCI Mass spectrum of 6-ethoxy-9-fluoro-[1,2,3,4,5]pentathiepino[6,7-a]indolizine (**3b**).

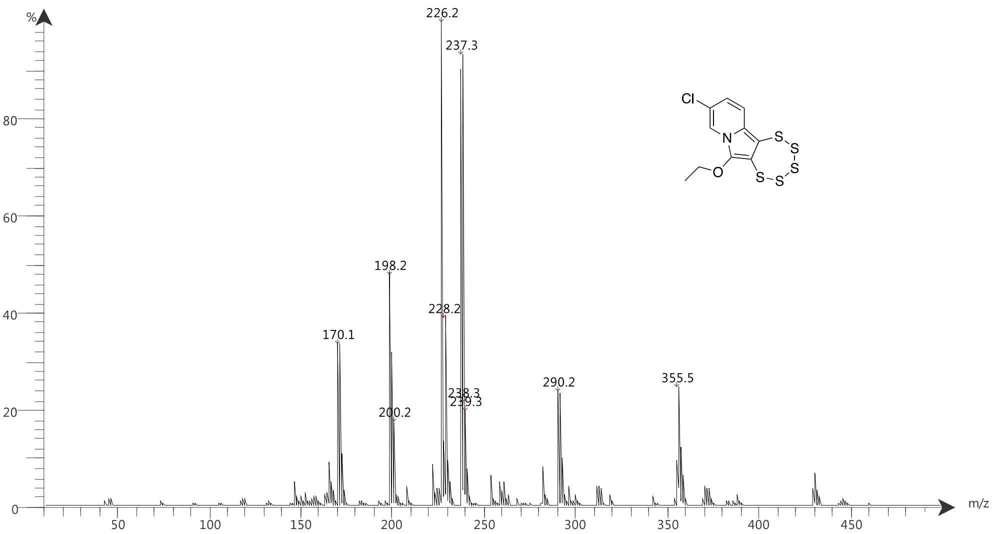

Figure S54. APCI Mass spectrum 6-ethoxy-9-chloro-[1,2,3,4,5]pentathiepino[6,7-a]indolizine (**3c**).

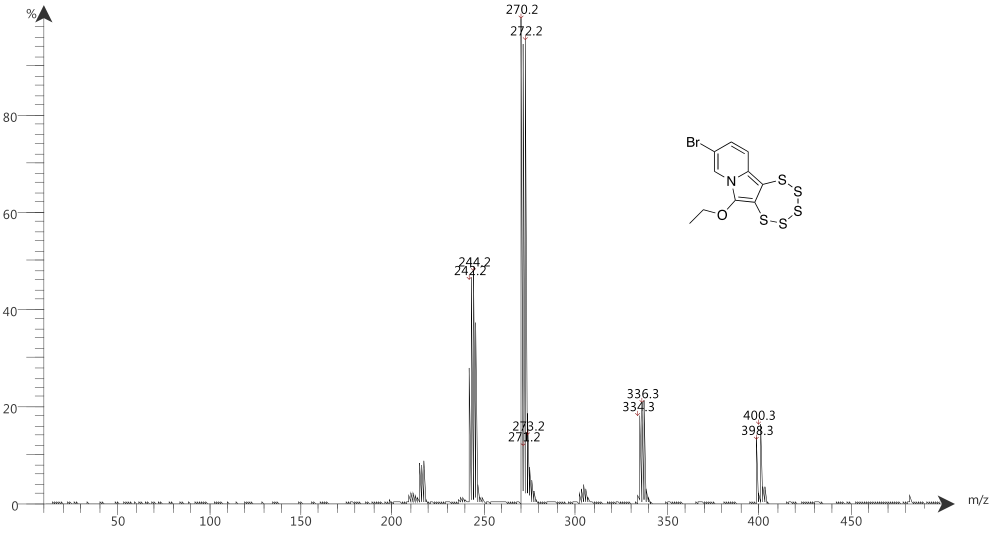

Figure S55. APCI Mass spectrum of 6-ethoxy-9-bromo-[1,2,3,4,5]pentathiepino[6,7-a]indolizine (**3d**).

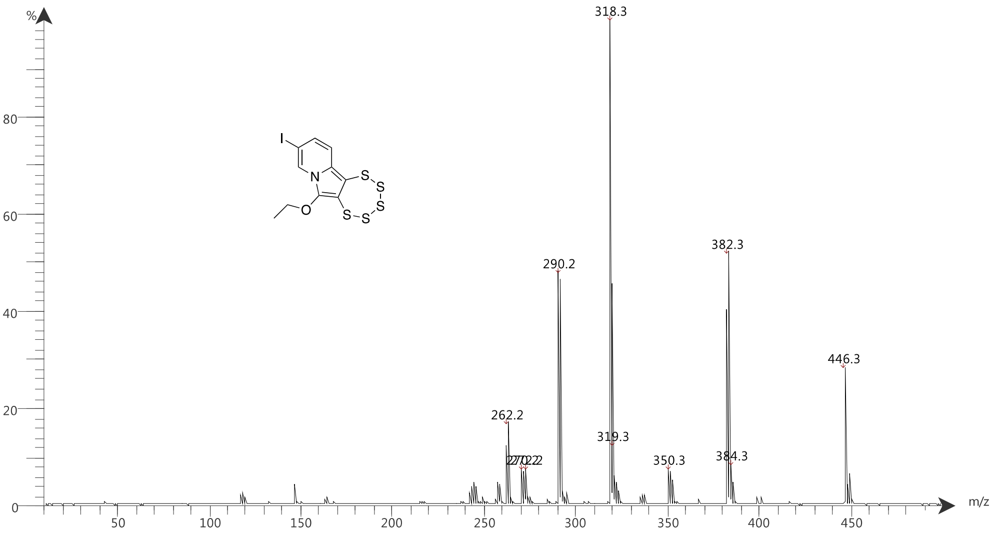

Figure S56. APCI Mass spectrum of 6-ethoxy-9-iodo-[1,2,3,4,5]pentathiepino[6,7-a]indolizine (**3e**).

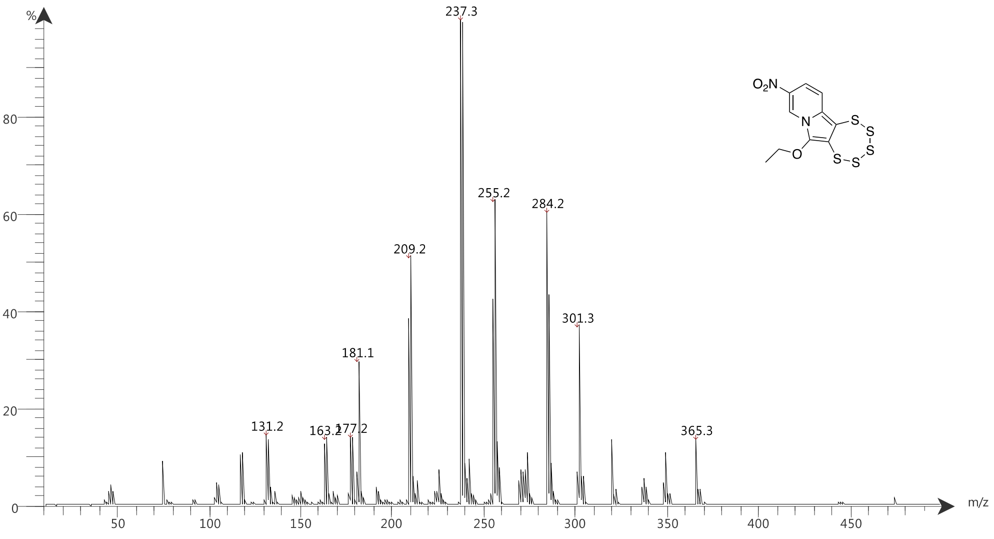

Figure S57. APCI Mass spectrum of 6-ethoxy-9-nitro-[1,2,3,4,5]pentathiepino[6,7-a]indolizine (**3f**).

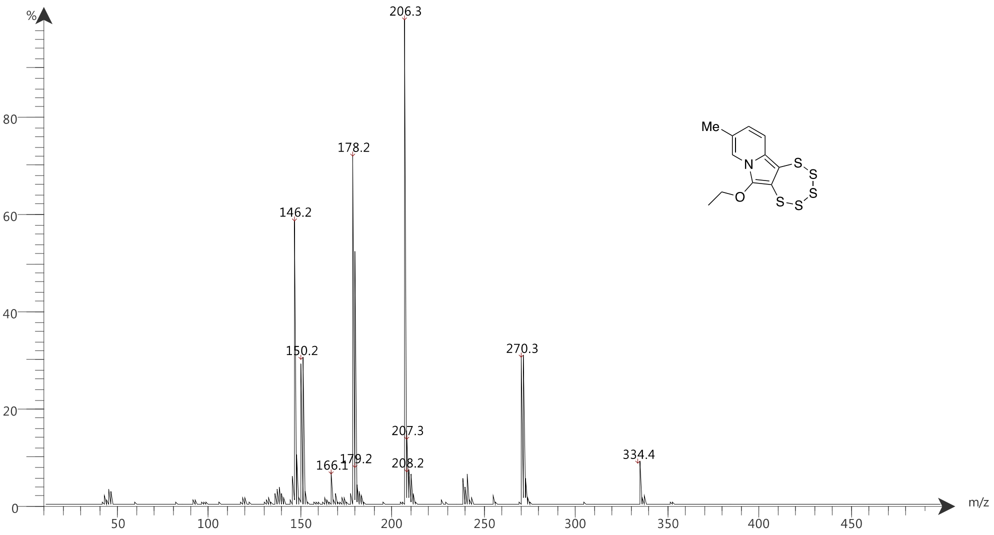

Figure S58. APCI Mass spectrum of 6-ethoxy-9-methyl-[1,2,3,4,5]pentathiepino[6,7-a]indolizine (**3g**).

Table S1. Comparison Between Dipole Moments and HOMO LUMO ENERGIES **3a** and **3f**

	**Dipole Moment** (D)	**HOMO – LUMO gap** (eV)	**HOMO** (eV)	**LUMO** (eV)
**3a**	5.41527	3.757	-5.597	-1.840
**3f**	2.36905	3.150	-6.100	-2.905

Table S2. Mulliken Atomic Charges for **3a**

```
-----------------------
MULLIKEN ATOMIC CHARGES
-----------------------
   0 S :   -0.037408
   1 S :   -0.048672
   2 S :   -0.021227
   3 S :   -0.050599
   4 S :   -0.050192
   5 O :   -0.329775
   6 N :    0.038046
   7 C :    0.197727
   8 C :   -0.017454
   9 C :   -0.031557
  10 C :    0.131295
  11 C :   -0.234629
  12 H :    0.120455
  13 C :   -0.076850
  14 H :    0.132511
  15 C :   -0.121277
  16 H :    0.128078
  17 C :   -0.115245
  18 H :    0.157232
  19 C :   -0.014908
  20 H :    0.096235
  21 H :    0.111672
  22 C :   -0.298121
  23 H :    0.107747
  24 H :    0.109827
  25 H :    0.117088
Sum of atomic charges:    0.0000000
```

Table S3. Summary of TD-DFT calculated first 20 low energy transitions of **3a**.

```
-----------------------------------
TD-DFT/TDA EXCITED STATES (SINGLETS)
-----------------------------------
the weight of the individual excitations are printed if larger than 1.0e-02

STATE  1:  E=   0.121751 au      3.313 eV    26721.3 cm**-1 <S**2> =   0.000000
    81a -> 82a  :     0.915576 (c=  0.95685750)
    81a -> 85a  :     0.014607 (c= -0.12086132)

STATE  2:  E=   0.139057 au      3.784 eV    30519.6 cm**-1 <S**2> =   0.000000
    80a -> 82a  :     0.586347 (c=  0.76573311)
    80a -> 84a  :     0.015351 (c=  0.12390116)
    81a -> 83a  :     0.120748 (c= -0.34748858)
    81a -> 84a  :     0.214701 (c=  0.46335830)
    81a -> 85a  :     0.032526 (c=  0.18035000)

STATE  3:  E=   0.139643 au      3.800 eV    30648.2 cm**-1 <S**2> =   0.000000
    79a -> 82a  :     0.012511 (c=  0.11185280)
    80a -> 82a  :     0.067112 (c= -0.25905925)
```

```
   81a -> 83a  :     0.840163 (c= -0.91660396)
   81a -> 84a  :     0.066073 (c= -0.25704572)

STATE  4:  E=   0.143515 au      3.905 eV    31497.8 cm**-1 <S**2> =   0.000000
   76a -> 82a  :     0.020137 (c= -0.14190657)
   77a -> 82a  :     0.031865 (c=  0.17850718)
   78a -> 82a  :     0.037096 (c=  0.19260428)
   80a -> 82a  :     0.253167 (c= -0.50315710)
   80a -> 84a  :     0.014638 (c= -0.12098814)
   81a -> 84a  :     0.609223 (c=  0.78052755)

STATE  5:  E=   0.148861 au      4.051 eV    32671.1 cm**-1 <S**2> =   0.000000
   78a -> 82a  :     0.163018 (c= -0.40375443)
   79a -> 82a  :     0.744221 (c= -0.86268224)
   79a -> 84a  :     0.013962 (c= -0.11816094)

STATE  6:  E=   0.151269 au      4.116 eV    33199.7 cm**-1 <S**2> =   0.000000
   77a -> 82a  :     0.117265 (c= -0.34244029)
   78a -> 82a  :     0.648098 (c=  0.80504517)
   78a -> 83a  :     0.013808 (c= -0.11750697)
   78a -> 84a  :     0.020186 (c=  0.14207894)
   79a -> 82a  :     0.111095 (c= -0.33330974)
   81a -> 85a  :     0.026331 (c=  0.16226715)

STATE  7:  E=   0.153658 au      4.181 eV    33724.1 cm**-1 <S**2> =   0.000000
   77a -> 82a  :     0.645829 (c=  0.80363462)
   77a -> 84a  :     0.024033 (c=  0.15502559)
   78a -> 82a  :     0.026997 (c=  0.16430716)
   80a -> 83a  :     0.011785 (c= -0.10855743)
   80a -> 84a  :     0.012221 (c= -0.11054811)
   81a -> 84a  :     0.041729 (c= -0.20427636)
   81a -> 85a  :     0.187689 (c=  0.43323047)

STATE  8:  E=   0.157891 au      4.296 eV    34653.1 cm**-1 <S**2> =   0.000000
   76a -> 82a  :     0.306961 (c= -0.55404064)
   77a -> 82a  :     0.054067 (c= -0.23252274)
   78a -> 82a  :     0.019402 (c= -0.13929140)
   79a -> 82a  :     0.014421 (c=  0.12008618)
   79a -> 83a  :     0.011566 (c=  0.10754493)
   80a -> 83a  :     0.073947 (c=  0.27193159)
   81a -> 85a  :     0.411717 (c=  0.64165148)
   81a -> 87a  :     0.030823 (c= -0.17556407)

STATE  9:  E=   0.163086 au      4.438 eV    35793.1 cm**-1 <S**2> =   0.000000
   76a -> 82a  :     0.016721 (c=  0.12930966)
   77a -> 82a  :     0.027943 (c=  0.16716156)
   78a -> 82a  :     0.013585 (c=  0.11655447)
   78a -> 83a  :     0.011881 (c= -0.10900139)
   79a -> 82a  :     0.015776 (c= -0.12560388)
   79a -> 83a  :     0.029631 (c=  0.17213758)
   80a -> 83a  :     0.751461 (c=  0.86686840)
   80a -> 84a  :     0.055288 (c=  0.23513354)
   81a -> 85a  :     0.021378 (c= -0.14621213)
   81a -> 86a  :     0.011602 (c=  0.10771432)

STATE 10:  E=   0.167296 au      4.552 eV    36717.2 cm**-1 <S**2> =   0.000000
   76a -> 82a  :     0.175387 (c= -0.41879253)
   80a -> 83a  :     0.011072 (c= -0.10522466)
```

81a -> 85a : 0.056952 (c= -0.23864671)
81a -> 86a : 0.631715 (c= 0.79480512)
81a -> 87a : 0.034334 (c= 0.18529360)

STATE 11: E= 0.169418 au 4.610 eV 37183.0 cm**-1 <S**2> = 0.000000
76a -> 82a : 0.277157 (c= 0.52645680)
77a -> 82a : 0.033774 (c= -0.18377696)
77a -> 83a : 0.042631 (c= -0.20647207)
77a -> 84a : 0.014471 (c= -0.12029369)
78a -> 83a : 0.023597 (c= 0.15361401)
79a -> 82a : 0.020067 (c= 0.14165746)
80a -> 83a : 0.011660 (c= 0.10798067)
80a -> 84a : 0.201723 (c= -0.44913596)
80a -> 85a : 0.016622 (c= -0.12892526)
80a -> 86a : 0.031449 (c= 0.17733807)
81a -> 85a : 0.049952 (c= 0.22349850)
81a -> 86a : 0.178213 (c= 0.42215252)
81a -> 87a : 0.033309 (c= -0.18250690)

STATE 12: E= 0.170696 au 4.645 eV 37463.5 cm**-1 <S**2> = 0.000000
76a -> 82a : 0.055371 (c= 0.23531092)
77a -> 83a : 0.091997 (c= 0.30331001)
78a -> 83a : 0.032568 (c= -0.18046699)
78a -> 84a : 0.028045 (c= -0.16746543)
79a -> 83a : 0.353332 (c= 0.59441751)
79a -> 84a : 0.011207 (c= 0.10586218)
80a -> 82a : 0.013759 (c= -0.11730060)
80a -> 83a : 0.053954 (c= -0.23228041)
80a -> 84a : 0.136327 (c= 0.36922503)
80a -> 86a : 0.032994 (c= -0.18164360)
81a -> 86a : 0.105160 (c= 0.32428374)
81a -> 87a : 0.045943 (c= -0.21434256)

STATE 13: E= 0.173621 au 4.724 eV 38105.3 cm**-1 <S**2> = 0.000000
77a -> 83a : 0.082967 (c= -0.28804053)
78a -> 83a : 0.411920 (c= 0.64180995)
78a -> 84a : 0.034839 (c= 0.18665259)
78a -> 86a : 0.010760 (c= -0.10373207)
79a -> 83a : 0.220962 (c= 0.47006566)
80a -> 85a : 0.119508 (c= 0.34569980)
80a -> 86a : 0.029720 (c= 0.17239489)
80a -> 87a : 0.010781 (c= -0.10383315)

STATE 14: E= 0.175267 au 4.769 eV 38466.7 cm**-1 <S**2> = 0.000000
78a -> 83a : 0.151780 (c= 0.38958987)
79a -> 83a : 0.135089 (c= -0.36754402)
79a -> 84a : 0.045319 (c= -0.21288194)
80a -> 82a : 0.018355 (c= -0.13548087)
80a -> 84a : 0.409743 (c= 0.64011190)
80a -> 85a : 0.066854 (c= -0.25856099)
81a -> 85a : 0.016134 (c= 0.12701873)
81a -> 86a : 0.014936 (c= 0.12221253)
81a -> 87a : 0.080163 (c= -0.28313013)

STATE 15: E= 0.177428 au 4.828 eV 38941.0 cm**-1 <S**2> = 0.000000
76a -> 83a : 0.036155 (c= 0.19014470)
77a -> 83a : 0.556974 (c= 0.74630706)
77a -> 84a : 0.035275 (c= 0.18781654)

```
   78a -> 83a  :     0.234776 (c=  0.48453672)
   80a -> 83a  :     0.023436 (c=  0.15308965)
   80a -> 84a  :     0.050573 (c= -0.22488338)
   80a -> 86a  :     0.011805 (c= -0.10865073)

STATE 16:  E=   0.178917 au      4.869 eV    39267.8 cm**-1 <S**2> =   0.000000
   77a -> 85a  :     0.014955 (c= -0.12229248)
   78a -> 83a  :     0.017800 (c= -0.13341634)
   78a -> 84a  :     0.056349 (c= -0.23737870)
   79a -> 82a  :     0.018475 (c=  0.13592170)
   79a -> 83a  :     0.030699 (c=  0.17521029)
   79a -> 84a  :     0.681173 (c= -0.82533185)
   79a -> 86a  :     0.022108 (c=  0.14868673)
   80a -> 85a  :     0.016923 (c=  0.13008783)
   80a -> 86a  :     0.063059 (c=  0.25111497)
   81a -> 86a  :     0.011900 (c= -0.10908813)

STATE 17:  E=   0.181747 au      4.946 eV    39888.9 cm**-1 <S**2> =   0.000000
   76a -> 82a  :     0.015765 (c=  0.12555910)
   77a -> 83a  :     0.014076 (c=  0.11864377)
   77a -> 84a  :     0.192503 (c= -0.43875111)
   77a -> 85a  :     0.054986 (c= -0.23449024)
   78a -> 84a  :     0.155703 (c= -0.39459227)
   79a -> 83a  :     0.079460 (c= -0.28188685)
   79a -> 84a  :     0.048320 (c=  0.21981701)
   79a -> 85a  :     0.010466 (c= -0.10230498)
   80a -> 84a  :     0.020824 (c=  0.14430544)
   80a -> 85a  :     0.169261 (c=  0.41141294)
   81a -> 85a  :     0.013171 (c=  0.11476456)
   81a -> 87a  :     0.126105 (c=  0.35511268)

STATE 18:  E=   0.184555 au      5.022 eV    40505.2 cm**-1 <S**2> =   0.000000
   75a -> 82a  :     0.013117 (c=  0.11452966)
   76a -> 85a  :     0.010614 (c= -0.10302406)
   77a -> 83a  :     0.079591 (c= -0.28211867)
   77a -> 84a  :     0.054635 (c=  0.23374192)
   77a -> 85a  :     0.077182 (c=  0.27781672)
   78a -> 82a  :     0.012813 (c=  0.11319505)
   78a -> 83a  :     0.023043 (c=  0.15179878)
   78a -> 84a  :     0.378026 (c= -0.61483781)
   78a -> 85a  :     0.099923 (c= -0.31610677)
   79a -> 85a  :     0.013858 (c= -0.11772092)
   79a -> 86a  :     0.026035 (c= -0.16135272)
   80a -> 85a  :     0.028516 (c= -0.16886634)
   80a -> 86a  :     0.069101 (c= -0.26287126)
   81a -> 87a  :     0.057853 (c=  0.24052750)

STATE 19:  E=   0.186116 au      5.064 eV    40847.7 cm**-1 <S**2> =   0.000000
   76a -> 82a  :     0.015075 (c=  0.12277856)
   76a -> 83a  :     0.013043 (c=  0.11420428)
   76a -> 84a  :     0.023543 (c= -0.15343696)
   76a -> 85a  :     0.024147 (c=  0.15539442)
   77a -> 84a  :     0.314366 (c=  0.56068349)
   77a -> 85a  :     0.014220 (c= -0.11924829)
   78a -> 85a  :     0.027143 (c= -0.16475075)
   78a -> 86a  :     0.017701 (c=  0.13304515)
   79a -> 83a  :     0.049668 (c= -0.22286249)
   79a -> 85a  :     0.111112 (c= -0.33333474)
```

80a -> 85a : 0.229704 (c= 0.47927413)
80a -> 86a : 0.013453 (c= 0.11598900)
81a -> 87a : 0.098794 (c= -0.31431562)

STATE 20: E= 0.187024 au 5.089 eV 41047.1 cm**-1 <S**2> = 0.000000
76a -> 83a : 0.066899 (c= -0.25864919)
77a -> 84a : 0.068375 (c= 0.26148679)
78a -> 84a : 0.077373 (c= -0.27815992)
78a -> 85a : 0.022836 (c= 0.15111514)
78a -> 86a : 0.024230 (c= 0.15566127)
79a -> 83a : 0.010465 (c= -0.10229671)
79a -> 85a : 0.612678 (c= 0.78273736)
80a -> 85a : 0.028459 (c= 0.16869942)
80a -> 86a : 0.028069 (c= 0.16753825)

ABSORPTION SPECTRUM VIA TRANSITION ELECTRIC DIPOLE MOMENTS

State	Energy (cm-1)	Wavelength (nm)	fosc	T2 (au**2)	TX (au)	TY (au)	TZ
1	26721.3	374.2	0.148618139	1.83100	0.10277	-1.34324	0.12708
2	30519.6	327.7	0.005001742	0.05395	0.11089	0.19256	0.06766
3	30648.2	326.3	0.006422274	0.06899	0.16758	-0.13113	0.15397
4	31497.8	317.5	0.014031813	0.14666	-0.00262	-0.35880	-0.13384
5	32671.1	306.1	0.009057343	0.09127	0.21828	-0.05854	0.20049
6	33199.7	301.2	0.003472390	0.03443	0.13931	-0.08281	0.09038
7	33724.1	296.5	0.002791267	0.02725	-0.05896	-0.14658	0.04781
8	34653.1	288.6	0.038314994	0.36400	-0.14321	-0.54521	-0.21502
9	35793.1	279.4	0.019364365	0.17811	0.08499	0.08405	0.40474
10	36717.2	272.4	0.023828239	0.21365	0.12002	0.43852	0.08332
11	37183.0	268.9	0.045218334	0.40035	-0.08595	-0.62110	-0.08484
12	37463.5	266.9	0.011333825	0.09960	-0.18194	-0.19824	-0.16491
13	38105.3	262.4	0.014501893	0.12529	0.11222	0.27470	0.19297
14	38466.7	260.0	0.017751950	0.15193	0.11678	-0.37181	-0.00684
15	38941.0	256.8	0.003139964	0.02655	0.03072	0.14530	0.06701
16	39267.8	254.7	0.001271196	0.01066	0.04046	0.09488	0.00432
17	39888.9	250.7	0.058227983	0.48057	0.08735	-0.65926	-0.19575
18	40505.2	246.9	0.021816308	0.17732	0.14110	0.29928	0.26046
19	40847.7	244.8	0.030296713	0.24418	0.11777	-0.45945	-0.13862
20	41047.1	243.6	0.002402289	0.01927	0.00817	-0.13820	0.01002

ABSORPTION SPECTRUM VIA TRANSITION VELOCITY DIPOLE MOMENTS

State	Energy (cm-1)	Wavelength (nm)	fosc	P2 (au**2)	PX (au)	PY (au)	PZ
1	26721.3	374.2	0.007888048	0.00144	0.00569	-0.00558	0.03711
2	30519.6	327.7	0.000594665	0.00012	-0.00145	0.00750	-0.00811
3	30648.2	326.3	0.002321943	0.00049	-0.01887	0.00740	0.00869
4	31497.8	317.5	0.010505104	0.00226	-0.02027	0.03464	-0.02552
5	32671.1	306.1	0.000512144	0.00011	-0.00840	0.00521	-0.00408
6	33199.7	301.2	0.008697934	0.00197	-0.01502	-0.03909	0.01483
7	33724.1	296.5	0.004047782	0.00093	0.02334	0.01950	-0.00282
8	34653.1	288.6	0.001534433	0.00036	-0.00816	0.00797	0.01527
9	35793.1	279.4	0.025352369	0.00620	0.05071	0.01561	-0.05820
10	36717.2	272.4	0.003780357	0.00095	-0.00002	-0.03074	0.00189

11	37183.0	268.9	0.009498863	0.00241	0.00341	0.04670	-0.01487
12	37463.5	266.9	0.000894688	0.00023	0.01266	-0.00071	0.00827
13	38105.3	262.4	0.015811580	0.00412	-0.02498	-0.05817	0.01052
14	38466.7	260.0	0.002608259	0.00069	-0.00274	0.02023	-0.01640
15	38941.0	256.8	0.004582063	0.00122	-0.00683	-0.03379	0.00559
16	39267.8	254.7	0.000593821	0.00016	-0.00842	-0.00585	0.00737
17	39888.9	250.7	0.013038121	0.00355	0.00729	0.02828	0.05197
18	40505.2	246.9	0.007758747	0.00215	-0.03784	-0.01117	-0.02431
19	40847.7	244.8	0.011617955	0.00324	-0.00647	0.05226	0.02170
20	41047.1	243.6	0.002271134	0.00064	0.01102	0.01791	-0.01396

Table S4. Electric properties calculations of **3a**.

```
The origin for moment calculation is the CENTER OF MASS  = ( 9.610306, 19.006217 12.966919)
-------------
DIPOLE MOMENT
-------------
                                X             Y             Z
Electronic contribution:    -4.04877     22.23335     -10.62197
Nuclear contribution   :     3.78769    -24.24490      11.27350
                        -----------------------------------------
Total Dipole Moment    :    -0.26108     -2.01155       0.65153
                        -----------------------------------------
Magnitude (a.u.)       :     2.13049
Magnitude (Debye)      :     5.41527

--------------------
Rotational spectrum
--------------------
Rotational constants in cm-1:     0.013645     0.008755     0.005837
Rotational constants in MHz :   409.064025   262.453777   174.982524

 Dipole components along the rotational axes:
x,y,z [a.u.] :    -1.997973    -0.098822     0.733021
x,y,z [Debye]:    -5.078445    -0.251185     1.863192
```

Table S5. Mulliken Charges for **3f**

```
-----------------------
MULLIKEN ATOMIC CHARGES
-----------------------
   0 S :   -0.030390
   1 S :   -0.037355
   2 S :   -0.010330
   3 S :   -0.037543
   4 S :   -0.035342
   5 O :   -0.330306
   6 O :   -0.320456
   7 O :   -0.304417
   8 N :    0.043297
   9 N :    0.418031
  10 C :   -0.290881
  11 H :    0.113240
  12 H :    0.114096
  13 H :    0.109590
  14 C :   -0.008707
  15 H :    0.107906
  16 H :    0.099927
```

17 C :   0.231450
18 C :   -0.032241
19 C :   -0.022416
20 C :   0.141567
21 C :   -0.218190
22 H :   0.123942
23 C :   -0.153833
24 H :   0.162161
25 C :   0.110293
26 C :   -0.126437
27 H :   0.183339
Sum of atomic charges: 0.0000000

Table S6. Summary of TD-DFT calculated first 20 low energy transitions of **3f**.

TD-DFT/TDA EXCITED STATES (SINGLETS)
----

the weight of the individual excitations are printed if larger than 1.0e-02

STATE 1: E=   0.097144 au   2.643 eV   21320.7 cm**-1 <S**2> =   0.000000
   92a -> 93a :   0.968611 (c= -0.98418029)

STATE 2: E=   0.126302 au   3.437 eV   27720.2 cm**-1 <S**2> =   0.000000
   87a -> 93a :   0.012088 (c= -0.10994337)
   88a -> 93a :   0.026520 (c= -0.16284891)
   91a -> 93a :   0.271837 (c= -0.52138021)
   92a -> 94a :   0.634348 (c= 0.79645969)

STATE 3: E=   0.134524 au   3.661 eV   29524.7 cm**-1 <S**2> =   0.000000
   87a -> 93a :   0.019860 (c= 0.14092474)
   88a -> 93a :   0.051426 (c= 0.22677365)
   91a -> 93a :   0.663955 (c= -0.81483406)
   91a -> 94a :   0.021844 (c= 0.14779691)
   92a -> 94a :   0.188617 (c= -0.43430049)
   92a -> 96a :   0.016694 (c= -0.12920673)

STATE 4: E=   0.135423 au   3.685 eV   29721.9 cm**-1 <S**2> =   0.000000
   89a -> 93a :   0.017568 (c= 0.13254474)
   90a -> 93a :   0.924296 (c= -0.96140334)
   90a -> 94a :   0.022996 (c= 0.15164453)

STATE 5: E=   0.138709 au   3.774 eV   30443.1 cm**-1 <S**2> =   0.000000
   86a -> 93a :   0.026293 (c= 0.16214985)
   89a -> 93a :   0.883192 (c= 0.93978306)
   89a -> 94a :   0.038872 (c= -0.19715904)
   90a -> 93a :   0.018846 (c= 0.13728160)

STATE 6: E=   0.141960 au   3.863 eV   31156.7 cm**-1 <S**2> =   0.000000
   86a -> 93a :   0.827233 (c= 0.90952359)
   86a -> 94a :   0.034834 (c= 0.18663853)
   86a -> 97a :   0.013376 (c= -0.11565563)
   88a -> 93a :   0.051354 (c= -0.22661427)
   89a -> 93a :   0.031760 (c= -0.17821442)

STATE 7: E=   0.145121 au   3.949 eV   31850.3 cm**-1 <S**2> =   0.000000
   86a -> 93a :   0.042246 (c= 0.20553798)
   87a -> 93a :   0.049429 (c= -0.22232571)

```
   88a -> 93a  :     0.441661 (c=  0.66457566)
   88a -> 94a  :     0.010090 (c= -0.10044855)
   90a -> 94a  :     0.011223 (c=  0.10593862)
   92a -> 95a  :     0.391606 (c= -0.62578464)

STATE  8:  E=   0.145886 au      3.970 eV    32018.3 cm**-1 <S**2> =   0.000000
   87a -> 93a  :     0.037200 (c= -0.19287226)
   88a -> 93a  :     0.181457 (c=  0.42597761)
   91a -> 94a  :     0.394083 (c= -0.62776022)
   91a -> 95a  :     0.012577 (c=  0.11214638)
   91a -> 96a  :     0.011237 (c= -0.10600649)
   92a -> 95a  :     0.243633 (c=  0.49359185)
   92a -> 96a  :     0.057016 (c=  0.23877942)
   92a -> 97a  :     0.011502 (c= -0.10724587)

STATE  9:  E=   0.146285 au      3.981 eV    32105.8 cm**-1 <S**2> =   0.000000
   88a -> 93a  :     0.073210 (c= -0.27057289)
   88a -> 94a  :     0.015495 (c=  0.12448056)
   90a -> 94a  :     0.019506 (c=  0.13966323)
   91a -> 93a  :     0.020112 (c= -0.14181527)
   91a -> 94a  :     0.475087 (c= -0.68926552)
   91a -> 96a  :     0.011144 (c= -0.10556597)
   92a -> 95a  :     0.233723 (c= -0.48344939)
   92a -> 96a  :     0.119410 (c= -0.34555769)

STATE 10:  E=   0.150561 au      4.097 eV    33044.3 cm**-1 <S**2> =   0.000000
   87a -> 93a  :     0.200292 (c= -0.44753946)
   88a -> 94a  :     0.014277 (c=  0.11948522)
   89a -> 94a  :     0.133718 (c= -0.36567520)
   90a -> 93a  :     0.011748 (c=  0.10838949)
   90a -> 94a  :     0.476963 (c=  0.69062475)
   91a -> 94a  :     0.018403 (c=  0.13565857)
   92a -> 95a  :     0.031622 (c=  0.17782503)
   92a -> 96a  :     0.045715 (c= -0.21381125)

STATE 11:  E=   0.151728 au      4.129 eV    33300.5 cm**-1 <S**2> =   0.000000
   87a -> 93a  :     0.204013 (c= -0.45167766)
   88a -> 94a  :     0.058726 (c= -0.24233382)
   90a -> 94a  :     0.243910 (c= -0.49387260)
   92a -> 96a  :     0.415922 (c= -0.64492041)

STATE 12:  E=   0.154654 au      4.208 eV    33942.6 cm**-1 <S**2> =   0.000000
   88a -> 94a  :     0.012829 (c= -0.11326732)
   89a -> 93a  :     0.031381 (c= -0.17714575)
   89a -> 94a  :     0.666695 (c= -0.81651404)
   89a -> 96a  :     0.017317 (c= -0.13159301)
   90a -> 94a  :     0.083362 (c= -0.28872414)
   92a -> 95a  :     0.018144 (c= -0.13470141)
   92a -> 96a  :     0.118691 (c=  0.34451523)

STATE 13:  E=   0.158065 au      4.301 eV    34691.2 cm**-1 <S**2> =   0.000000
   87a -> 93a  :     0.194462 (c= -0.44097836)
   88a -> 94a  :     0.388004 (c=  0.62289998)
   90a -> 94a  :     0.043529 (c= -0.20863523)
   92a -> 96a  :     0.020061 (c=  0.14163535)
   92a -> 97a  :     0.251567 (c=  0.50156476)

STATE 14:  E=   0.162339 au      4.417 eV    35629.3 cm**-1 <S**2> =   0.000000
```

```
   87a -> 93a  :     0.187833 (c=  0.43339751)
   88a -> 93a  :     0.117909 (c=  0.34337850)
   88a -> 94a  :     0.261793 (c=  0.51165743)
   88a -> 96a  :     0.012618 (c=  0.11232854)
   89a -> 94a  :     0.056195 (c= -0.23705487)
   91a -> 93a  :     0.015760 (c=  0.12554076)
   91a -> 95a  :     0.035626 (c= -0.18874980)
   92a -> 94a  :     0.055445 (c=  0.23546751)
   92a -> 95a  :     0.017504 (c=  0.13230119)
   92a -> 96a  :     0.106780 (c= -0.32677150)
   92a -> 99a  :     0.014574 (c= -0.12072170)

STATE 15:  E=   0.162846 au      4.431 eV    35740.6 cm**-1 <S**2> =   0.000000
   82a -> 93a  :     0.014237 (c=  0.11931849)
   83a -> 93a  :     0.069552 (c= -0.26372757)
   87a -> 93a  :     0.030197 (c=  0.17377193)
   87a -> 94a  :     0.245618 (c=  0.49559834)
   88a -> 94a  :     0.055027 (c= -0.23457764)
   90a -> 94a  :     0.015044 (c=  0.12265295)
   90a -> 96a  :     0.013306 (c=  0.11535027)
   91a -> 95a  :     0.041256 (c=  0.20311605)
   92a -> 96a  :     0.024762 (c= -0.15736059)
   92a -> 97a  :     0.394685 (c=  0.62823959)
   92a -> 98a  :     0.011397 (c=  0.10675661)

STATE 16:  E=   0.164622 au      4.480 eV    36130.4 cm**-1 <S**2> =   0.000000
   82a -> 93a  :     0.155132 (c= -0.39386737)
   83a -> 93a  :     0.648993 (c=  0.80560083)
   83a -> 94a  :     0.024409 (c=  0.15623531)
   84a -> 93a  :     0.034276 (c=  0.18513899)
   87a -> 93a  :     0.031491 (c=  0.17745723)
   87a -> 94a  :     0.012713 (c=  0.11275371)
   92a -> 97a  :     0.030932 (c=  0.17587608)

STATE 17:  E=   0.168206 au      4.577 eV    36917.0 cm**-1 <S**2> =   0.000000
   88a -> 94a  :     0.066465 (c= -0.25780863)
   91a -> 95a  :     0.571494 (c= -0.75597189)
   91a -> 96a  :     0.087877 (c= -0.29644082)
   92a -> 97a  :     0.064177 (c=  0.25333173)
   92a -> 98a  :     0.117355 (c= -0.34257123)

STATE 18:  E=   0.170529 au      4.640 eV    37426.7 cm**-1 <S**2> =   0.000000
   87a -> 94a  :     0.171637 (c= -0.41429050)
   91a -> 95a  :     0.163662 (c=  0.40455161)
   91a -> 96a  :     0.014864 (c=  0.12191920)
   92a -> 97a  :     0.079413 (c=  0.28180269)
   92a -> 98a  :     0.514816 (c= -0.71750672)

STATE 19:  E=   0.172859 au      4.704 eV    37938.2 cm**-1 <S**2> =   0.000000
   87a -> 94a  :     0.338113 (c= -0.58147451)
   88a -> 94a  :     0.012169 (c= -0.11031413)
   89a -> 95a  :     0.068988 (c= -0.26265551)
   90a -> 95a  :     0.105392 (c=  0.32464146)
   91a -> 96a  :     0.037307 (c= -0.19315107)
   91a -> 98a  :     0.015873 (c=  0.12598955)
   92a -> 97a  :     0.060957 (c=  0.24689564)
   92a -> 98a  :     0.238424 (c=  0.48828686)
   92a -> 99a  :     0.029528 (c= -0.17183763)
```

STATE 20: E= 0.173594 au 4.724 eV 38099.4 cm**-1 <S**2> = 0.000000
87a -> 94a : 0.028483 (c= 0.16876922)
88a -> 95a : 0.017288 (c= -0.13148453)
89a -> 95a : 0.387250 (c= -0.62229422)
89a -> 96a : 0.070777 (c= -0.26604032)
89a -> 97a : 0.011095 (c= -0.10533331)
90a -> 95a : 0.258719 (c= -0.50864396)
90a -> 96a : 0.021689 (c= -0.14727264)
91a -> 95a : 0.017112 (c= 0.13081416)
91a -> 96a : 0.038442 (c= -0.19606620)
91a -> 97a : 0.036550 (c= -0.19118076)
91a -> 98a : 0.058625 (c= 0.24212528)

ABSORPTION SPECTRUM VIA TRANSITION ELECTRIC DIPOLE MOMENTS

State	Energy (cm-1)	Wavelength (nm)	fosc	T2 (au**2)	TX (au)	TY (au)	TZ (au)
1	21320.7	469.0	0.010835612	0.16731	-0.03415	0.40052	0.07567
2	27720.2	360.7	0.051481724	0.61141	-0.56175	-0.26025	-0.47761
3	29524.7	338.7	0.152175870	1.69682	0.88829	0.54255	0.78321
4	29721.9	336.5	0.009504681	0.10528	-0.16325	-0.21205	-0.18347
5	30443.1	328.5	0.002708001	0.02928	0.08953	0.14051	0.03907
6	31156.7	321.0	0.012993223	0.13729	0.25554	0.17346	0.20470
7	31850.3	314.0	0.058048021	0.60000	-0.58439	-0.22272	-0.45703
8	32018.3	312.3	0.019938248	0.20501	-0.10933	-0.40129	-0.17893
9	32105.8	311.5	0.015326064	0.15715	0.20162	0.28260	0.19142
10	33044.3	302.6	0.021342589	0.21263	0.39438	-0.10025	0.21689
11	33300.5	300.3	0.000025238	0.00025	-0.00986	0.00870	-0.00876
12	33942.6	294.6	0.008515699	0.08259	0.06969	0.27804	0.02072
13	34691.2	288.3	0.055508883	0.52677	0.40611	0.47891	0.36399
14	35629.3	280.7	0.443776723	4.10046	-1.38567	-1.02201	-1.06577
15	35740.6	279.8	0.036708334	0.33813	-0.27075	-0.40468	-0.31789
16	36130.4	276.8	0.018837733	0.17164	-0.20917	-0.21397	-0.28655
17	36917.0	270.9	0.017617502	0.15711	0.30294	-0.21385	-0.14000
18	37426.7	267.2	0.017719290	0.15586	-0.32326	-0.18005	-0.13766
19	37938.2	263.6	0.057886654	0.50232	-0.38886	-0.41462	-0.42331
20	38099.4	262.5	0.007297595	0.06306	-0.08397	0.23097	-0.05156

ABSORPTION SPECTRUM VIA TRANSITION VELOCITY DIPOLE MOMENTS

State	Energy (cm-1)	Wavelength (nm)	fosc	P2 (au**2)	PX (au)	PY (au)	PZ (au)
1	21320.7	469.0	0.019996950	0.00291	-0.04393	0.02751	-0.01509
2	27720.2	360.7	0.001552818	0.00029	-0.00777	0.00673	0.01373
3	29524.7	338.7	0.008998225	0.00182	-0.02212	-0.01543	-0.03299
4	29721.9	336.5	0.002709653	0.00055	0.00075	-0.00119	0.02342
5	30443.1	328.5	0.006275529	0.00131	-0.02995	-0.02000	-0.00300
6	31156.7	321.0	0.000990041	0.00021	-0.01178	-0.00530	-0.00664
7	31850.3	314.0	0.012236281	0.00266	0.03207	-0.00068	0.04043
8	32018.3	312.3	0.003337369	0.00073	0.02250	0.00690	0.01329
9	32105.8	311.5	0.001812816	0.00040	-0.00114	-0.01834	-0.00775
10	33044.3	302.6	0.007436346	0.00168	-0.03648	-0.00397	-0.01824
11	33300.5	300.3	0.005211835	0.00119	-0.03315	-0.00139	0.00923

12	33942.6	294.6	0.008660567	0.00201	-0.03053	-0.03170	-0.00849
13	34691.2	288.3	0.004678621	0.00111	-0.00793	-0.02260	-0.02314
14	35629.3	280.7	0.068885915	0.01677	0.07687	0.06843	0.07863
15	35740.6	279.8	0.000865539	0.00021	0.01145	0.00656	0.00611
16	36130.4	276.8	0.015274224	0.00377	-0.00448	-0.00645	0.06091
17	36917.0	270.9	0.018394657	0.00464	-0.05040	-0.01520	0.04325
18	37426.7	267.2	0.007131332	0.00182	0.03712	0.01488	0.01499
19	37938.2	263.6	0.004247779	0.00110	0.02019	0.01118	0.02385
20	38099.4	262.5	0.002419075	0.00063	0.01765	-0.01532	0.00916

Table S7. Electric properties calculations of **3f**.

The origin for moment calculation is the CENTER OF MASS = (10.368261, 9.432489 5.319525)

DIPOLE MOMENT

	X	Y	Z
Electronic contribution:	16.10917	-7.61403	4.96568
Nuclear contribution :	-16.15544	8.40189	-4.46986
Total Dipole Moment :	-0.04627	0.78786	0.49582

Magnitude (a.u.) : 0.93204
Magnitude (Debye) : 2.36905

Rotational spectrum

Rotational constants in cm-1: 0.012346 0.005076 0.003843
Rotational constants in MHz : 370.110005 152.166380 115.210831

Dipole components along the rotational axes:
x,y,z [a.u.] : 0.613008 0.681169 0.170074
x,y,z [Debye]: 1.558143 1.731393 0.432294

Table S8. Crystal data and structure refinement for 6-ethoxy-[1,2,3,4,5]pentathiepino[6,7-a]indolizine (**3a**).

Identification code	SIV-01-70
Empirical formula	$C_{10}H_9NS_5O$
Formula weight	319.48
Temperature	170(2) K
Wavelength	0.71073 A
Crystal system, space group	Orthorombic, P b c a
Unit cell dimensions	a = 8.6214(17) A alpha = 90 deg.
	b = 16.866(3) A beta = 90 deg.
	c = 17.750(4) A gamma = 90 deg.
Volume	2581.1(9) A^3
Z, calculated value	8, 1.644 Mg/m^3
Absorption coefficient	0.878 mm^-1
F(000)	1312
Crystal size	0.212 x 0.175 x 0.167 mm
Theta range for data collection	3.294 to 26.766 deg.
Limiting indices	-10<=h<=10, -21<=k<=21, -19<=l<=22
Reflections collected / unique	20574 / 2723 [R(int) = 0.0499]
Completeness to theta =	99.8 %
Absorption correction	Numerical
Max. and min. transmission	0.9933 and 0.8297
Refinement method	Full-matrix least-squares on F^2
Data / restraints / parameters	2723 / 0 / 155
Goodness-of-fit on F^2	1.032
Final R indices [I>2sigma(I)]	R1 = 0.0314, wR2 = 0.0789
R indices (all data)	R1 = 0.0430, wR2 = 0.0823
Extinction coefficient	n/a
Largest diff. peak and hole	1.027 and -0.610 e.A^-3

Table 2. Atomic coordinates (x 10^4) and equivalent isotropic displacement parameters (A^2 x 10^3) for siv0170.
U(eq) is defined as one third of the trace of the orthogonalize Uij tensor.

	x	y	z	U(eq)
S(1)	7858(1)	6719(1)	4190(1)	31(1)
S(2)	6166(1)	7347(1)	4753(1)	35(1)
S(3)	5142(1)	8045(1)	3941(1)	35(1)
S(4)	3719(1)	7293(1)	3355(1)	36(1)
S(5)	5192(1)	6649(1)	2669(1)	30(1)
O(1)	7901(2)	4807(1)	4661(1)	29(1)
N(1)	6064(2)	4640(1)	3703(1)	23(1)
C(1)	7004(2)	5129(1)	4125(1)	24(1)
C(2)	6862(2)	5891(1)	3850(1)	24(1)
C(3)	5781(2)	5871(1)	3238(1)	25(1)
C(4)	5284(2)	5092(1)	3158(1)	23(1)
C(5)	4191(3)	4698(1)	2692(1)	28(1)
C(6)	3933(3)	3908(1)	2790(1)	31(1)
C(7)	4766(3)	3476(1)	3345(1)	30(1)
C(8)	5814(3)	3836(1)	3792(1)	27(1)
C(9)	7365(4)	4889(2)	5417(2)	56(1)
C(10)	8525(3)	4543(2)	5939(1)	37(1)

Table 3. Bond lengths [A] and angles [deg] for siv0170.

S(1)-C(2)	1.748(2)
S(1)-S(2)	2.0613(9)
S(2)-S(3)	2.0599(9)
S(3)-S(4)	2.0483(9)
S(4)-S(5)	2.0672(9)
S(5)-C(3)	1.732(2)
O(1)-C(1)	1.341(3)
O(1)-C(9)	1.427(3)
N(1)-C(1)	1.378(3)
N(1)-C(8)	1.382(3)
N(1)-C(4)	1.404(3)
C(1)-C(2)	1.379(3)
C(2)-C(3)	1.431(3)
C(3)-C(4)	1.390(3)
C(4)-C(5)	1.419(3)
C(5)-C(6)	1.361(3)
C(5)-H(5)	0.9500
C(6)-C(7)	1.421(3)
C(6)-H(6)	0.9500
C(7)-C(8)	1.347(3)
C(7)-H(7)	0.9500
C(8)-H(8)	0.9500
C(9)-C(10)	1.483(4)
C(9)-H(9A)	0.9900
C(9)-H(9B)	0.9900
C(10)-H(10A)	0.9800
C(10)-H(10B)	0.9800
C(10)-H(10C)	0.9800
C(2)-S(1)-S(2)	103.29(8)
S(3)-S(2)-S(1)	104.91(4)
S(4)-S(3)-S(2)	104.93(4)
S(3)-S(4)-S(5)	104.86(4)
C(3)-S(5)-S(4)	103.57(8)
C(1)-O(1)-C(9)	116.18(18)
C(1)-N(1)-C(8)	128.15(18)
C(1)-N(1)-C(4)	109.30(16)
C(8)-N(1)-C(4)	122.49(18)
O(1)-C(1)-N(1)	118.75(18)
O(1)-C(1)-C(2)	132.82(19)
N(1)-C(1)-C(2)	108.30(18)
C(1)-C(2)-C(3)	107.74(18)
C(1)-C(2)-S(1)	125.39(17)
C(3)-C(2)-S(1)	126.87(16)
C(4)-C(3)-C(2)	107.48(18)
C(4)-C(3)-S(5)	124.47(17)
C(2)-C(3)-S(5)	128.03(16)
C(3)-C(4)-N(1)	107.18(18)
C(3)-C(4)-C(5)	135.0(2)
N(1)-C(4)-C(5)	117.74(18)
C(6)-C(5)-C(4)	119.4(2)
C(6)-C(5)-H(5)	120.3
C(4)-C(5)-H(5)	120.3
C(5)-C(6)-C(7)	120.6(2)
C(5)-C(6)-H(6)	119.7

C(7)-C(6)-H(6)	119.7
C(8)-C(7)-C(6)	121.0(2)
C(8)-C(7)-H(7)	119.5
C(6)-C(7)-H(7)	119.5
C(7)-C(8)-N(1)	118.7(2)
C(7)-C(8)-H(8)	120.7
N(1)-C(8)-H(8)	120.7
O(1)-C(9)-C(10)	109.3(2)
O(1)-C(9)-H(9A)	109.8
C(10)-C(9)-H(9A)	109.8
O(1)-C(9)-H(9B)	109.8
C(10)-C(9)-H(9B)	109.8
H(9A)-C(9)-H(9B)	108.3
C(9)-C(10)-H(10A)	109.5
C(9)-C(10)-H(10B)	109.5
H(10A)-C(10)-H(10B)	109.5
C(9)-C(10)-H(10C)	109.5
H(10A)-C(10)-H(10C)	109.5
H(10B)-C(10)-H(10C)	109.5

Symmetry transformations used to generate equivalent atoms:

Table 4. Anisotropic displacement parameters (A^2 x 10^3) for siv0170.
The anisotropic displacement factor exponent takes the form:
-2 pi^2 [h^2 a*^2 U11 + ... + 2 h k a* b* U12]

	U11	U22	U33	U23	U13	U12
S(1)	29(1)	26(1)	37(1)	-2(1)	-4(1)	-3(1)
S(2)	48(1)	31(1)	27(1)	-2(1)	3(1)	0(1)
S(3)	43(1)	26(1)	38(1)	-1(1)	4(1)	4(1)
S(4)	31(1)	31(1)	45(1)	1(1)	0(1)	6(1)
S(5)	39(1)	26(1)	25(1)	5(1)	-1(1)	3(1)
O(1)	32(1)	32(1)	24(1)	1(1)	-4(1)	8(1)
N(1)	25(1)	21(1)	23(1)	2(1)	1(1)	0(1)
C(1)	22(1)	26(1)	23(1)	0(1)	0(1)	2(1)
C(2)	25(1)	23(1)	25(1)	0(1)	1(1)	-2(1)
C(3)	26(1)	24(1)	24(1)	2(1)	1(1)	1(1)
C(4)	23(1)	26(1)	21(1)	0(1)	2(1)	2(1)
C(5)	29(1)	32(1)	22(1)	-1(1)	-1(1)	-1(1)
C(6)	33(1)	32(1)	28(1)	-6(1)	0(1)	-6(1)
C(7)	36(1)	23(1)	33(1)	-2(1)	5(1)	-3(1)
C(8)	31(1)	21(1)	29(1)	1(1)	5(1)	2(1)
C(9)	68(2)	73(2)	28(1)	0(1)	1(1)	36(2)
C(10)	33(1)	51(2)	27(1)	6(1)	0(1)	6(1)

Table 5. Hydrogen bonds for siv0170 [A and deg.].

Nr	Typ	Res	Donor --- H....Acceptor	[ARU]	D - H	H...A	D...A	D - H...A
1	1		C(10) --H(10C) ..O(1)	[5555.01]	0.98	2.54	3.439(3)	152

Translation of ARU-Code to CIF and Equivalent Position Code
==
[5555.] = -x,-y,-z

Table S9. Crystal data and structure refinement for 6-ethoxy-9-fluoro-[1,2,3,4,5]pentathiepino[6,7-a]indolizine (**3b**).

Identification code	RT105
Empirical formula	$C_{10}H_8NS_5OF$
Formula weight	337.47
Temperature	170(2) K
Wavelength	0.71073 A
Crystal system, space group	Triclinic, P -1
Unit cell dimensions	a = 8.4433(17) A alpha = 96.08(3) deg.
	b = 8.5239(17) A beta = 96.15(3) deg.
	c = 9.4614(19) A gamma = 99.51(3) deg.
Volume	662.4(2) A^3
Z, calculated value	2, 1.692 Mg/m^3
Absorption coefficient	0.871 mm^-1
F(000)	344
Crystal size	0.137 x 0.135 x 0.092 mm
Theta range for data collection	2.182 to 29.471 deg.
Limiting indices	-11<=h<=11, -11<=k<=11, -13<=l<=11
Reflections collected / unique	7509 / 3639 [R(int) = 0.0289]
Completeness to theta =	25.242 99.7 %
Absorption correction	Numerical
Max. and min. transmission	0.9534 and 0.8334
Refinement method	Full-matrix least-squares on F^2
Data / restraints / parameters	3639 / 0 / 164
Goodness-of-fit on F^2	1.052
Final R indices [I>2sigma(I)]	R1 = 0.0359, wR2 = 0.0844

R indices (all data)	R1 = 0.0535, wR2 = 0.0942
Extinction coefficient	n/a
Largest diff. peak and hole	0.411 and -0.399 e.A^-3

Atomic coordinates (x 10^4) and equivalent isotropic

displacement parameters (A^2 x 10^3) for RT105.

U(eq) is defined as one third of the trace of the orthogonalized

Uij tensor.

	x	y	z	U(eq)
S(1)	2250(1)	4047(1)	2532(1)	34(1)
S(2)	2424(1)	4473(1)	4733(1)	39(1)
S(3)	1878(1)	6717(1)	5123(1)	40(1)
S(4)	3965(1)	8224(1)	4890(1)	35(1)
S(5)	3959(1)	8161(1)	2708(1)	31(1)
F(1)	9200(2)	2479(2)	162(2)	41(1)
O(1)	7511(2)	7853(2)	1663(2)	31(1)
N(1)	6642(2)	5067(2)	1482(2)	26(1)
C(1)	7974(3)	4577(3)	967(2)	30(1)
C(2)	7925(3)	2995(3)	694(2)	32(1)
C(3)	6619(3)	1825(3)	925(2)	35(1)
C(4)	5316(3)	2322(3)	1426(2)	31(1)
C(5)	5285(3)	3979(2)	1707(2)	27(1)
C(6)	4166(3)	4867(3)	2178(2)	28(1)
C(7)	4860(3)	6513(2)	2250(2)	26(1)
C(8)	6384(3)	6600(2)	1829(2)	27(1)
C(9)	8544(4)	8558(4)	2955(3)	63(1)
C(10)	9823(3)	9840(3)	2682(3)	41(1)

Bond lengths [A] and angles [deg] for RT105.

S(1)-C(6)	1.735(2)
S(1)-S(2)	2.0609(9)
S(2)-S(3)	2.0462(11)
S(3)-S(4)	2.0485(11)
S(4)-S(5)	2.0596(9)
S(5)-C(7)	1.742(2)
F(1)-C(2)	1.354(3)
O(1)-C(8)	1.342(2)
O(1)-C(9)	1.435(3)
N(1)-C(8)	1.372(3)
N(1)-C(1)	1.380(3)
N(1)-C(5)	1.401(3)
C(1)-C(2)	1.338(3)
C(2)-C(3)	1.412(3)
C(3)-C(4)	1.355(3)
C(4)-C(5)	1.415(3)
C(5)-C(6)	1.386(3)
C(6)-C(7)	1.419(3)
C(7)-C(8)	1.380(3)
C(9)-C(10)	1.468(3)
C(6)-S(1)-S(2)	103.25(8)
S(3)-S(2)-S(1)	104.23(5)
S(2)-S(3)-S(4)	104.05(4)
S(3)-S(4)-S(5)	104.62(5)
C(7)-S(5)-S(4)	103.36(8)
C(8)-O(1)-C(9)	114.29(18)
C(8)-N(1)-C(1)	128.47(18)
C(8)-N(1)-C(5)	109.11(18)
C(1)-N(1)-C(5)	122.41(18)
C(2)-C(1)-N(1)	117.0(2)
C(1)-C(2)-F(1)	118.3(2)
C(1)-C(2)-C(3)	124.0(2)
F(1)-C(2)-C(3)	117.7(2)
C(4)-C(3)-C(2)	118.4(2)
C(3)-C(4)-C(5)	120.1(2)
C(6)-C(5)-N(1)	107.32(18)

C(6)-C(5)-C(4)	134.6(2)
N(1)-C(5)-C(4)	118.0(2)
C(5)-C(6)-C(7)	107.51(19)
C(5)-C(6)-S(1)	124.42(16)
C(7)-C(6)-S(1)	128.03(18)
C(8)-C(7)-C(6)	107.82(19)
C(8)-C(7)-S(5)	124.80(16)
C(6)-C(7)-S(5)	127.33(17)
O(1)-C(8)-N(1)	119.85(19)
O(1)-C(8)-C(7)	131.8(2)
N(1)-C(8)-C(7)	108.23(18)
O(1)-C(9)-C(10)	111.6(2)

Symmetry transformations used to generate equivalent atoms:

Anisotropic displacement parameters (A^2 x 10^3) for RT105.

The anisotropic displacement factor exponent takes the form:

-2 pi^2 [h^2 a*^2 U11 + ... + 2 h k a* b* U12]

	U11	U22	U33	U23	U13	U12
S(1)	27(1)	36(1)	35(1)	4(1)	1(1)	-5(1)
S(2)	38(1)	44(1)	35(1)	14(1)	7(1)	-3(1)
S(3)	32(1)	51(1)	36(1)	5(1)	11(1)	2(1)
S(4)	36(1)	38(1)	28(1)	-1(1)	6(1)	1(1)
S(5)	36(1)	28(1)	30(1)	5(1)	6(1)	8(1)
F(1)	39(1)	43(1)	42(1)	-2(1)	5(1)	16(1)
O(1)	33(1)	27(1)	30(1)	6(1)	6(1)	-4(1)
N(1)	28(1)	25(1)	24(1)	3(1)	3(1)	2(1)
C(1)	30(1)	32(1)	26(1)	4(1)	3(1)	5(1)
C(2)	33(1)	36(1)	28(1)	1(1)	0(1)	10(1)
C(3)	43(1)	28(1)	31(1)	0(1)	-4(1)	8(1)
C(4)	36(1)	25(1)	29(1)	5(1)	-2(1)	0(1)
C(5)	29(1)	26(1)	23(1)	3(1)	-1(1)	0(1)
C(6)	28(1)	27(1)	26(1)	5(1)	1(1)	0(1)
C(7)	30(1)	25(1)	24(1)	4(1)	2(1)	3(1)
C(8)	30(1)	24(1)	25(1)	5(1)	4(1)	1(1)
C(9)	66(2)	65(2)	39(2)	13(1)	-9(1)	-34(2)
C(10)	37(1)	34(1)	48(1)	5(1)	2(1)	-2(1)

Table 5. Torsion angles [deg] for RT105.

C(8)-N(1)-C(1)-C(2)	-179.3(2)
C(5)-N(1)-C(1)-C(2)	-0.5(3)
N(1)-C(1)-C(2)-F(1)	178.73(18)
N(1)-C(1)-C(2)-C(3)	-1.1(3)
C(1)-C(2)-C(3)-C(4)	1.4(3)
F(1)-C(2)-C(3)-C(4)	-178.46(19)
C(2)-C(3)-C(4)-C(5)	-0.1(3)
C(8)-N(1)-C(5)-C(6)	1.0(2)
C(1)-N(1)-C(5)-C(6)	-178.06(18)
C(8)-N(1)-C(5)-C(4)	-179.27(18)
C(1)-N(1)-C(5)-C(4)	1.7(3)
C(3)-C(4)-C(5)-C(6)	178.3(2)
C(3)-C(4)-C(5)-N(1)	-1.4(3)
N(1)-C(5)-C(6)-C(7)	-0.5(2)
C(4)-C(5)-C(6)-C(7)	179.9(2)
N(1)-C(5)-C(6)-S(1)	177.34(14)
C(4)-C(5)-C(6)-S(1)	-2.3(4)
S(2)-S(1)-C(6)-C(5)	109.15(18)
S(2)-S(1)-C(6)-C(7)	-73.5(2)
C(5)-C(6)-C(7)-C(8)	-0.2(2)
S(1)-C(6)-C(7)-C(8)	-177.92(16)
C(5)-C(6)-C(7)-S(5)	177.28(15)
S(1)-C(6)-C(7)-S(5)	-0.4(3)
S(4)-S(5)-C(7)-C(8)	-109.22(18)
S(4)-S(5)-C(7)-C(6)	73.70(19)
C(9)-O(1)-C(8)-N(1)	-98.6(3)
C(9)-O(1)-C(8)-C(7)	85.0(3)
C(1)-N(1)-C(8)-O(1)	0.7(3)
C(5)-N(1)-C(8)-O(1)	-178.25(17)
C(1)-N(1)-C(8)-C(7)	177.85(19)
C(5)-N(1)-C(8)-C(7)	-1.1(2)
C(6)-C(7)-C(8)-O(1)	177.5(2)
S(5)-C(7)-C(8)-O(1)	-0.1(3)
C(6)-C(7)-C(8)-N(1)	0.8(2)
S(5)-C(7)-C(8)-N(1)	-176.74(14)
C(8)-O(1)-C(9)-C(10)	175.7(2)

Table S10. Crystal data and structure refinement for 6-ethoxy-9-chloro-[1,2,3,4,5]pentathiepino[6,7-a]indolizine (**3c**).

Identification code	RT143
Empirical formula	$C_{10}H_8NClS_5O$
Formula weight	353.92
Temperature	100(2) K
Wavelength	1.54184 A
Crystal system, space group	Orthorhombic, P c a 21
Unit cell dimensions	a = 19.22720(10) A alpha = 90 deg.
	b = 8.61740(10) A beta = 90 deg.
	c = 8.20880(10) A gamma = 90 deg.
Volume	1360.10(2) A^3
Z, calculated value	4, 1.728 Mg/m^3
Absorption coefficient	9.550 mm^-1
F(000)	720
Crystal size	0.205 x 0.090 x 0.063 mm
Theta range for data collection	4.600 to 80.026 deg.
Limiting indices	-24<=h<=24, -11<=k<=10, -10<=l<=10
Reflections collected / unique	47449 / 2962 [R(int) = 0.0581]
Completeness to theta =	0.0 %
Absorption correction	Numerical
Max. and min. transmission	1.07 and 0.45
Refinement method	Full-matrix least-squares on F^2
Data / restraints / parameters	2962 / 1 / 164
Goodness-of-fit on F^2	1.065
Final R indices [I>2sigma(I)]	R1 = 0.0241, wR2 = 0.0657
R indices (all data)	R1 = 0.0243, wR2 = 0.0663
Absolute structure parameter	-0.002(9)
Extinction coefficient	n/a
Largest diff. peak and hole	0.352 and -0.276 e.A^-3

Table 2. Atomic coordinates (x 10^4) and equivalent isotropic displacement parameters (A^2 x 10^3) for rt143.
U(eq) is defined as one third of the trace of the orthogonalized Uij tensor.

	x	y	z	U(eq)
Cl(1)	7905(1)	9185(1)	3142(1)	23(1)
S(1)	4828(1)	4585(1)	4629(1)	17(1)
S(2)	4741(1)	2687(1)	3096(1)	19(1)
S(3)	4742(1)	803(1)	4657(1)	20(1)
S(4)	5755(1)	543(1)	5417(1)	19(1)
S(5)	5892(1)	2238(1)	7171(1)	16(1)
O(1)	7406(1)	3991(2)	6689(3)	18(1)
N(1)	6810(1)	5717(3)	4950(3)	16(1)
C(1)	5719(1)	4771(3)	4989(3)	15(1)
C(2)	6146(2)	3837(3)	6007(4)	16(1)
C(3)	6813(2)	4435(3)	5955(4)	16(1)
C(4)	6140(1)	5930(3)	4316(4)	15(1)
C(5)	6037(2)	7163(3)	3205(4)	16(1)
C(6)	6574(2)	8125(3)	2822(4)	18(1)
C(7)	7239(2)	7887(4)	3565(4)	17(1)
C(8)	7360(1)	6694(3)	4597(4)	18(1)
C(9)	7777(2)	2791(4)	5762(4)	20(1)
C(10)	8461(2)	2538(4)	6604(4)	22(1)

Table 3. Bond lengths [A] and angles [deg] for rt143.

Cl(1)-C(7)	1.736(3)
S(1)-C(1)	1.746(3)
S(1)-S(2)	2.0711(10)
S(2)-S(3)	2.0683(10)
S(3)-S(4)	2.0568(11)
S(4)-S(5)	2.0676(10)
S(5)-C(2)	1.747(3)
O(1)-C(3)	1.347(4)
O(1)-C(9)	1.469(4)
N(1)-C(3)	1.378(4)
N(1)-C(8)	1.383(4)
N(1)-C(4)	1.401(4)
C(1)-C(4)	1.400(4)
C(1)-C(2)	1.421(4)
C(2)-C(3)	1.383(4)
C(4)-C(5)	1.414(4)
C(5)-C(6)	1.361(4)
C(6)-C(7)	1.432(4)
C(7)-C(8)	1.352(5)
C(9)-C(10)	1.501(4)
C(1)-S(1)-S(2)	104.72(10)
S(3)-S(2)-S(1)	104.10(4)
S(4)-S(3)-S(2)	105.94(4)
S(3)-S(4)-S(5)	104.75(4)
C(2)-S(5)-S(4)	102.23(11)
C(3)-O(1)-C(9)	112.3(2)
C(3)-N(1)-C(8)	127.7(3)
C(3)-N(1)-C(4)	109.3(2)
C(8)-N(1)-C(4)	123.0(3)
C(4)-C(1)-C(2)	107.5(2)
C(4)-C(1)-S(1)	124.5(2)
C(2)-C(1)-S(1)	127.9(2)
C(3)-C(2)-C(1)	107.9(3)
C(3)-C(2)-S(5)	124.8(2)
C(1)-C(2)-S(5)	127.4(2)
O(1)-C(3)-N(1)	119.9(3)
O(1)-C(3)-C(2)	131.8(3)
N(1)-C(3)-C(2)	108.3(3)
C(1)-C(4)-N(1)	107.0(3)
C(1)-C(4)-C(5)	135.2(3)
N(1)-C(4)-C(5)	117.9(3)
C(6)-C(5)-C(4)	120.0(3)
C(5)-C(6)-C(7)	119.5(3)
C(8)-C(7)-C(6)	122.0(3)
C(8)-C(7)-Cl(1)	119.2(2)
C(6)-C(7)-Cl(1)	118.8(2)
C(7)-C(8)-N(1)	117.6(3)
O(1)-C(9)-C(10)	106.8(2)

Symmetry transformations used to generate equivalent atoms:

Table 4. Anisotropic displacement parameters (A^2 x 10^3) for rt143.
The anisotropic displacement factor exponent takes the form:
-2 pi^2 [h^2 a*^2 U11 + ... + 2 h k a* b* U12]

	U11	U22	U33	U23	U13	U12
Cl(1)	19(1)	25(1)	26(1)	4(1)	1(1)	-7(1)
S(1)	12(1)	19(1)	20(1)	1(1)	-1(1)	0(1)
S(2)	19(1)	22(1)	17(1)	1(1)	-3(1)	-4(1)
S(3)	21(1)	20(1)	18(1)	0(1)	0(1)	-5(1)
S(4)	21(1)	17(1)	17(1)	-1(1)	3(1)	1(1)
S(5)	16(1)	18(1)	14(1)	1(1)	1(1)	0(1)
O(1)	14(1)	23(1)	16(1)	0(1)	-4(1)	2(1)
N(1)	15(1)	16(1)	16(1)	0(1)	-1(1)	0(1)
C(1)	13(1)	16(1)	16(2)	-2(1)	1(1)	1(1)
C(2)	16(1)	18(1)	15(1)	-1(1)	0(1)	-1(1)
C(3)	15(1)	19(1)	13(1)	-1(1)	-1(1)	1(1)
C(4)	13(1)	19(1)	14(1)	-3(1)	-1(1)	1(1)
C(5)	15(1)	18(1)	16(1)	-3(1)	-2(1)	1(1)
C(6)	20(1)	17(1)	16(1)	1(1)	1(1)	2(1)
C(7)	15(1)	19(1)	18(1)	-3(1)	3(1)	-3(1)
C(8)	14(1)	21(1)	19(1)	-5(1)	-1(1)	-2(1)
C(9)	19(1)	24(2)	17(1)	-1(1)	-1(1)	3(1)
C(10)	18(2)	27(2)	22(2)	-1(1)	-3(1)	5(1)

Table 5. Torsion angles [deg] for rt143.

S(2)-S(1)-C(1)-C(4)	106.5(2)
S(2)-S(1)-C(1)-C(2)	-74.8(3)
C(4)-C(1)-C(2)-C(3)	0.5(3)
S(1)-C(1)-C(2)-C(3)	-178.4(2)
C(4)-C(1)-C(2)-S(5)	179.1(2)
S(1)-C(1)-C(2)-S(5)	0.3(4)
S(4)-S(5)-C(2)-C(3)	-107.3(3)
S(4)-S(5)-C(2)-C(1)	74.2(3)
C(9)-O(1)-C(3)-N(1)	-94.8(3)
C(9)-O(1)-C(3)-C(2)	85.9(4)
C(8)-N(1)-C(3)-O(1)	-1.8(4)
C(4)-N(1)-C(3)-O(1)	179.1(3)
C(8)-N(1)-C(3)-C(2)	177.7(3)
C(4)-N(1)-C(3)-C(2)	-1.4(3)
C(1)-C(2)-C(3)-O(1)	180.0(3)
S(5)-C(2)-C(3)-O(1)	1.2(5)
C(1)-C(2)-C(3)-N(1)	0.6(3)
S(5)-C(2)-C(3)-N(1)	-178.2(2)
C(2)-C(1)-C(4)-N(1)	-1.3(3)
S(1)-C(1)-C(4)-N(1)	177.6(2)
C(2)-C(1)-C(4)-C(5)	178.1(3)
S(1)-C(1)-C(4)-C(5)	-3.1(5)
C(3)-N(1)-C(4)-C(1)	1.6(3)
C(8)-N(1)-C(4)-C(1)	-177.5(3)
C(3)-N(1)-C(4)-C(5)	-177.8(3)
C(8)-N(1)-C(4)-C(5)	3.0(4)
C(1)-C(4)-C(5)-C(6)	178.7(3)
N(1)-C(4)-C(5)-C(6)	-2.0(4)
C(4)-C(5)-C(6)-C(7)	-0.6(4)
C(5)-C(6)-C(7)-C(8)	2.5(5)
C(5)-C(6)-C(7)-Cl(1)	-176.7(2)
C(6)-C(7)-C(8)-N(1)	-1.5(5)
Cl(1)-C(7)-C(8)-N(1)	177.7(2)
C(3)-N(1)-C(8)-C(7)	179.8(3)
C(4)-N(1)-C(8)-C(7)	-1.3(4)
C(3)-O(1)-C(9)-C(10)	173.6(3)

Symmetry transformations used to generate equivalent atoms:

Table 6. Hydrogen bonds for rt143 [A and deg.].

D-H...A	d(D-H)	d(H...A)	d(D...A)	<(DHA)
C(9)-H(9A)...O(1)#1	0.99	2.57	3.518(4)	160.3
C(5)-H(5)...S(5)#2	0.95	2.92	3.839(3)	164.5
C(5)-H(5)...S(5)#2	0.95	2.92	3.839(3)	164.5
C(9)-H(9A)...O(1)#1	0.99	2.57	3.518(4)	160.3

Symmetry transformations used to generate equivalent atoms:
#1 -x+3/2,y,z-1/2 #2 -x+1,-y+1,z-1/2

Table S11. Crystal data and structure refinement for 6-ethoxy-9-bromo-[1,2,3,4,5]pentathiepino[6,7-a]indolizine (**3d**).

Identification code	FW-02-01
Empirical formula	$C_{10}H_8NBrS_5O$
Formula weight	398.38
Temperature	170(2) K
Wavelength	0.71073 A
Crystal system, space group	Orthorhombic, P c a 21
Unit cell dimensions	a = 19.380(4) A alpha = 90 deg.
	b = 8.7056(17) A beta = 90 deg.
	c = 8.2131(16) A gamma = 90 deg.
Volume	1385.6(5) A^3
Z, calculated value	4, 1.910 Mg/m^3
Absorption coefficient	3.704 mm^-1
F(000)	792
Crystal size	0.126 x 0.092 x 0.073 mm
Theta range for data collection	3.146 to 29.488 deg.
Limiting indices	-26<=h<=26, -12<=k<=11, -9<=l<=11
Reflections collected / unique	14351 / 3493 [R(int) = 0.0923]
Completeness to theta =	25.242 99.7 %
Absorption correction	Numerical
Max. and min. transmission	0.7978 and 0.6823
Refinement method	Full-matrix least-squares on F^2
Data / restraints / parameters	3493 / 1 / 165
Goodness-of-fit on F^2	0.996
Final R indices [I>2sigma(I)]	R1 = 0.0456, wR2 = 0.0913
R indices (all data)	R1 = 0.0932, wR2 = 0.1066
Absolute structure parameter	0.085(18)
Extinction coefficient	n/a
Largest diff. peak and hole	0.575 and -0.841 e.A^-3

Atomic coordinates (x 10^4) and equivalent isotropic

displacement parameters (A^2 x 10^3) for fw-02-01.

U(eq) is defined as one third of the trace of the orthogonalized

Uij tensor.

	x	y	z	U(eq)
Br(1)	7920(1)	9277(1)	3166(1)	38(1)
S(1)	5888(1)	2241(2)	7163(3)	29(1)
S(2)	5752(1)	574(2)	5403(3)	34(1)
S(3)	4751(1)	835(2)	4655(3)	35(1)
S(4)	4752(1)	2699(2)	3100(3)	35(1)
S(5)	4834(1)	4566(2)	4643(3)	32(1)
O(1)	7385(3)	4003(5)	6708(7)	32(1)
N(1)	6791(3)	5708(6)	4982(8)	28(1)
C(1)	8441(4)	2585(9)	6615(12)	40(2)
C(2)	7771(4)	2863(9)	5764(10)	34(2)
C(3)	6800(4)	4445(9)	5958(10)	29(2)
C(4)	6144(4)	3834(8)	6010(10)	30(2)
C(5)	5716(4)	4748(8)	4988(10)	27(2)
C(6)	6127(4)	5918(8)	4357(9)	27(2)
C(7)	6018(3)	7134(7)	3254(11)	30(2)
C(8)	6551(4)	8101(8)	2869(9)	29(2)
C(9)	7201(4)	7855(9)	3611(10)	33(2)
C(10)	7328(4)	6673(8)	4629(10)	29(2)

Bond lengths [A] and angles [deg] for fw-02-01.

Br(1)-C(9)	1.899(7)
S(1)-C(4)	1.751(8)
S(1)-S(2)	2.065(3)
S(2)-S(3)	2.047(3)
S(3)-S(4)	2.065(3)
S(4)-S(5)	2.067(3)
S(5)-C(5)	1.740(7)
O(1)-C(3)	1.347(9)
O(1)-C(2)	1.465(9)
N(1)-C(3)	1.361(9)
N(1)-C(10)	1.368(9)
N(1)-C(6)	1.399(9)
C(1)-C(2)	1.494(11)
C(3)-C(4)	1.378(11)
C(4)-C(5)	1.423(11)
C(5)-C(6)	1.392(10)
C(6)-C(7)	1.409(10)
C(7)-C(8)	1.369(10)
C(8)-C(9)	1.416(10)
C(9)-C(10)	1.348(11)
C(4)-S(1)-S(2)	102.4(3)
S(3)-S(2)-S(1)	104.66(11)
S(2)-S(3)-S(4)	105.73(11)
S(3)-S(4)-S(5)	103.83(13)
C(5)-S(5)-S(4)	104.3(3)
C(3)-O(1)-C(2)	112.4(6)
C(3)-N(1)-C(10)	127.8(7)
C(3)-N(1)-C(6)	109.4(6)
C(10)-N(1)-C(6)	122.8(6)
O(1)-C(2)-C(1)	107.8(7)
O(1)-C(3)-N(1)	120.7(7)
O(1)-C(3)-C(4)	130.7(7)
N(1)-C(3)-C(4)	108.6(7)
C(3)-C(4)-C(5)	107.6(7)
C(3)-C(4)-S(1)	125.7(6)
C(5)-C(4)-S(1)	126.6(6)

C(6)-C(5)-C(4)	107.2(6)
C(6)-C(5)-S(5)	124.6(6)
C(4)-C(5)-S(5)	128.1(6)
C(5)-C(6)-N(1)	107.1(6)
C(5)-C(6)-C(7)	134.7(7)
N(1)-C(6)-C(7)	118.1(6)
C(8)-C(7)-C(6)	119.9(7)
C(7)-C(8)-C(9)	118.6(7)
C(10)-C(9)-C(8)	123.0(7)
C(10)-C(9)-Br(1)	118.9(6)
C(8)-C(9)-Br(1)	118.1(6)
C(9)-C(10)-N(1)	117.5(7)

Anisotropic displacement parameters (A^2 x 10^3) for fw-02-01.

The anisotropic displacement factor exponent takes the form:

-2 pi^2 [h^2 a*^2 U11 + ... + 2 h k a* b* U12]

	U11	U22	U33	U23	U13	U12
Br(1)	34(1)	38(1)	42(1)	4(1)	0(1)	-9(1)
S(1)	31(1)	27(1)	29(1)	3(1)	-1(1)	1(1)
S(2)	38(1)	30(1)	33(1)	-1(1)	2(1)	2(1)
S(3)	39(1)	32(1)	35(1)	1(1)	-2(1)	-7(1)
S(4)	36(1)	36(1)	33(1)	4(1)	-6(1)	-6(1)
S(5)	25(1)	32(1)	38(1)	2(1)	-1(1)	0(1)
O(1)	28(3)	38(3)	29(3)	2(2)	-2(2)	3(2)
N(1)	30(3)	28(3)	26(4)	1(3)	-3(3)	-4(3)
C(1)	35(4)	41(4)	45(6)	-1(4)	-5(4)	8(3)
C(2)	33(4)	40(4)	31(5)	-8(4)	0(4)	6(3)
C(3)	28(4)	34(4)	26(4)	1(3)	-1(3)	7(3)
C(4)	35(4)	30(4)	26(4)	-2(3)	1(3)	-3(3)
C(5)	25(4)	27(3)	28(4)	-1(3)	-2(3)	-3(3)
C(6)	24(4)	28(3)	28(4)	-1(3)	2(3)	0(3)
C(7)	26(3)	32(3)	31(4)	-5(4)	4(4)	-1(3)
C(8)	32(4)	29(4)	25(5)	7(3)	1(3)	3(3)
C(9)	25(4)	37(4)	39(5)	-3(3)	7(3)	-4(3)
C(10)	28(4)	30(4)	29(4)	-3(4)	-1(4)	-3(3)

Hydrogen coordinates (x 10^4) and isotropic

displacement parameters (A^2 x 10^3) for fw-02-01.

	x	y	z	U(eq)
H(1A)	8698	3551	6693	60
H(1B)	8712	1834	5998	60
H(1C)	8351	2186	7710	60
H(2A)	7856	3250	4648	41
H(2B)	7505	1895	5687	41
H(7)	5576	7282	2778	36
H(8)	6485	8921	2121	35
H(10)	7773	6517	5084	35

Hydrogen bonds for fw-02-01 [A and deg.].

D-H...A	d(D-H)	d(H...A)	d(D...A)	<(DHA)
C(2)-H(2A)...O(1)#1	0.99	2.55	3.489(10)	159.3
C(7)-H(7)...S(1)#2	0.95	2.91	3.840(7)	165.7

Symmetry transformations used to generate equivalent atoms:

#1 -x+3/2,y,z-1/2 #2 -x+1,-y+1,z-1/2

Table S12. Crystal data and structure refinement for 6-ethoxy-9-iodo-[1,2,3,4,5]pentathiepino[6,7-a]indolizine (**3e**).

Identification code	RT125
Empirical formula	$C_{10}H_8NIS_5O$
Formula weight	445.37
Temperature	100(2) K
Wavelength	1.54184 A
Crystal system, space group	Orthorombic, P c a 21
Unit cell dimensions	a = 19.6206(3) A alpha = 90 deg.
	b = 8.82190(10) A beta = 90 deg.
	c = 8.23530(10) A gamma = 90 deg.
Volume	1425.46(3) A^3
Z, calculated density	4, 2.075 Mg/m^3
Absorption coefficient	24.394 mm^-1
F(000)	864
Crystal size	0.207 x 0.100 x 0.049 mm
Theta range for data collection	4.507 to 74.498 deg.
Limiting indices	-24<=h<=24, -11<=k<=10, -8<=l<=10
Reflections collected / unique	15433 / 2509 [R(int) = 0.0631]
Completeness to theta =	0.770 0.0 %
Absorption correction	Numerical
Max. and min. transmission	0.48 and 0.09
Refinement method	Full-matrix least-squares on F^2
Data / restraints / parameters	2509 / 1 / 165
Goodness-of-fit on F^2	1.156
Final R indices [I>2sigma(I)]	R1 = 0.0424, wR2 = 0.1236
R indices (all data)	R1 = 0.0426, wR2 = 0.1237
Absolute structure parameter	0.057(15)
Extinction coefficient	n/a
Largest diff. peak and hole	2.280 and -0.967 e.A^-3

Atomic coordinates (x 10^4) and equivalent isotropic

displacement parameters (A^2 x 10^3) for RT125.

U(eq) is defined as one third of the trace of the orthogonalized

Uij tensor.

	x	y	z	U(eq)
I(1)	2073(1)	4410(1)	7787(1)	24(1)
S(1)	4124(1)	-2738(3)	3810(3)	17(1)
S(2)	4248(1)	-4391(3)	5585(4)	21(1)
S(3)	5242(1)	-4143(3)	6350(4)	22(1)
S(4)	5235(1)	-2292(2)	7888(4)	22(1)
S(5)	5173(1)	-455(3)	6332(4)	19(1)
O(1)	2650(3)	-927(8)	4202(10)	19(1)
N(1)	3241(4)	714(9)	5944(12)	16(2)
C(1)	3229(5)	-535(10)	4967(14)	16(2)
C(2)	3878(5)	-1167(10)	4932(13)	15(2)
C(3)	4298(5)	-236(10)	5941(13)	15(2)
C(4)	3897(5)	915(11)	6603(13)	15(2)
C(5)	4008(5)	2100(10)	7682(16)	19(2)
C(6)	3482(5)	3083(10)	8063(14)	19(2)
C(7)	2828(5)	2866(12)	7331(14)	19(2)
C(8)	2712(5)	1700(11)	6302(13)	18(2)
C(9)	2251(5)	-2017(12)	5177(14)	20(2)
C(10)	1583(5)	-2262(13)	4299(16)	27(2)

Bond lengths [A] and angles [deg] for RT125.

I(1)-C(7)	2.047(10)
S(1)-C(2)	1.734(10)
S(1)-S(2)	2.079(4)
S(2)-S(3)	2.060(4)
S(3)-S(4)	2.067(4)
S(4)-S(5)	2.069(4)
S(5)-C(3)	1.758(10)
O(1)-C(1)	1.344(13)
O(1)-C(9)	1.477(12)
N(1)-C(1)	1.364(13)
N(1)-C(8)	1.386(12)
N(1)-C(4)	1.407(12)
C(1)-C(2)	1.391(13)
C(2)-C(3)	1.430(13)
C(3)-C(4)	1.396(13)
C(4)-C(5)	1.389(15)
C(5)-C(6)	1.385(13)
C(6)-C(7)	1.430(14)
C(7)-C(8)	1.352(16)
C(9)-C(10)	1.513(14)
C(2)-S(1)-S(2)	102.6(4)
S(3)-S(2)-S(1)	104.56(14)
S(2)-S(3)-S(4)	105.40(14)
S(3)-S(4)-S(5)	103.86(18)
C(3)-S(5)-S(4)	104.9(3)
C(1)-O(1)-C(9)	111.1(8)
C(1)-N(1)-C(8)	128.2(9)
C(1)-N(1)-C(4)	110.2(8)
C(8)-N(1)-C(4)	121.6(9)
O(1)-C(1)-N(1)	120.0(9)
O(1)-C(1)-C(2)	131.4(9)
N(1)-C(1)-C(2)	108.6(9)
C(1)-C(2)-C(3)	106.6(8)
C(1)-C(2)-S(1)	125.9(8)
C(3)-C(2)-S(1)	127.4(7)
C(4)-C(3)-C(2)	108.6(8)

C(4)-C(3)-S(5)	124.0(8)
C(2)-C(3)-S(5)	127.3(7)
C(5)-C(4)-C(3)	135.1(9)
C(5)-C(4)-N(1)	119.0(9)
C(3)-C(4)-N(1)	105.9(9)
C(6)-C(5)-C(4)	119.9(9)
C(5)-C(6)-C(7)	119.3(10)
C(8)-C(7)-C(6)	121.1(9)
C(8)-C(7)-I(1)	119.9(7)
C(6)-C(7)-I(1)	118.9(8)
C(7)-C(8)-N(1)	119.0(9)
O(1)-C(9)-C(10)	107.0(9)

Anisotropic displacement parameters (A^2 x 10^3) for RT125.

The anisotropic displacement factor exponent takes the form:

-2 pi^2 [h^2 a*^2 U11 + ... + 2 h k a* b* U12]

	U11	U22	U33	U23	U13	U12
I(1)	21(1)	24(1)	27(1)	0(1)	0(1)	4(1)
S(1)	16(1)	17(1)	19(1)	-1(1)	1(1)	-1(1)
S(2)	22(1)	19(1)	21(1)	2(1)	2(1)	0(1)
S(3)	21(1)	19(1)	25(1)	0(1)	-1(1)	6(1)
S(4)	21(1)	22(1)	23(1)	-3(1)	-3(1)	5(1)
S(5)	12(1)	19(1)	27(2)	-2(1)	0(1)	-2(1)
O(1)	16(3)	25(3)	16(4)	0(3)	-2(3)	-2(3)
N(1)	16(4)	14(4)	18(5)	1(3)	-1(3)	1(3)
C(1)	16(4)	18(4)	15(5)	-1(4)	-3(4)	1(3)
C(2)	16(4)	10(4)	19(5)	-1(4)	-1(4)	-2(3)
C(3)	16(4)	12(4)	18(5)	0(4)	3(4)	1(3)
C(4)	15(4)	12(4)	17(5)	5(4)	1(4)	0(3)
C(5)	20(4)	16(4)	22(5)	5(5)	-5(4)	-3(3)
C(6)	22(4)	17(4)	18(6)	-1(4)	-1(4)	-2(3)
C(7)	15(4)	20(5)	23(6)	2(4)	2(4)	7(3)
C(8)	16(4)	20(4)	18(5)	2(4)	2(4)	6(3)
C(9)	19(4)	24(4)	17(5)	2(4)	-2(4)	-4(4)
C(10)	16(5)	34(6)	30(7)	2(5)	-4(4)	-6(4)

Torsion angles [deg] for RT125.

C(9)-O(1)-C(1)-N(1)	-92.4(11)
C(9)-O(1)-C(1)-C(2)	89.7(14)
C(8)-N(1)-C(1)-O(1)	0.5(16)
C(4)-N(1)-C(1)-O(1)	-179.5(9)
C(8)-N(1)-C(1)-C(2)	178.8(10)
C(4)-N(1)-C(1)-C(2)	-1.2(12)
O(1)-C(1)-C(2)-C(3)	177.8(11)
N(1)-C(1)-C(2)-C(3)	-0.2(12)
O(1)-C(1)-C(2)-S(1)	0.0(18)
N(1)-C(1)-C(2)-S(1)	-178.0(8)
S(2)-S(1)-C(2)-C(1)	-108.1(9)
S(2)-S(1)-C(2)-C(3)	74.6(9)
C(1)-C(2)-C(3)-C(4)	1.6(12)
S(1)-C(2)-C(3)-C(4)	179.3(8)
C(1)-C(2)-C(3)-S(5)	-178.7(8)
S(1)-C(2)-C(3)-S(5)	-1.0(15)
S(4)-S(5)-C(3)-C(4)	105.7(9)
S(4)-S(5)-C(3)-C(2)	-74.0(10)
C(2)-C(3)-C(4)-C(5)	177.6(12)
S(5)-C(3)-C(4)-C(5)	-2.2(18)
C(2)-C(3)-C(4)-N(1)	-2.2(11)
S(5)-C(3)-C(4)-N(1)	178.0(7)
C(1)-N(1)-C(4)-C(5)	-177.7(10)
C(8)-N(1)-C(4)-C(5)	2.3(14)
C(1)-N(1)-C(4)-C(3)	2.1(11)
C(8)-N(1)-C(4)-C(3)	-177.8(9)
C(3)-C(4)-C(5)-C(6)	178.7(11)
N(1)-C(4)-C(5)-C(6)	-1.6(15)
C(4)-C(5)-C(6)-C(7)	-0.3(16)
C(5)-C(6)-C(7)-C(8)	1.5(16)
C(5)-C(6)-C(7)-I(1)	-175.9(8)
C(6)-C(7)-C(8)-N(1)	-0.8(16)
I(1)-C(7)-C(8)-N(1)	176.6(7)
C(1)-N(1)-C(8)-C(7)	178.9(10)
C(4)-N(1)-C(8)-C(7)	-1.1(15)
C(1)-O(1)-C(9)-C(10)	174.4(9)

Hydrogen bonds for RT125 [A and deg.].

D-H...A	d(D-H)	d(H...A)	d(D...A)	<(DHA)
C(9)-H(9A)...O(1)#1	0.99	2.51	3.457(14)	160.7
C(9)-H(9B)...I(1)#2	0.99	3.20	3.831(11)	123.1
C(5)-H(5)...S(1)#3	0.95	2.89	3.822(10)	166.3
C(9)-H(9B)...I(1)#4	0.99	3.19	3.946(11)	134.1

Symmetry transformations used to generate equivalent atoms:
#1 -x+1/2,y,z+1/2 #2 x,y-1,z #3 -x+1,-y,z+1/2
#4 -x+1/2,y-1,z-1/2

Table S13. Crystal data and structure refinement for 6-ethoxy-9-nitro-[1,2,3,4,5]pentathiepino[6,7-a]indolizine (**3f**).

Identification code	RT50
Empirical formula	$C_{10}H_8N_2S_5O_3$
Formula weight	364.48
Temperature	170(2) K
Wavelength	0.71073 A
Crystal system, space group	Orthorombic, P -1
Unit cell dimensions	a = 7.7568(16) A alpha = 89.31(3) deg.
	b = 9.1803(18) A beta = 78.99(3) deg.
	c = 10.401(2) A gamma = 76.65(3) deg.
Volume	707.0(3) A^3
Z, calculated density	2, 1.712 Mg/m^3
Absorption coefficient	0.825 mm^-1
F(000)	372
Crystal size	0.229 x 0.210 x 0.181 mm
Theta range for data collection	1.996 to 29.543 deg.
Limiting indices	-10<=h<=10, -12<=k<=12, -14<=l<=14
Reflections collected / unique	7776 / 3862 [R(int) = 0.0261]
Completeness to theta =	98.9 %

Absorption correction	Numerical
Max. and min. transmission	0.7981 and 0.6942
Refinement method	Full-matrix least-squares on F^2
Data / restraints / parameters	3862 / 0 / 182
Goodness-of-fit on F^2	1.112
Final R indices [I>2sigma(I)]	R1 = 0.0362, wR2 = 0.0989
R indices (all data)	R1 = 0.0483, wR2 = 0.1041
Extinction coefficient	n/a
Largest diff. peak and hole	0.882 and -0.466 e.A^-3

Table 2. Atomic coordinates (x 10^4) and equivalent isotropic displacement parameters (A^2 x 10^3) for rt50.
U(eq) is defined as one third of the trace of the orthogonalized Uij tensor.

	x	y	z	U(eq)
S(1)	5484(1)	8440(1)	3271(1)	36(1)
S(2)	5016(1)	8077(1)	5251(1)	35(1)
S(3)	7486(1)	6951(1)	5603(1)	38(1)
S(4)	7687(1)	4797(1)	5004(1)	34(1)
S(5)	8441(1)	4797(1)	2995(1)	33(1)
O(1)	2297(2)	7727(2)	1851(1)	38(1)
O(2)	663(2)	3315(2)	37(2)	46(1)
O(3)	2635(2)	1218(2)	-235(2)	45(1)
N(1)	3962(2)	5253(2)	1634(1)	25(1)
N(2)	2125(2)	2524(2)	139(2)	32(1)
C(1)	32(4)	9893(3)	2623(3)	74(1)
C(2)	937(4)	8398(3)	2941(3)	57(1)
C(3)	3724(2)	6709(2)	2074(2)	28(1)
C(4)	5193(3)	6797(2)	2607(2)	28(1)
C(5)	6371(2)	5344(2)	2500(2)	28(1)
C(6)	5608(2)	4396(2)	1893(2)	26(1)
C(7)	6100(3)	2863(2)	1518(2)	29(1)
C(8)	4987(3)	2240(2)	947(2)	31(1)
C(9)	3337(2)	3176(2)	738(2)	27(1)
C(10)	2825(2)	4652(2)	1060(2)	26(1)

Table 3. Bond lengths [A] and angles [deg] for rt50.

S(1)-C(4)	1.745(2)
S(1)-S(2)	2.0593(8)
S(2)-S(3)	2.0530(10)
S(3)-S(4)	2.0427(9)
S(4)-S(5)	2.0598(9)
S(5)-C(5)	1.7420(19)
O(1)-C(3)	1.330(2)
O(1)-C(2)	1.428(3)
O(2)-N(2)	1.219(2)
O(3)-N(2)	1.217(2)
N(1)-C(10)	1.368(2)
N(1)-C(3)	1.378(2)
N(1)-C(6)	1.409(2)
N(2)-C(9)	1.457(2)
C(1)-C(2)	1.456(4)
C(3)-C(4)	1.376(3)
C(4)-C(5)	1.425(3)
C(5)-C(6)	1.377(3)
C(6)-C(7)	1.410(3)
C(7)-C(8)	1.363(3)
C(8)-C(9)	1.421(3)
C(9)-C(10)	1.349(3)
C(4)-S(1)-S(2)	102.38(7)
S(3)-S(2)-S(1)	104.27(4)
S(4)-S(3)-S(2)	103.58(4)
S(3)-S(4)-S(5)	104.38(4)
C(5)-S(5)-S(4)	102.47(7)
C(3)-O(1)-C(2)	118.72(17)
C(10)-N(1)-C(3)	128.19(15)
C(10)-N(1)-C(6)	122.76(16)
C(3)-N(1)-C(6)	109.03(15)
O(3)-N(2)-O(2)	123.70(18)
O(3)-N(2)-C(9)	117.85(16)
O(2)-N(2)-C(9)	118.45(16)
O(1)-C(2)-C(1)	109.9(2)
O(1)-C(3)-C(4)	133.09(18)
O(1)-C(3)-N(1)	118.62(16)
C(4)-C(3)-N(1)	108.11(16)
C(3)-C(4)-C(5)	107.83(17)
C(3)-C(4)-S(1)	124.14(15)
C(5)-C(4)-S(1)	128.04(14)
C(6)-C(5)-C(4)	107.91(16)
C(6)-C(5)-S(5)	124.09(15)
C(4)-C(5)-S(5)	127.99(15)
C(5)-C(6)-N(1)	107.12(16)
C(5)-C(6)-C(7)	135.00(17)
N(1)-C(6)-C(7)	117.89(17)
C(8)-C(7)-C(6)	120.35(17)
C(7)-C(8)-C(9)	118.28(18)
C(10)-C(9)-C(8)	123.43(18)
C(10)-C(9)-N(2)	117.61(16)
C(8)-C(9)-N(2)	118.96(17)
C(9)-C(10)-N(1)	117.28(16)

Symmetry transformations used to generate equivalent atoms:

Table 4. Anisotropic displacement parameters (A^2 x 10^3) for rt50.
The anisotropic displacement factor exponent takes the form:
-2 pi^2 [h^2 a*^2 U11 + ... + 2 h k a* b* U12]

	U11	U22	U33	U23	U13	U12
S(1)	53(1)	31(1)	27(1)	1(1)	-11(1)	-15(1)
S(2)	47(1)	34(1)	26(1)	-3(1)	-7(1)	-11(1)
S(3)	44(1)	48(1)	28(1)	1(1)	-12(1)	-20(1)
S(4)	34(1)	41(1)	29(1)	7(1)	-11(1)	-11(1)
S(5)	27(1)	44(1)	29(1)	-1(1)	-6(1)	-7(1)
O(1)	40(1)	34(1)	35(1)	-6(1)	-13(1)	7(1)
O(2)	41(1)	44(1)	58(1)	-8(1)	-24(1)	-5(1)
O(3)	53(1)	31(1)	56(1)	-10(1)	-17(1)	-9(1)
N(1)	28(1)	25(1)	22(1)	-2(1)	-6(1)	-3(1)
N(2)	36(1)	32(1)	27(1)	-2(1)	-8(1)	-8(1)
C(1)	73(2)	46(2)	78(2)	-1(2)	1(2)	22(1)
C(2)	53(2)	53(2)	52(2)	0(1)	2(1)	5(1)
C(3)	32(1)	25(1)	24(1)	-1(1)	-6(1)	-1(1)
C(4)	35(1)	28(1)	22(1)	0(1)	-6(1)	-7(1)
C(5)	29(1)	31(1)	22(1)	1(1)	-5(1)	-6(1)
C(6)	27(1)	28(1)	22(1)	1(1)	-5(1)	-2(1)
C(7)	30(1)	28(1)	26(1)	-1(1)	-6(1)	0(1)
C(8)	36(1)	27(1)	26(1)	-2(1)	-6(1)	-2(1)
C(9)	31(1)	29(1)	22(1)	-2(1)	-6(1)	-7(1)
C(10)	27(1)	29(1)	22(1)	-1(1)	-5(1)	-4(1)

Table 5. Torsion angles [deg] for rt50.

C(3)-O(1)-C(2)-C(1)	152.2(2)
C(2)-O(1)-C(3)-C(4)	-71.3(3)
C(2)-O(1)-C(3)-N(1)	114.2(2)
C(10)-N(1)-C(3)-O(1)	-5.5(3)
C(6)-N(1)-C(3)-O(1)	175.96(17)
C(10)-N(1)-C(3)-C(4)	178.72(17)
C(6)-N(1)-C(3)-C(4)	0.2(2)
O(1)-C(3)-C(4)-C(5)	-175.3(2)
N(1)-C(3)-C(4)-C(5)	-0.4(2)
O(1)-C(3)-C(4)-S(1)	4.3(3)
N(1)-C(3)-C(4)-S(1)	179.21(13)
S(2)-S(1)-C(4)-C(3)	107.12(17)
S(2)-S(1)-C(4)-C(5)	-73.34(18)
C(3)-C(4)-C(5)-C(6)	0.5(2)
S(1)-C(4)-C(5)-C(6)	-179.09(14)
C(3)-C(4)-C(5)-S(5)	179.27(14)
S(1)-C(4)-C(5)-S(5)	-0.3(3)
S(4)-S(5)-C(5)-C(6)	-107.74(16)
S(4)-S(5)-C(5)-C(4)	73.68(18)
C(4)-C(5)-C(6)-N(1)	-0.4(2)
S(5)-C(5)-C(6)-N(1)	-179.22(13)
C(4)-C(5)-C(6)-C(7)	-180.0(2)
S(5)-C(5)-C(6)-C(7)	1.2(3)
C(10)-N(1)-C(6)-C(5)	-178.50(16)
C(3)-N(1)-C(6)-C(5)	0.2(2)
C(10)-N(1)-C(6)-C(7)	1.2(3)
C(3)-N(1)-C(6)-C(7)	179.80(16)
C(5)-C(6)-C(7)-C(8)	178.3(2)
N(1)-C(6)-C(7)-C(8)	-1.3(3)
C(6)-C(7)-C(8)-C(9)	0.3(3)
C(7)-C(8)-C(9)-C(10)	0.9(3)
C(7)-C(8)-C(9)-N(2)	-179.47(17)
O(3)-N(2)-C(9)-C(10)	175.07(18)
O(2)-N(2)-C(9)-C(10)	-4.8(3)
O(3)-N(2)-C(9)-C(8)	-4.6(3)
O(2)-N(2)-C(9)-C(8)	175.56(18)
C(8)-C(9)-C(10)-N(1)	-1.0(3)
N(2)-C(9)-C(10)-N(1)	179.35(15)
C(3)-N(1)-C(10)-C(9)	-178.44(17)
C(6)-N(1)-C(10)-C(9)	-0.1(3)

Table S14. Crystal data and structure refinement for 6-ethoxy-9-methyl-[1,2,3,4,5]pentathiepino[6,7-a]indolizine (**3g**).

Identification code	RT160
Empirical formula	$C_{11}H_{11}NS_5O$
Formula weight	333.51
Temperature	103(2) K
Wavelength	1.54184 A
Crystal system, space group	Orthorhombic, P c a 21
Unit cell dimensions	a = 19.29930(10) A alpha = 90 deg.
	b = 8.63970(10) A beta = 90 deg.
	c = 8.25520(10) A gamma = 90 deg.
Volume	1376.47(2) A^3
Z, calculated value	4, 1.609 Mg/m^3
Absorption coefficient	7.649 mm^-1
F(000)	688
Crystal size	0.130 x 0.090 x 0.050 mm
Theta range for data collection	4.582 to 79.698 deg.
Limiting indices	-24<=h<=24, -11<=k<=10, -10<=l<=10
Reflections collected / unique	21710 / 2728 [R(int) = 0.0463]
Completeness to theta =	0.0 %
Absorption correction	Numerical
Max. and min. transmission	1.000 and 0.804
Refinement method	Full-matrix least-squares on F^2
Data / restraints / parameters	2728 / 1 / 165
Goodness-of-fit on F^2	1.083
Final R indices [I>2sigma(I)]	R1 = 0.0318, wR2 = 0.0897
R indices (all data)	R1 = 0.0320, wR2 = 0.0899
Absolute structure parameter	-0.01(2)
Extinction coefficient	n/a
Largest diff. peak and hole	0.367 and -0.284 e.A^-3

Table 2. Atomic coordinates (x 10^4) and equivalent isotropic displacement parameters (A^2 x 10^3) for rt160.
U(eq) is defined as one third of the trace of the orthogonalized Uij tensor.

	x	y	z	U(eq)
S(1)	5904(1)	2257(1)	2808(1)	16(1)
S(2)	5785(1)	558(1)	4546(1)	19(1)
S(3)	4777(1)	787(1)	5329(1)	20(1)
S(4)	4765(1)	2673(1)	6875(1)	20(1)
S(5)	4838(1)	4569(1)	5345(1)	17(1)
O(1)	7409(1)	4002(2)	3319(3)	18(1)
N(1)	6812(1)	5709(3)	5065(4)	16(1)
C(1)	6815(2)	4445(3)	4051(4)	16(1)
C(2)	6151(2)	3856(3)	3980(4)	16(1)
C(3)	5722(1)	4777(3)	5002(4)	15(1)
C(4)	6143(2)	5925(3)	5680(4)	15(1)
C(5)	6039(2)	7154(3)	6787(4)	17(1)
C(6)	6581(2)	8089(3)	7183(4)	18(1)
C(7)	7257(2)	7867(3)	6494(4)	18(1)
C(8)	7362(1)	6669(3)	5466(4)	17(1)
C(9)	7842(2)	8957(4)	6914(4)	23(1)
C(10)	7786(2)	2834(3)	4254(4)	21(1)
C(11)	8464(2)	2578(4)	3411(5)	23(1)

Table 3. Bond lengths [A] and angles [deg] for rt160.

S(1)-C(2)	1.753(3)
S(1)-S(2)	2.0653(10)
S(2)-S(3)	2.0586(11)
S(3)-S(4)	2.0698(10)
S(4)-S(5)	2.0737(11)
S(5)-C(3)	1.740(3)
O(1)-C(1)	1.352(4)
O(1)-C(10)	1.464(4)
N(1)-C(1)	1.376(4)
N(1)-C(8)	1.387(4)
N(1)-C(4)	1.400(4)
C(1)-C(2)	1.379(4)
C(2)-C(3)	1.425(4)
C(3)-C(4)	1.398(4)
C(4)-C(5)	1.416(4)
C(5)-C(6)	1.362(4)
C(6)-C(7)	1.436(5)
C(7)-C(8)	1.354(5)
C(7)-C(9)	1.510(4)
C(10)-C(11)	1.498(5)
C(2)-S(1)-S(2)	101.99(11)
S(3)-S(2)-S(1)	104.75(4)
S(2)-S(3)-S(4)	106.29(4)
S(3)-S(4)-S(5)	104.21(5)
C(3)-S(5)-S(4)	104.32(10)
C(1)-O(1)-C(10)	112.4(2)
C(1)-N(1)-C(8)	128.0(3)
C(1)-N(1)-C(4)	109.2(2)
C(8)-N(1)-C(4)	122.7(3)
O(1)-C(1)-N(1)	120.0(3)
O(1)-C(1)-C(2)	131.6(3)
N(1)-C(1)-C(2)	108.4(3)
C(1)-C(2)-C(3)	107.9(3)
C(1)-C(2)-S(1)	124.6(2)
C(3)-C(2)-S(1)	127.5(2)

C(4)-C(3)-C(2)	107.2(3)
C(4)-C(3)-S(5)	125.3(2)
C(2)-C(3)-S(5)	127.5(2)
C(3)-C(4)-N(1)	107.2(2)
C(3)-C(4)-C(5)	135.1(3)
N(1)-C(4)-C(5)	117.6(3)
C(6)-C(5)-C(4)	119.5(3)
C(5)-C(6)-C(7)	121.6(3)
C(8)-C(7)-C(6)	119.1(3)
C(8)-C(7)-C(9)	120.6(3)
C(6)-C(7)-C(9)	120.3(3)
C(7)-C(8)-N(1)	119.5(3)
O(1)-C(10)-C(11)	106.9(3)

Symmetry transformations used to generate equivalent atoms:

Table 4. Anisotropic displacement parameters (A^2 x 10^3) for rt160.

The anisotropic displacement factor exponent takes the form:

-2 pi^2 [h^2 a*^2 U11 + ... + 2 h k a* b* U12]

	U11	U22	U33	U23	U13	U12
S(1)	19(1)	12(1)	18(1)	-1(1)	0(1)	0(1)
S(2)	22(1)	12(1)	22(1)	1(1)	-2(1)	2(1)
S(3)	22(1)	14(1)	24(1)	0(1)	1(1)	-4(1)
S(4)	20(1)	16(1)	23(1)	-1(1)	3(1)	-4(1)
S(5)	14(1)	14(1)	25(1)	-1(1)	0(1)	0(1)
O(1)	18(1)	16(1)	20(1)	1(1)	3(1)	2(1)
N(1)	16(1)	11(1)	20(1)	1(1)	2(1)	0(1)
C(1)	17(1)	14(1)	16(1)	0(1)	1(1)	1(1)
C(2)	17(1)	12(1)	20(1)	-1(1)	1(1)	0(1)
C(3)	15(1)	11(1)	20(2)	1(1)	-1(1)	1(1)
C(4)	15(1)	11(1)	18(1)	2(1)	2(1)	1(1)
C(5)	19(1)	12(1)	21(2)	2(1)	2(1)	1(1)
C(6)	22(1)	12(1)	20(1)	-2(1)	-2(1)	1(1)
C(7)	19(1)	13(1)	21(2)	2(1)	-2(1)	-3(1)
C(8)	16(1)	15(1)	21(2)	2(1)	0(1)	-2(1)
C(9)	23(2)	19(1)	28(2)	-1(1)	-4(1)	-8(1)
C(10)	19(1)	20(1)	23(2)	1(1)	3(1)	5(1)
C(11)	19(2)	25(1)	26(2)	0(1)	2(1)	4(1)

Table 5. Torsion angles [deg] for rt160.

C(10)-O(1)-C(1)-N(1)	92.7(3)
C(10)-O(1)-C(1)-C(2)	-88.0(4)
C(8)-N(1)-C(1)-O(1)	0.6(5)
C(4)-N(1)-C(1)-O(1)	-179.5(3)
C(8)-N(1)-C(1)-C(2)	-178.8(3)
C(4)-N(1)-C(1)-C(2)	1.1(3)
O(1)-C(1)-C(2)-C(3)	179.9(3)
N(1)-C(1)-C(2)-C(3)	-0.7(3)
O(1)-C(1)-C(2)-S(1)	-0.4(5)
N(1)-C(1)-C(2)-S(1)	179.0(2)
S(2)-S(1)-C(2)-C(1)	105.7(3)
S(2)-S(1)-C(2)-C(3)	-74.7(3)
C(1)-C(2)-C(3)-C(4)	0.1(4)
S(1)-C(2)-C(3)-C(4)	-179.6(2)
C(1)-C(2)-C(3)-S(5)	179.0(2)
S(1)-C(2)-C(3)-S(5)	-0.6(4)
S(4)-S(5)-C(3)-C(4)	-105.6(3)
S(4)-S(5)-C(3)-C(2)	75.6(3)
C(2)-C(3)-C(4)-N(1)	0.5(3)
S(5)-C(3)-C(4)-N(1)	-178.4(2)
C(2)-C(3)-C(4)-C(5)	-178.7(3)
S(5)-C(3)-C(4)-C(5)	2.3(5)
C(1)-N(1)-C(4)-C(3)	-1.0(3)
C(8)-N(1)-C(4)-C(3)	178.9(3)
C(1)-N(1)-C(4)-C(5)	178.4(3)
C(8)-N(1)-C(4)-C(5)	-1.7(4)
C(3)-C(4)-C(5)-C(6)	-179.5(3)
N(1)-C(4)-C(5)-C(6)	1.3(4)
C(4)-C(5)-C(6)-C(7)	0.5(5)
C(5)-C(6)-C(7)-C(8)	-2.2(5)
C(5)-C(6)-C(7)-C(9)	177.8(3)
C(6)-C(7)-C(8)-N(1)	1.8(5)
C(9)-C(7)-C(8)-N(1)	-178.2(3)
C(1)-N(1)-C(8)-C(7)	180.0(3)
C(4)-N(1)-C(8)-C(7)	0.1(5)
C(1)-O(1)-C(10)-C(11)	-173.9(3)

*Synthesis of precursor **1b***

The 5-fluoro-2-brompyridine derivative was obtained following a classic Sandmeyer approach. A diazonium tetrafluoborate salt from 5-amino-2-bromopyridine was isolated as reported in the literature, then heated up in hexane up to a maximum of 40 °C to give the desired product. Trials at 80 °C in toluene or boiling hexane resulted in unsatisfactory yields and the formation of numerous byproducts. Gentle heating was the key to reach an acceptable yield. The reaction (Figure S52) can be followed by TLC and its initiation is immediately noticeable since molecular nitrogen is formed and gas bubbles become visible. Product formation was supported by mass spectrometry and the compound was used for the next step without further purification.

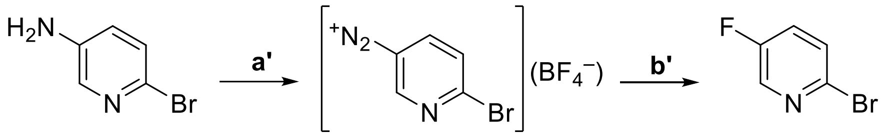

4 **5** **1b**

a': HBF_4, $NaNO_2$, EtOH, H_2O, 0 °C to RT, 30 min
b': Hexane, 40 °C, overnight. Yield 57%.

Figure S52. Synthesis of **1b** through a diazonium salt intermediate followed by a Sandmeyer reaction.
